# Genome-Wide Identification and Expression Profiling of *SlGeBP* Gene Family in Response to Hormone and Abiotic Stresses in *Solanum lycopersicum* L.

**DOI:** 10.3390/ijms26136008

**Published:** 2025-06-23

**Authors:** Haohao Cao, Danfeng Wang, Xiaoli Li, Yi Zhang, Deding Su, Wang Lu, Kedong Xu, Zhengguo Li

**Affiliations:** 1 Key Laboratory of Crop Molecular Breeding and Bioreactor of Henan, Zhou Kou Normal University, Zhoukou 466000, China; haohaocao@zknu.edu.cn (H.C.); 20211046@zknu.edu.cn (D.W.); lxl1989@zknu.edu.cn (X.L.); 20091018@zknu.edu.cn (Y.Z.); 2 Key Laboratory of Southwest China Wildlife Resources Conservation (Ministry of Education), School of Life Science, China West Normal University, Nanchong 637001, China; dedingsu@cwnu.edu.cn; 3 Key Laboratory of Plant Hormones Regulation and Molecular Breeding of Chongqing, School of Life Sciences, Chongqing University, Chongqing 401331, China; 20192601787@stu.cqu.edu.cn; 4 Center of Plant Functional Genomics and Synthetic Biology, Institute of Advanced Interdisciplinary Studies, Chongqing University, Chongqing 401331, China

**Keywords:** tomato, GeBP gene family, genome-wide analysis, hormone and stress, potential function, abiotic stress

## Abstract

The *GLABROUS1* enhancer-binding protein (GeBP) gene family, a plant-specific class of transcriptional regulators, is involved in multiple biological processes, including the formation of trichomes, plant growth, and environmental adaptation. However, the functional characterization of *SlGeBP* genes in tomato remains poor, particularly regarding their roles in regulating developmental processes and stress response mechanisms. In this study, 11 *SlGeBP* family members were identified from the tomato genome and 97 GeBP proteins from six species were classified into three groups. A wide range of elements linked to phytohormone, stress, and plant development were presented on the promoter sequences. Gene expression profile analysis revealed a comprehensive expression during the vegetative and immature fruit development stages. Analysis of the expression level under nine hormones and seven stresses can help us to understand the responsiveness of *SlGeBP* genes associated with hormone induction and stress tolerance. Subcellular localization analysis exhibited that SlGeBP1 and SlGeBP5 were localized in the nucleus, and the yeast two-hybrid assay confirmed that SlGeBP1 could interact with SlGeBP5. This study will help us to understand the potential function of the *SlGeBP* family and may establish a basis for further research on phytohormone signaling and stress resistance.

## 1. Introduction

Trichomes are appendages growing on the surfaces of numerous land plants. They are special structures that derive from the development of epidermal cells and involve the complex regulation of cell cycle, transcription, and cytoskeletal functions [1]. Trichomes constitute specialized multicellular epidermal structures that bifurcate into glandular and non-glandular types [2]. Through coordinated mechanical barriers and immune responsive metabolic pathways, these structures biosynthesize specialized defensive metabolites that effectively deter phytopathogens and herbivorous arthropods [3,4,5,6].

The *GLABROUS1* enhancer-binding protein (GeBP) transcription factors are a plant-specific transcription factor family composed of a basic amino acid central region and a non-typical leucine zipper region [7]. As the distance between these two conserved regions is greater than nine residues—distinct from the standard bZIP protein—the GeBP transcription factor family is considered as a novel family of transcription factors [8,9,10,11].

Previous studies have reported that there were 23, 7, 9,13, 20, and 6 GeBP family members in *Arabidopsis thaliana* L. [7], apple [9], soybean [10], rice [11], *Brassica rapa* L. [12], and tea plant [13], respectively. They have been proven to participate in plant growth, the formation of plant trichomes, plant senescence, and the response to environmental stresses [7,8,9,10,11,12,13,14]. Chevalier et al. reported that AtGeBP can interact with AtGL1 to regulate trichome formation [7]. In tea plant and soybean, a number of genes have also been discovered to be involved in trichomes’ formation, such as *CsGeBP4* and *GmGeBP4* [10,13]. In apple, the ectopic overexpression of the *MdGeBP3* can promote adventitious root development, as well as delayed flowering [9]. Furthermore, the role of GeBP’s participation in response to hormones has already been reported. During the process of plant growth and senescence, the functional loss of the triple mutants *gebp*/*gpl1*/*gpl2* indicates a reduced sensitivity to exogenous cytokinins [7]. The *gebp*/*gpl1*/*gpl2* mutant lines exhibited a significantly increased expression of type-A response regulators (RRs), which act as a negative feedback regulator involved in the signal transduction of cytokinin. *MdGeBP3-OX* seeding could be involved in cytokinin-responsive regulation and a decreased sensitivity to ABA [9].

Additionally, related research has further exhibited that GeBP critically regulates stress-responsive processes. Studies have shown that the transcription factor *AtGPL4* (*GeBP-like 4*) acts as a negative regulatory factor of root growth that is rapidly responsive to heavy metals such as cadmium (Cd),copper (Cu), and zinc (Zn) [8]. In *Brassica rapa*, the expression levels of *BrGeBP3*, *BrGeBP14,* and *BrGeBP17* have also been reported to be significantly upregulated under drought stress. BrGeBP3 and BrGeBP14, functional homologs of *Arabidopsis* At5g28040, interact with DES1 to promote H_2_S biosynthesis. This H_2_S-mediated pathway modulates SnRK2.6-regulated ABA signaling, ultimately accelerating stomatal closure to enhance stress tolerance [12]. The ectopic expression of *MdGeBP3* induces drought hypersensitivity in *Arabidopsis* through dual mechanisms: accelerated chlorophyll catabolism and a pathological accumulation of reactive oxygen species (H_2_O_2_ and O_2_). This oxidative burst directly compromises membrane integrity, establishing *MdGeBP3* as a negative regulator of drought tolerance [9].

As a model crop for genetic research, tomato offers unique advantages for genes’ functional characterization due to its abundant genomic resources and large-scale EMS mutant library [15,16,17]. In recent years, the rapid advancement in CRISPR/Cas gene-editing technology has enabled the precise knockout, overexpression, or single-base editing of target genes, significantly accelerating the study of the genetic regulatory mechanisms underlying key agronomic traits such as stress tolerance, yield, and fruit quality [18,19,20,21]. Notably, in some species, including *Arabidopsis*, *Brassica rapa,* and *Malus domestica* L., members of the *GeBP* gene have been shown to broadly participate in stress responses such as drought cold and heat stress. In addition, *AtGeBP*, *CsGeBP4,* and *GmGeBP4* have been reported to regulate trichome development, which may enhance physical and chemical defenses against pathogen and pest infestation. The growth and ripening process of tomato is finely regulated by a variety of hormones, and this process is also subject to various external environmental stresses, such as drought and wound stress [22,23,24,25,26]. In tomato, the *SlGeBP* gene family—as homologs to *Arabidopsis*—may have inherited conserved biological functions, while also potentially acquiring novel functions through species-specific evolution, thereby enabling adaptation to diverse ecological niches and developmental demands. Therefore, systematic analysis of the molecular features of the *SlGeBP* gene family members and their responses to hormonal and abiotic stress conditions will help to uncover their regulatory nodes within the plant stress adaptation network, providing critical targeted editing sites for the molecular breeding of stress-tolerant cultivars.

In this research, a comprehensive genome-wide analysis of the *SlGeBP* transcription factor family was achieved using bioinformatic methods. Public transcriptome data and qRT-PCR were employed to analyze the expression pattern of *SlGeBP* transcription factors during different stages in tomato. At the same time, the responsiveness of the *SlGeBP* transcription factors was tested under nine significant plant hormones and seven different stresses. We also predicted their interacting proteins and proposed hypotheses regarding the molecular regulatory mechanisms of SlGeBP1 and SlGeBP5 in response to drought and temperature stresses. This work aimed to provide insights into the potential functions of *SlGeBP* genes in phytohormone signaling and stress resistance, which could be valuable for molecular breeding in the future.

## 2. Results

### 2.1. Identification and Physical and Chemical Parameters of SlGeBP Gene Family in Tomato

Through a comprehensive screening and sequence alignment, eleven members of the *SlGeBP* gene family were successfully identified in tomato. These were further validated using the NCBI CD-Search tool and the Pfam database, ensuring the accuracy and reliability of the findings.

Based on the genomic position, the 11 *SlGeBPs* were termed *SlGeBP1* to *SlGeBP11*. The number of exons in the *SlGeBP* genes was relatively low, with most of them having one exon, except for *SlGeBP8,* which contained two exons, and *SlGeBP2,* which contained eight exons. The lengths of the SlGeBP proteins ranged from 157 (SlGeBP10) to 519 (SlGeBP2) AA, and the predicted molecular weight (MW) ranged from 18,184.97 (SlGeBP4) to 57,645.42 (SlGeBP2). The isoelectric point (pI) values of SlGeBP2, SlGeBP3, and SlGeBP4 were beyond 7, revealing that the proteins were basic, while the other eight proteins were acidic. The aliphatic index (AI) values of the SlGeBP proteins ranged from 58.14 (SlGeBP10) to 85.08 (SlGeBP2), indicating that they have a higher thermostability. The grand average of hydropathicity (GRAVY) ranged from −1.295 (SlGeBP10) to −0.371 (SlGeBP1), suggesting a strong hydrophilicity. Some other detailed data, such as the gene accession number and genomic locus, have detailed annotations in Table 1.

### 2.2. Chromosome Distribution and Multiple Sequence Alignment Analysis of SlGeBP Genes

**Figure 1:**
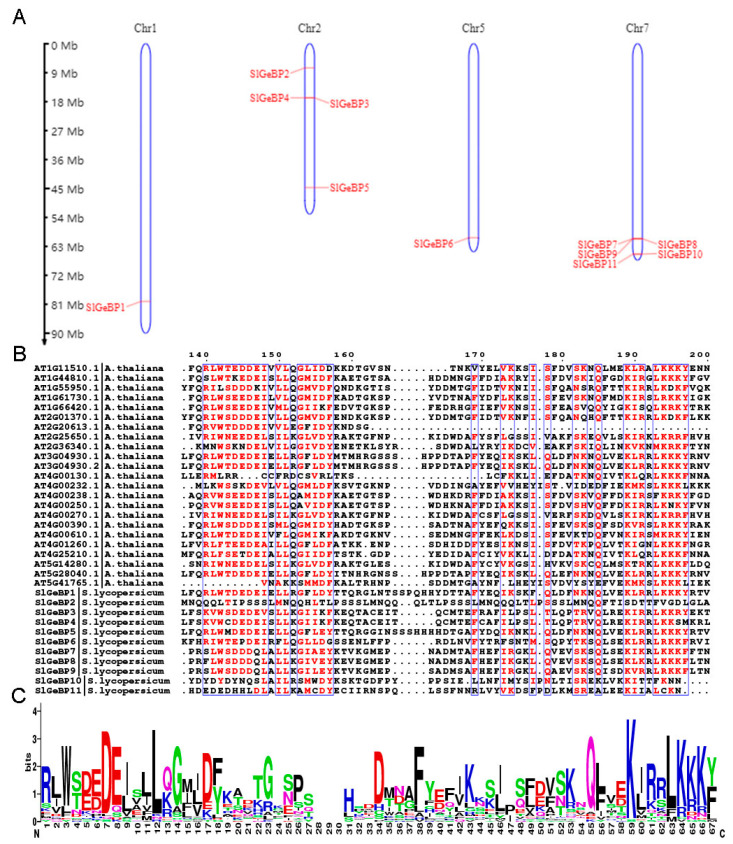
Chromosome location and amino acid multiple sequence alignment of GeBPs. (**A**) Chromosome location of the eleven *SlGeBP* genes, with the red line demonstrating their location on the chromosome. (**B**) Multiple amino acid sequence alignment of GeBP proteins in *Arabidopsis* and tomato. (**C**) Sequence logos of amino acid multiple sequence alignment of AtGeBP and SlGeBP.

The *SlGeBP* gene family was distributed into four chromosomes, with only one gene located on chromosome 1 and chromosome 5, four genes located on chromosome 2, and five genes located on chromosome 7 (Figure 1A). In this group, six *SlGeBP* genes were sense strand, whereas the other five genes were antisense strand (Table 1). Based on the alignment of 23 *Arabidopsis* and 11 tomato GeBP family members, the conserved sequences are mainly located at the C-terminal, consisting of approximately 60 conserved amino acids, which contain a relatively high number of asparagine, leucine, and lysine residues (Figure 1B,C). Furthermore, they have highly similar conserved sequences between *Arabidopsis* and tomato, also indicating that the sequences were relatively conserved during evolution (Figure S1).

### 2.3. Spatial Structure and Phylogenetic Analysis of SlGeBP Genes

The spatial structure prediction for SlGeBP proteins demonstrated that they were different from each other, as well as the existence of a helix, strand, and random coil in the protein structure (Table S1). According to outcomes from four subcellular localization prediction online tools, almost all SlGeBP proteins were presumptively located in the nucleus, except for SlGeBP3, which may be located in the nucleus or cytoplasm; SlGeBP4, which may be located in the chloroplast or nucleus; and SlGeBP6, which may be located in the plastid or nucleus (Table S2).

**Figure 2:**
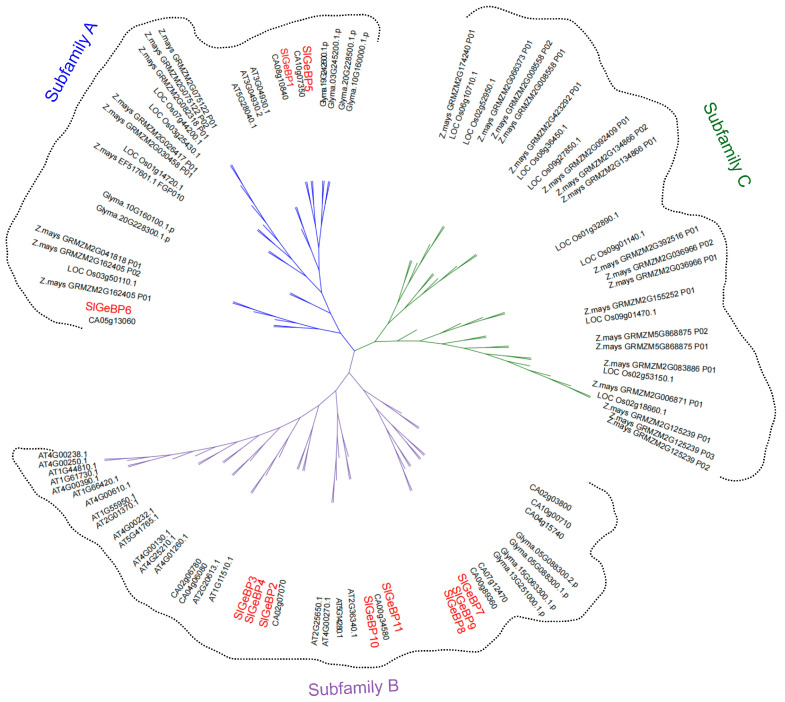
Phylogenetic analysis of GeBP family. A total of 97 GeBP family members from 6 different species within rice, maize, *Arabidopsis,* pepper, soybean, and tomato (red font). The phylogenetic tree was constructed with the NJ method through MEGA X with 1000 bootstrap replications. iTOL v7 was implemented for the annotation, management and formation of an unrooted phylogenetic tree.

A total of 97 GeBP family members from six different species, including monocotyledonous plants such as rice [11] and maize [27], as well as dicotyledonous plants, including *Arabidopsis* [7,9], pepper [27], soybean [9,27], and tomato [11], were used to analyze their phylogenetic relationship through the neighbor joining method. The GeBP members were classified into three subfamily groups, designated as subfamilies A, B, and C (Figure 2). Among them, subfamily B was the largest, containing 41 members, followed by subfamilies A and C, each containing 28 members. Subfamily A contained three members of GeBP in tomato, while subfamily C did not include any members from tomato. Notably, subfamily C was exclusively composed of GeBP members from monocotyledonous plants. Subfamily B was composed of 8 SlGeBPs, 20 AtGeBPs, 9 CaGeBPs, and 4 GmGeBPs, indicating a closer evolutionary relationship with *A. thaliana* and *C. annuum* than *G.max, O. sativa,* or *Z. mays.*

### 2.4. Gene Structure, Conserved Motif, and Collinearity Analysis of SlGeBP Genes

**Figure 3:**
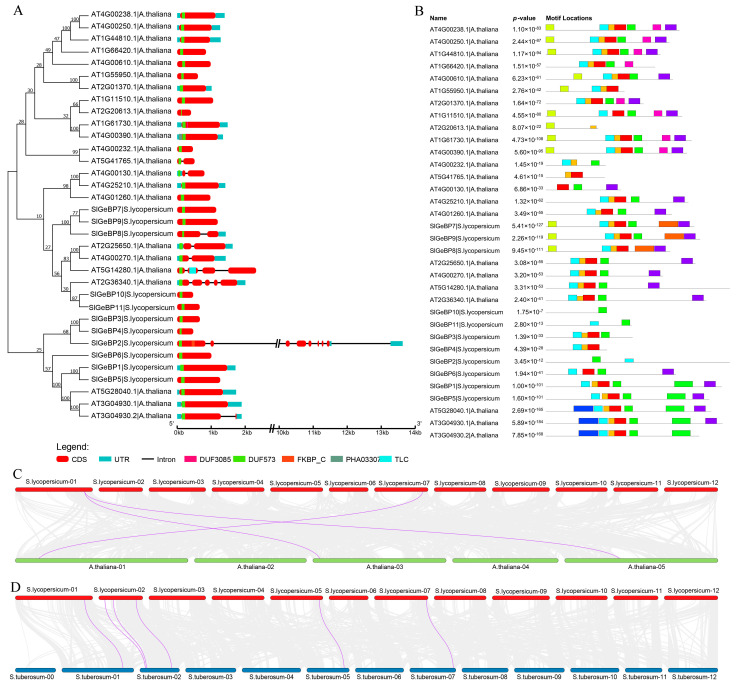
Gene structure, conserved motif, and collinearity analysis of *SlGeBP* genes. (**A**) The NJ tree on the left side was constructed by MEGA X from *Arabidopsis* and tomato. The gene structure, including the quantities and position of introns, exons, and functional domains, was visualized by GSDS 2.0. The red box represents CDS, the dark blue box represents UTR, the green box represents the domain DUF573. (**B**) The conserved motif analyzed by the MEME tool. Boxes with different colors represent different motifs. (**C**,**D**) Interspecies collinearity analysis between tomato, *Arabidopsis,* and potato. Gray lines represent collinearity relationships among different genes in different species. The purple lines represent collinearity relationships among *GeBP* genes.

The examination of gene structure helps to determine the crucial position of multi-gene families in evolutionary processes. The NJ phylogenetic relationship of 34 GeBP proteins from *Arabidopsis* and tomato was constructed to analyze their gene structure and conserved motifs. The results revealed that the majority of GeBP genes contained a single exon, except SlGeBP2 and SlGeBP8, and genes sharing comparable structures were observed to be grouped closely together. Furthermore, it was noted that all genes in these two species are incorporated with a conserved DUF573 domain, suggesting its role as a core DNA-binding domain of the GeBP family (Figure 3A).

The analysis of the conserved motifs revealed that SlGeBP proteins exhibited one to seven predicted conserved motifs (Figure S2). SlGeBP7, SlGeBP8, and SlGeBP9 share similar conserved motif architectures, and SlGeBP1 and SlGeBP5 exhibit identical motif composition patterns. These members cluster within the same phylogenetic cluster, suggesting a strong evolutionary relationship. Among them, motifs 1, 2, and 3 are widely present in most of the SlGeBPs, followed progressively by motifs 4 and 5 (Figure 3B). A comparative motif architecture analysis revealed that motifs 1, 2, and 3 exhibit a significant sequence homology to the core DNA-binding domain of DUF573, with motifs 1 and 4 containing the nuclear localization signal (NLS) sequence and motifs 1, 2, and 4 containing the non-typical leucine zipper regions. These findings indicate the conserved characteristics of these motifs and their potential role in DNA recognition, dimer stabilization and nuclear trafficking.

Collinearity analysis was mainly used to reveal the homology and arrangement order of genes. The duplication of genes can significantly enrich the length and abundance of the genome, thereby promoting the evolutionary process of plants and enriching the genetic diversity of species. Therefore, to gain a deeper insight into the differentiation and developmental mechanisms of the *SlGeBP* family, both interspecies and intraspecies collinearity analysis maps were conducted. It was observed that no segmental duplication events occurred within the *GeBP* gene family in tomato, which implied that the expansion of the *SlGeBP* family was most likely not due to segmental duplication (Figure S3). Interspecies collinearity analysis showed that the highest *GeBP* homologs were presented in potato, belonging to the *Solanaceae* species, with six collinear gene pairs. This was followed by *Arabidopsis*, with three collinear gene pairs. Taken together, tomato and potato shared a stronger synteny than *Arabidopsis* (Figure 3C,D).

### 2.5. Analysis of the cis-Acting Regulatory Elements in the SlGeBP Gene Promoters

**Figure 4:**
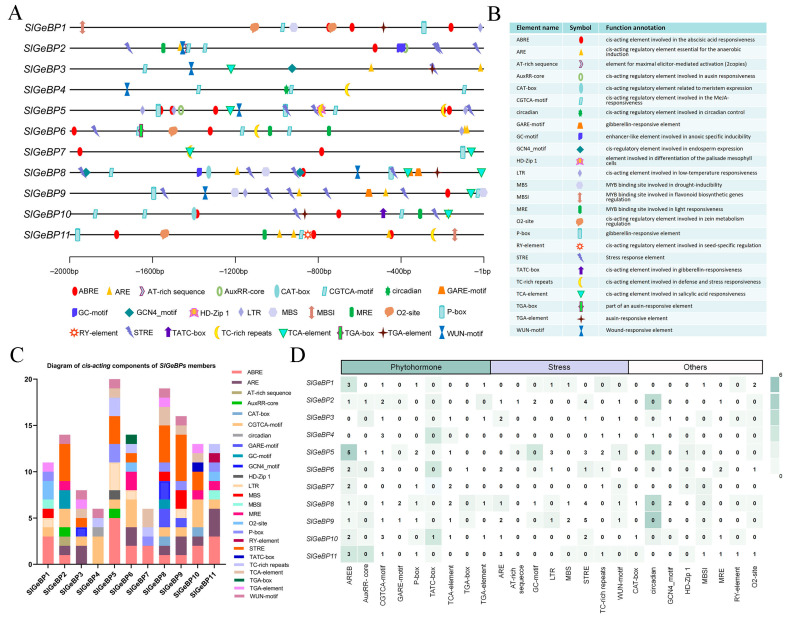
The category and position of predicted *cis-acting* elements in the *SlGeBP* gene promoters. (**A**) The position of *cis-acting* elements in the 2 kb upstream region of the 5′ UTR annotated with different symbols. (**B**) The detailed element names, symbols, and function annotations of *cis-acting* elements. (**C**) Diagram of *cis-acting* components of *SlGeBP* members. The boxes with different colors represent different *cis-acting* elements. (**D**) A total of 25 *cis-acting* elements were classified into three categories: phytohormone-responsive, stress-responsive, and plant development-related elements. The quantities of *cis-acting* elements within each gene are denoted by numbers, and deeper colors represent higher frequencies of occurrence.

A *cis-acting* element is a type of DNA sequence present in the promoter regions, which takes part in the regulation of gene expression by binding to *trans-acting* factors (such as transcription factors) to ensure that genes are expressed at the appropriate time and under suitable conditions. To predict the *cis-acting* elements present in *SlGeBP* genes, a 2 kb upstream region of coding sequences was uploaded to PlantCARE for analysis (Figure 4A). The category and location information were annotated with diverse symbols, and the element name, symbol, and the potential function annotation are listed in Figure 4B. A total of 25 *cis-acting* elements were identified and classified into three categories: hormone-responsive, stress-responsive, and plant development-related elements (Figure S4). Within this context, the largest number was present in *SlGeBP5*, with a total of 20, while the most diverse types were present in *SlGeBP8*, including 13 distinct elements (Figure 4C,D).

Among these *cis-acting* elements, the occurrence frequencies of AREB, CGTCA-motif, and STRE were relatively higher than those of others. Additionally, seven out of the eleven genes contained GARE-motif, P-box, and TATC-motif, all of which are known to be involved in GA responsiveness. Within these, some specific *cis-acting* elements were also present, such as MBSI, which may be involved in flavonoid biosynthesis gene regulation and only existed in *SlGeBP1* and *SlGeBP11*. Taken together, each *SlGeBP* gene is composed of multiple types and quantities of *cis-acting* elements, suggesting the diversity of gene function in the *SlGeBP* family.

### 2.6. Tissue-Specific Expression Profiles Analysis of SlGeBPs

**Figure 5:**
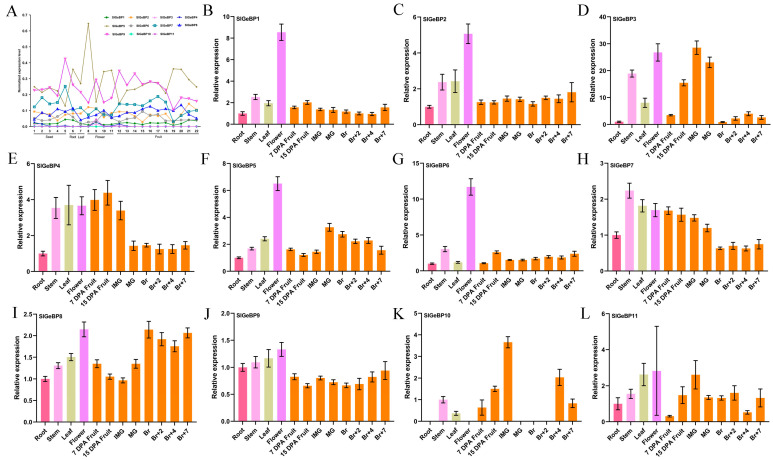
Expression profiles of *SlGeBP* genes among various tissues and different stages of tomato fruits. (**A**) Expression profiles of *SlGeBP* genes derived from TomExpress online RNA-seq data. Numerals 1–5 represent the whole seeds of immature green fruit, mature green fruit, breaker fruit, and orange and red fruit, respectively; 6 represents the whole root; 7 represents the whole leaf; 8 represents the petal of flowers; 9–11 represent the whole flower at the stage of budding, the bud at 3 mm, and anthesis; 12 represents the whole fruit of 4 DPA; 13–22 represent the flesh and peel of immature green fruit, mature green fruit, breaker fruit, orange fruit, and red fruit, respectively. (**B**) Expression profiles of *SlGeBP1* in different tomato tissues. (**C**) Expression profiles of *SlGeBP2* in different tomato tissues. (**D**) Expression profiles of *SlGeBP3* in different tomato tissues. (**E**) Expression profiles of *SlGeBP4* in different tomato tissues. (**F**) Expression profiles of *SlGeBP5* in different tomato tissues. (**G**) Expression profiles of *SlGeBP6* in different tomato tissues. (**H**) Expression profiles of *SlGeBP7* in different tomato tissues. (**I**) Expression profiles of *SlGeBP8* in different tomato tissues. (**J**) Expression profiles of *SlGeBP9* in different tomato tissues. (**K**) Expression profiles of *SlGeBP10* in different tomato tissues. (**L**) Expression profiles of *SlGeBP11* in different tomato tissues. DPA: day post anthesis. Experimental data are presented as means ± standard error from triplicate biological experiments.

Exploring their spatio-temporal expression was crucial for understanding their potential biological function; hence, the expression profiles of the *SlGeBP* genes in various tissues and during different stages of tomato fruits were investigated using the online TomExpress RNA-seq database and qRT-PCR. A total of 22 templates were picked to analyze the expression profiles via the TomExpress database (http://tomexpress.toulouse.inra.fr/query, accessed on 30 October 2024), predominantly including seeds from immature green, mature green, breaker, orange, and red fruit, roots, leaves, flowers from the petal, buds, 3 mm buds and whole flowers, and fruits during various development stages (Figure 5A, Figures S5 and S6). *SlGeBP5* and *SlGeBP9* have higher expression levels compared to others; it is particularly noteworthy that the expression level of *SlGeBP5* was higher in the flower petals and 41 DPA stage, with *SlGeBP9* having a higher expression level in seeds from red fruit. Additionally, the expression level of *SlGeBP7* was elevated to 38 DPA and then declined, suggesting that *SlGeBP7* may be able to participate in the regulation process of fruit ripening.

To better elucidate the potential function of the *SlGeBP* genes, qRT-PCR was implemented to assess the expression abundance in vegetative tissues and whole flowers in the stage of anthesis, as well as fruits during different stages (7 DPA fruit; 15 DPA fruit; immature green fruit; mature green fruit; breaker fruit; 2 days, 4 days, and 7 days after breaker fruit). Distinctly, the expression of *SlGeBPs* was observable in the majority of tomato tissues, except for *SlGeBP10*, which was only detected in specific tissues, mainly consisting of the stem, leaf, 7 DPA fruit, 15 DPA fruit, immature green fruit, and 4 days and 7 days after breaker fruit (Figure 5B–L). Among them, *SlGeBP1* to *SlGeBP6* showed a higher expression in flower organs, implying that they may play a potentially important role in flower organ development and fruit set. Moreover, the expression levels of *SlGeBP3*, *SlGeBP4*, *SlGeBP10,* and *SlGeBP11* were incrementally increased during the fruit development stage, reaching their peak at the IMG and MG stages and subsequently gradually decreasing during fruit ripening, which indicated their potential biological roles in fruit development. Interestingly, *SlGeBP3* and *SlGeBP4,* as well as *SlGeBP10* and *SlGeBP11,* exhibited a close evolutionary relationship and similar expression patterns (Figure 2), and they also share the same orthologs in *Arabidopsis* (Table 1). Taken together, these results suggested that they may play a functional redundancy or synergistic role in the regulation of fruit development.

### 2.7. Expression Profiles of SlGeBP Genes in Response to Plant Hormones

Plant hormones act as significant regulators of plants’ development, maturation, senescence, and adaptation to environmental variation through spatially and temporally specific distribution and dynamic interaction networks [28,29,30,31]. Therefore, understanding the expression profiles of *SlGeBP* genes under diverse hormones’ treatment can help us to explore the potential function of *SlGeBP*.

**Figure 6:**
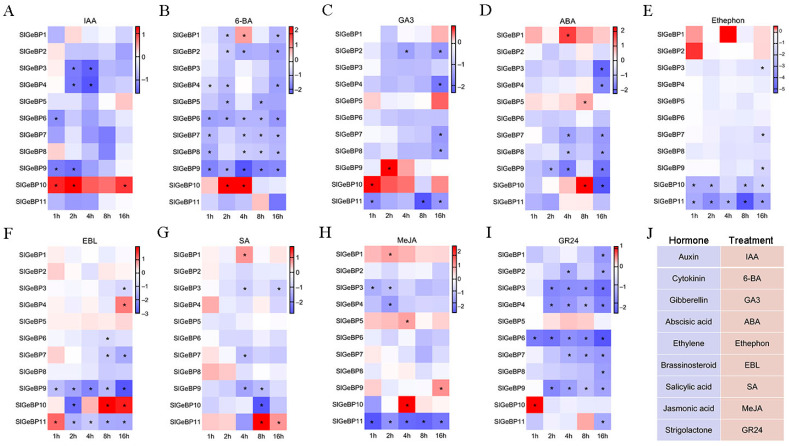
Expression profiles of *SlGeBP* genes under nine hormone treatments: (**A**) IAA; (**B**) 6-BA; (**C**) GA3; (**D**) ABA; (**E**) Ethephon; (**F**) EBL; (**G**) SA; (**H**) MeJA; (**I**) GR24. (**J**) The responsiveness of *SlGeBP* genes induced by nine different types of hormones or their analogues. After tomato seeds germinated for 12 days, samples were collected after hormone treatment at intervals of 1, 2, 4, 8, and 16 h. The expression profiles’ data were converted into log_2_ fold change and visualized by heat map. Experimental data are presented as means ± standard error from triplicate biological experiments. * refer to significant differences with *p* < 0.05 compared to the corresponding mock controls.

Under IAA treatment, only five genes significantly responded to the hormone. Among them, *SlGeBP3*, *SlGeBP4*, *SlGeBP6,* and *SlGeBP9* were significantly induced to exhibit a downregulated expression pattern, while only *SlGeBP10* was significantly upregulated, reaching its peak level at the second hour (Figure 6A). Except for *SlGeBP3* and *SlGeBP11*, all the other genes could be induced by 6-BA. However, *SlGeBP1* was upregulated at the fourth hour, and *SlGeBP10* was upregulated at the second and fourth hour, and the remaining genes showed a downregulated pattern induced by the 6-BA. Additionally, *SlGeBP1* displayed dynamic changes with the passage of time; it showed a decreased expression at the second hour, which significantly increased at the fourth hour, and then significantly declined again after induction at the sixteenth hour (Figure 6B). Under GA3 treatment for 8 h, *SlGeBP4*, *SlGeBP7,* and *SlGeBP8* showed no significant induction response, and they began to exhibit a significantly downregulated expression at the sixteenth hour. *SlGeBP9* and *SlGeBP10* exhibited a strongly upregulated expression under induction at the second hour and first hour, and neither responded to subsequent treatments (Figure 6C). For the induction of ABA, *SlGeBP1* and *SlGeBP5* showed a significant upregulation at the fourth and eighth hour, while *SlGeBP3*, *SlGeBP4*, *SlGeBP7*, *SlGeBP8,* and *SlGeBP9* exhibited a significantly downregulated expression level. *SlGeBP10* showed a significant upregulation at the eighth hour that sharply declined after the sixteenth hour of treatment (Figure 6D). Fewer genes could be induced by ethylene treatment, and all the induced genes displayed a trend of downregulated expression. Notably, *SlGeBP3*, *SlGeBP7,* and *SlGeBP9* showed a significant downregulation only after sixteen hours of induction, while *SlGeBP10* and *SlGeBP11* began to undergo downregulation as early as the first hour (Figure 6E). Under the induction of EBL, *SlGeBP3*, *SlGeBP6*, *SlGeBP7,* and *SlGeBP9* showed a downregulated expression pattern. *SlGeBP4* showed no significant changes before eight hours of treatment but demonstrated significant upregulation at the sixteenth hour. *SlGeBP10* initially exhibited a downregulated expression at the second hour, followed by a significant increase, reaching its peak at the eighth hour. *SlGeBP11* showed an upregulated expression at the first hour but subsequently displayed a declining trend, achieving the strongest decrease at the sixteenth hour (Figure 6F). *SlGeBP1* and *SlGeBP11* exhibited an upregulated induction at the fourth hour and eighth hour under SA treatment, respectively, while *SlGeBP3*, *SlGeBP7*, *SlGeBP9,* and *SlGeBP10* demonstrated a downregulated induction (Figure 6G). Under MeJA treatment, *SlGeBP1*, *SlGeBP5*, *SlGeBP9,* and *SlGeBP10* were upregulated in response to MeJA induction, while *SlGeBP3*, *SlGeBP4,* and *SlGeBP11* exhibited the opposite trend (Figure 6H). All *SlGeBP* genes, except for *SlGeBP5*, could be induced by GR24. Among them, only *SlGeBP10* exhibited upregulation by GR24 treatment, and the remaining induced genes showed a significant downregulation trend (Figure 6I). Therefore, the transcription of *SlGeBPs* can respond to multiple hormone treatments (Figure 6J), among which *SlGeBP9* and *SlGeBP10* are induced by all nine hormones, suggesting that they are likely act as specialized regulators in the hormone signaling networks.

### 2.8. Expression Profiles of SlGeBP Genes in Response to Stresses

**Figure 7:**
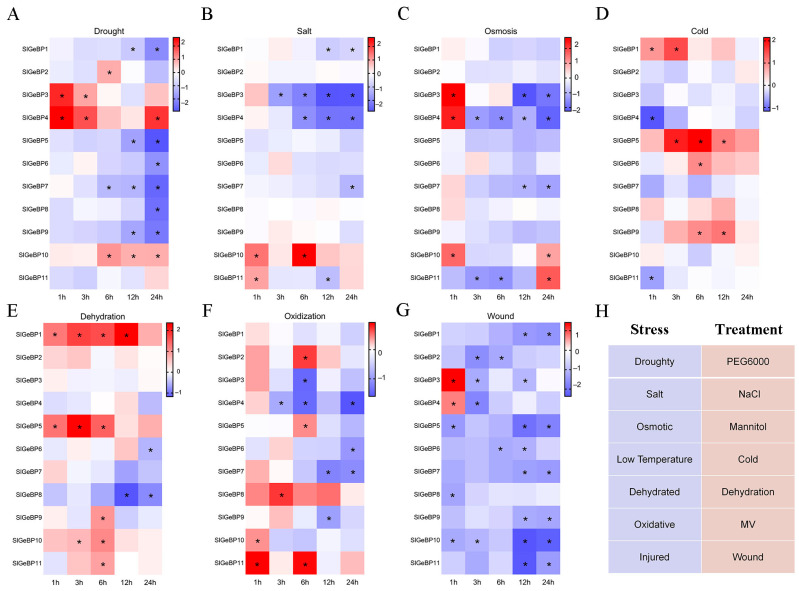
Expression profiles of *SlGeBP* genes under seven stress treatments: (**A**) Drought; (**B**) Salt; (**C**) Osmosis; (**D**) Cold; (**E**) Dehydration; (**F**) Oxidization; (**G**) Wound. (**H**) The responsiveness of *SlGeBP* genes induced by seven different stresses was analyzed by qRT-PCR, including drought (PEG6000), salt (NaCl), osmotic (mannitol), low temperature (cold), dehydrated (dehydration), oxidative (MV), and injured (wound). Samples were collected after stress treatments at intervals of 1, 3, 6, 12, and 24 h. The expression profiles’ data were converted into log_2_ fold change and visualized by heat map. Experimental data are presented as means ± standard error from triplicate biological experiments. * refer to significant differences with *p* < 0.05 compared to the corresponding mock controls.

In order to further elucidate the responsiveness of *SlGeBP* to abiotic stresses, we analyzed the transcript profiles of *SlGeBP* genes under PEG6000 (drought) (Figure 7A), NaCl (salt) (Figure 7B), mannitol (osmotic) (Figure 7C), cold (low temperature) (Figure 7D), dehydration (dehydrated) (Figure 7E), MV (oxidative) (Figure 7F), and wound (injured) (Figure 7G) stresses using qRT-PCR. Overall, *SlGeBP* genes could be influenced by multiple stresses and exhibited a varied expression pattern in response to those seven stresses (Figure 7H). All *SlGeBP* genes demonstrated sensitivity to wound stress, with *SlGeBP3* and *SlGeBP4* being upregulated at the first hour, and all 11 genes showed a significantly decreased expression during the subsequent treatment. Among the 11 *SlGeBP* genes, 10 exhibited sensitivity to both drought and oxidative stress. *SlGeBP3* and *SlGeBP4* showed a higher transcriptional upregulation level under drought stress, and *SlGeBP8* and *SlGeBP11* had the highest transcript levels under oxidative stress (Figure 7A,F). For salt treatment, the expression levels of five genes were significantly decreased, with only *SlGeBP10* reaching the peak of upregulation induction after 6 h of treatment (Figure 7B). During osmotic stress, *SlGeBP3* and *SlGeBP4* were transiently upregulated at the first hour, with a rapid decline during subsequent treatment. *SlGeBP11* initially decreased at the third and sixth hours, followed by a significantly upregulated induction at the twenty-fourth hour of treatment (Figure 7C). The expression levels of *SlGeBP1*, *SlGeBP5,* and *SlGeBP9* were upregulated under both cold and dehydration stress, while *SlGeBP6* and *SlGeBP11* displayed antagonistic expression profiles (Figure 7D,E). The comprehensive analysis of these findings suggest that *SlGeBP* genes are involved in stress response, implying their potential role in plant environmental adaptation.

### 2.9. Prediction of the Protein–Protein Interaction with SlGeBP Proteins

**Figure 8:**
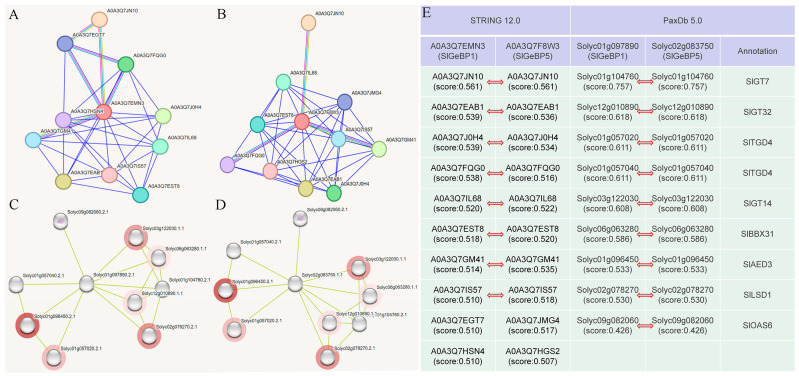
Protein–protein interaction network of SlGeBP1 and SlGeBP5 in tomato. (**A**,**B**) The predicted PPI network of SlGeBP1 and SlGeBP5 in the STRING database. (**C**,**D**) The predicted PPI network of SlGeBP1 and SlGeBP5 in the PaxDb database. (**E**) The detailed interacting proteins are listed in the table.

To further clarify the biological function of the *SlGeBP* genes, a protein–protein interaction (PPI) network analysis was conducted to predict their interacting proteins (Table S3). Through the integration of interaction predictions from the STRING 12.0 and PaxDb 5.0 databases, this study identified candidate protein–protein interaction partners for SlGeBP1 and SlGeBP5, laying a foundation for further mechanistic exploration (Figure 8). The prediction results indicated that the interacting partners of SlGeBP1 and SlGeBP5 include three genes of the trihelix family of transcription factors, namely SlGT7, SlGT14, and SlGT32 [32]. The *SlGT* gene family contains a conserved trihelix structure (helix–loop–helix–loop–helix); these genes have been reported to participate in light signal transduction and the response to various stresses, and they contribute to biological functions such as organ morphogenesis and trichome development [33]. Prediction interaction analysis showed that two *TGD* family genes, homologous to At3g06960 in *Arabidopsis*, shared an interaction with SlGeBP1 and SlGeBP5. The ABC transporter system trigalactosyldiacylglycerol TGD4 was able to transport polar lipids from the endoplasmic reticulum to chloroplasts and traffic phosphatidic acid across the outer envelope membrane [34,35]. In addition to the five genes with a prediction score greater than 0.6, four additional genes, BBX31, AED3, LSD1, and OAS6, were also predicted to be interaction partners of SlGeBP1 and SlGeBP5. Among those, BBX31 [36] and OAS6 [37] have been reported to participate in temperature stress and drought stress, respectively, while AED3 [38] and LSD1 [39,40,41] are involved in programmed cell death (PCD), thereby contributing to pathogen defense.

### 2.10. The Interaction Between and Subcellular Localization of SlGeBP1 and SlGeBP5

Based on them being on the same branch in their evolutionary relationship and the similar expression patterns between SlGeBP1 and SlGeBP5 (Figure S7), we hypothesized that they might interact with each other or exhibit a functional redundancy in the performance of biological functions.

**Figure 9:**
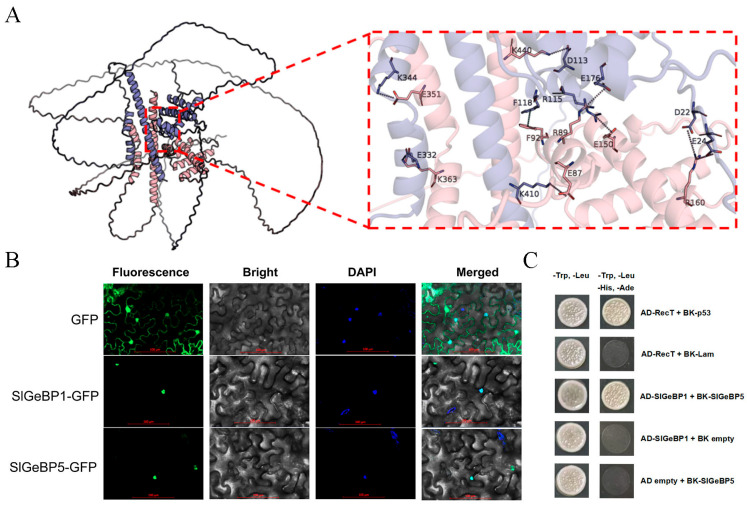
The interaction and subcellular localization of SlGeBP1 and SlGeBP5. (**A**) A 3D model of the molecular docking of SlGeBP1 and SlGeBP5. The pink protein structure represents SlGeBP1, and the purple protein structure represents SlGeBP5. The zoom-in areas on the side of the protein structure indicate the binding sites in the docking proteins. The protein complex model was predicted by AlphaFold3, and PyMol 2.6.0a0 (Schrödinger, LLC, New York, NY, USA) was used to render the binding site. (**B**) Subcellular localization of SlGeBP1 and SlGeBP5 in *N. benthamiana* leaves. DAPI staining as a nuclear localization marker to delineate the nucleus boundaries. Scale bars: 100 µm. (**C**) Yeast two-hybrid assay confirmed the interaction between SlGeBP1 and SlGeBP5. The pGADT7-RecT and pGBKT7-p53 act as a positive control, and the pGADT7-RecT and pGBKT7-Lam act as a negative control.

To predict whether they can interact with each other, PEPPI (https://zhanggroup.org/PEPPI/, accessed on 2 March 2025) and MEGADOCK 4.0 (Tokyo Institute of Technology, Tokyo, Japan) were used to estimate the probability of SlGeBP1 and SlGeBP5 interactions. The prediction results suggest a high probability of interaction between them (Figure S8). Furthermore, AlphaFold3 was used to predict the three-dimensional structures of the proteins and the interaction sites between the proteins, and rendering and visualization was carried out using PyMol (Figure 9A). As we can see from the prediction results, nine binding sites were present between SlGeBP1 and SlGeBP5. Those binding sites were E351 and K344, K363 and E332, K440 and D113, F92 and F118, E87 and K410, R160 and D22, R160 and E24, R89 and E176, and E150 and R115. Subcellular localization analysis revealed that both SlGeBP1 and SlGeBP5 were specially localized in the nucleus (Figure 9B). To further validate the previous predictive analysis, yeast two-hybrid assays were conducted to verify the interaction between SlGeBP1 and SlGeBP5 (Figure 9C). The findings indicated that SlGeBP1 and SlGeBP5 can interact with each other, suggesting that they may jointly form a functional dimer to perform biological functions.

## 3. Discussion

Within plants, there exists a comprehensive and systematic regulatory network that manipulates numerous physiological and biochemical processes, among which transcription factors play a pivotal role throughout the life cycle of plants [24]. Therefore, exploring the structure and function of transcription factors at a genome-wide level, as well as their response to hormones and stresses, is of great importance for elucidating their biological function. The GeBP transcription factors are unique, existing in plants and taking part in a range of biological processes, including trichome development, plant development and senescence, and resistance to stress and pathogen injection in *Arabidopsis* [42,43,44,45]. Since it was identified in *Arabidopsis*, a comprehensive genome-wide identification of the *GeBP* family has been carried out within various species, such as dicotyledonous plants, including *B.rapa*, *G.max*, upland cotton [46], and *M.domestica*, as well as monocotyledonous, plants including *O.sativa*, *Triticum aestivum* L., and *Z. mays* [11]. To date, only the *GeBP* genes in *Arabidopsis* have been extensively and deeply studied, while the available data on the exploration of their function and molecular mechanism in other species are still quite limited, especially in tomato.

During this research, 11 *SlGeBP* genes were identified using bioinformatic techniques. The majority of genes in *Arabidopsis* and tomato were found in distinct subfamilies, with both existing in subfamily A and subfamily B, suggesting that they may share similar functions. Interestingly, all members in subfamily C were from the monocotyledonous plants, and interspecies collinearity analysis data also demonstrate a stronger synteny with dicotyledonous plants compared with monocotyledonous plants. The *CsGeBP* genes clustered together in Group I and Group II, while other *AtGeBP* genes clustered separately in Group III, and *OsGeBP* genes clustered separately in Group VI, indicating differential gene loss during species divergence [6]. The *GeBP* gene family in apples underwent an independent evolution during the differentiation of apple species, highlighting their unique evolutionary trajectory [9]. Evolutionary analysis revealed that the expansion mechanisms of the *GeBP* gene family diverged significantly across nine Gramineae crops, reflecting their individual responses to selective pressures and ecological adaptations [11]. Taken together, these findings highlight the evolutionary expansion and diversification of *GeBP* family members.

Gene structure analysis revealed that the SlGeBP proteins contain a conserved DNA-binding domain and an atypical leucine zipper. The presence of these two domains confers upon GeBP the dual capacity to precisely recognize downstream target genes and mediate protein dimerization, with a functional equivalence to the standard leucine zippers [7,14]. Studies have demonstrated that NbGeBP interacts with pathogenic factors encoded by geminiviruses, contributing to host defense mechanisms against viral infection. Notably, NbGeBP can form homodimers, and the interaction with pathogenic factors is independent of both the conserved DNA-binding domain and an atypical leucine zipper. This suggest that GeBP and its interacting partners may assemble into diverse complex regulatory modules to execute their function [44]. Interaction prediction analysis revealed over 80% of the predicted interacting proteins between SlGeBP1 and SlGeBP5. Concurrently, under hormone treatments and abiotic stresses, *SlGeBP1* and *SlGeBP5* exhibited tightly synchronized expression dynamics, suggesting coordinated roles in environmental signal integration. Based on the integrated bioinformatic and expression profile evidence described above, we propose the hypothesis that there is a functional interaction between SlGeBP1 and SlGeBP5. The interaction between GeBP1 and SlGeBP5 was preliminarily verified through yeast two-hybrid experiments. However, further validation through experimental approaches is required to confirm their interaction. Subsequent research will focus on constructing SlGeBP1 and SlGeBP5 mutants, as well as SlGeBP1/SlGeBP5 double mutants, to analyze their specific biological function and determine whether the dimer they form exhibits functional redundancy or synergy.

Current research on the functional characterization of *SlGeBP* family members remains limited. We thus preliminarily inferred their potential biological functions through integrated bioinformatics analyses, including tissue-specific expression profiling, the identification of *cis-acting* elements in promoter regions, and the prediction of interacting proteins. *SlGeBP2* exhibited an elevated expression during flowering, coinciding with the high expression of its predicted interactor NOP58 (A0A3Q7J2J4) in anthers [47]. This suggested that *SlGeBP2* may function in flowering or fruit set. Furthermore, *SlGeBP2* expression was higher during fruit ripening than fruit development. Another predicted interactor was NOP56 (A0A3Q7F8Q2, a candidate ripening regulator) [48], supporting the potential role of *SlGeBP2* in tomato fruit ripening. Additionally, SlGeBP2 was predicted to interact with CIP3/FKBP12, which mediates rapamycin-TOR signaling—a central kinase regulating tomato metabolic processes, including photosynthesis, cell wall restructuring, and senescence [49]. The promoter region of *SlGeBP3* contains both a CGTCA-motif and TCA-element, *cis*-regulatory elements associated with MeJA and salicylic acid responsiveness. Consistent with this, *SlGeBP3* exhibited a downregulated expression under MeJA and SA treatment. SlWRKY70 (K4BYC4_SOLLC), which was predicted to interact with SlGeBP3, was likewise induced by SA and participates in tomato immune responses against aphids and root-knot nematodes [50]. Collectively, these findings suggest *SlGeBP3* may regulate pathogen defense mechanisms. Notably, *SlGeBP3* expression progressively increased during fruit development, peaking at the IMG stage before declining through the ripening—implying additional roles in developmental processes. The promoter region of *SlGeBP4* harbors three CGTCA-motifs and one TC-rich repeat, suggesting it may be involved in MeJA-mediated stress responses. Given its suppressed expression under both MeJA and oxidative treatments, we propose that *SlGeBP4* functions as a negative regulator of stress and pathogen resistance. Interestingly, a prior protein interaction prediction and preliminary yeast two-hybrid assay confirmed that SlGeBP1 and SlGeBP5 could form a function dimer. Additionally, interaction predictions also indicated a potential interaction between SlGeBP6 (A0A3Q7HF63) and SlGeBP8 (A0A3Q7I8D9), and this result requires further experimental validation. Concurrently, SlGeBP6 may interact with A0A3Q7EKC9 (Chloroplast Vesiculation, CV)—a protein whose CRISPR/Cas9 knockout enhances stress tolerance across vegetative and reproductive stages, consequently improving fruit yield and quality [51]. This functional association gains significance as *SlGeBP6* expression progressively increases from immature green (IMG) to ripening stages, suggesting synergistic roles in the regulation of ripening and development of flavor. *SlGeBP7* expression progressively decreases throughout fruits’ development and ripening. Its interaction partner AMP (A0A3Q7HYM8) elevates inosine monophosphate and guanosine monophosphate levels—key umami-enhancing nucleotides in fruits [52]. This inverse expression pattern suggests *SlGeBP7* may negatively regulate pathways for the accumulation of flavor compounds, potentially contributing to the improvement of fruit quality. The presence of a low-temperature-responsive element in *SlGeBP9* and its upregulation under cold stress implicate it in regulating the cold stress response. *SlGeBP10* exhibited a tissue-specific expression in vegetative organs (stems and leaves) and during fruit developmental stages—including 7 DPA, 15 DPA, immature green (IMG), and 4 days and 7 days after breaker fruit—indicating its stage-specific functional importance. The coexistence of ABA/IAA-responsive *cis*-elements (AREB, TGA-box) with a significant downregulated expression under both IAA and ABA treatments suggest that it may mediate hormone-regulated biological processes. Among *SlGeBP* family members, only *SlGeBP1* and *SlGeBP11* contain the MBSI *cis*-element—a MYB-binding site regulating flavonoid biosynthetic genes—suggesting their potential role in aromatic compounds’ synthesis and metabolism. Furthermore, *SlGeBP11* harbors multiple plant development-associated *cis*-regulatory elements, such as seed-specific regulatory element and zein metabolism regulation element, implying additional roles in seed development or germination processes.

**Figure 10:**
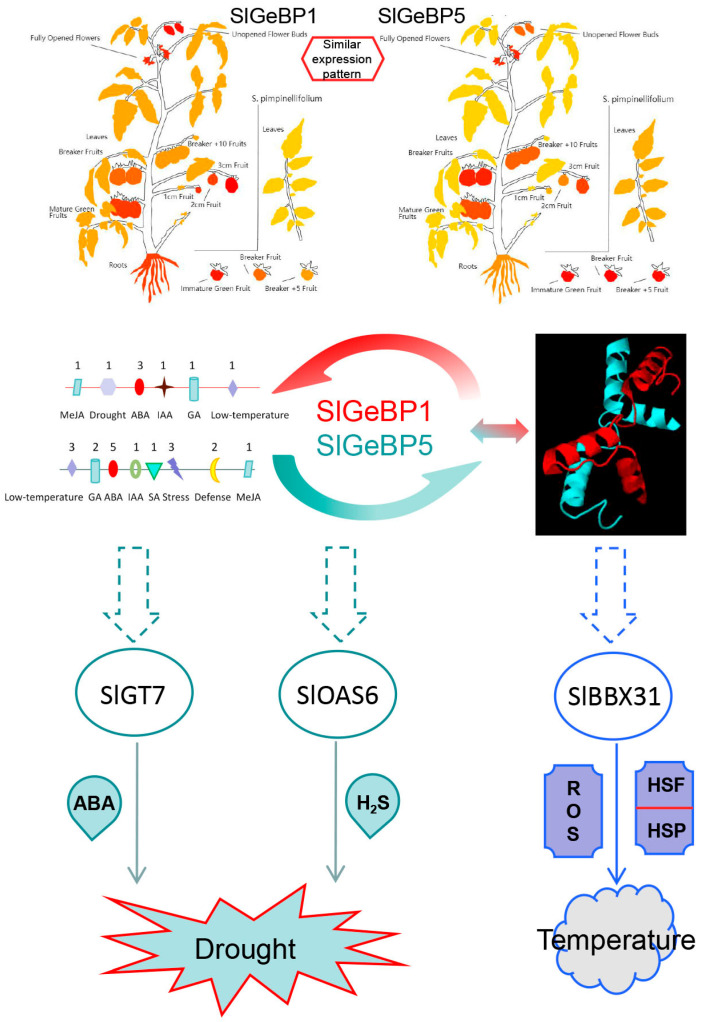
The hypothesized regulatory mechanisms of SlGeBP1 and SlGeBP5 under drought and temperature.

To avoid damage from drought, a higher ABA concentration in plants exists to reduce transpiration by closing stomata to prevent the loss of water and synthesis-relevant substances to maintain the balance of osmotic pressure [53,54,55]. *SlGT7* and *SlOAS6* have been reported to function in the ABA-dependent drought response pathway. SlGT7 exhibited a high expression level in leaves, as well as during the Br+4 and Br+7 stages. It was reported that water could suppress the expression of *SlGT7* in tomato seedling leaves, indicating that *SlGT7* might respond to water stress by participating in the ABA pathway [33]. SlOAS6, O-acetylserine (mercaptan) lyases, was found to be involved in the production of H_2_S. H_2_S facilitates stomatal closure by inhibiting the inward flow of K^+^ channels and widely participates in ABA signaling in plants, thereby contributing to various physiological functions [36]. Functional prediction analysis showed that SlGT7, SlOAS6, and SlBBX31 might interact with SlGeBP1 and SlGeBP5 (Figure 10). SlBBX31 could participate in the physiological processes of photosynthesis, flowering, and fruit ripening in tomato. It also plays a vital role in the regulation of temperature stress by enhancing cell membrane stability, increasing the activity of antioxidant enzymes, and scavenging reactive oxygen species [36]. Although this work provides an initial bioinformatic exploration of *SlGeBP* family functions and mechanisms, further validation through the tomato mutants lines, physiological assays, and more molecular biology evidence is essential.

*GeBP* have also been reported to be involved in plant pathogen resistance. Acting as a susceptibility factor, the knockout of *AtGeBP* significantly reduced the accumulation of viral DNA [44]. The *GPL2*-Ox line displayed a strong resistance to *Pst* DC3000 in *Arabidopsis* [43]. Recent studies have identified that SlGeBP6, a member of the tomato GeBP family, could interact with TbCSB and TYLCCNB βC1 to participate in virus infection [44]. Phylogenetic tree analysis revealed that SlGeBP1, GeBP5, and SlGeBP6 cluster within the same clade, suggesting they might share similar structures and may perform analogous biological functions. Through interaction prediction analysis, it has been observed that SlGeBP1 and SlGeBP5 might interact with AED3 and LSD1, which are linked to disease resistance. AED3, aspartyl protease 3, was highly expressed in leaves and NPCD cells in lace plants, and it is involved in programmed cell death processes to trigger autophagy and pathogen defense responses [38]. LSD1, lesion simulating disease 1, acts as a negative regulator of cell death and plays a crucial role in hypersensitive responses and programmed cell death, thereby being able to protect the plant cells against the oxidative stress [39,40,41]. Future studies will also focus on delineating the precise roles of GeBP proteins in phytohormone signaling, stress tolerance, and pathogen resistance, leveraging CRISPR-Cas9 mediated gene editing, transcriptomic profiling, and protein interaction networks to dissect their dual functionality in defense signaling and susceptibility mechanisms.

## 4. Materials and Methods

### 4.1. Plant Materials and Growth Conditions

Tomato (*Solanum lycopersicum* L.) cv. Micro-Tom was used in this study and cultured under the following conditions: a 16 h light/8 h dark cycle, temperatures of 25 °C during the day and 18 °C at night, and a relative humidity of 60%. To investigate the tissue-specific expression patterns of *SlGeBP* family genes, samples were collected from roots, stems, leaves, flowers, and fruits during different stages, including 7 DPA fruit, 15 DPA fruit, immature green fruit, mature green fruit, breaker fruit, and 2 days, 4 days, and 7 days after breaker fruit. Vegetative tissues were collected from at least ten individual one-month-old tomato seedlings. Whole flowers in the stage of anthesis were collected from at least 30 individual healthy plants. The different stages of fruits were collected from at least 10 individual healthy plants. All samples were immediately frozen with liquid nitrogen and stored at −80 °C for further analysis.

### 4.2. Identification of SlGeBP in Tomato

To identify SlGeBP in tomato, the domain of PF04504 (downloaded from Pfam) (https://www.ebi.ac.uk/interpro/, accessed on 12 September 2024), was used to construct a hidden Markov model (HHM). The HMMER website (http://hmmer.org/, accessed on 13 September 2024) was used to search the potential tomato GeBP proteins. The candidate proteins were further verified using Pfam (accessed on 15 September 2024) and NCBI CD-Search (https://www.ncbi.nlm.nih.gov/Structure/cdd/wrpsb.cgi/, accessed on 15 September 2024). Taken together, all candidate proteins that contained the PF04504 domain were regarded as members of the SlGeBP family. The genome data were downloaded from the ensemblPlants database (https://plants.ensembl.org/, accessed on 30 September 2024), including tomato (*Solanum lycopersicum* L.), *Arabidopsis* (*Arabidopsis thaliana* L.), soybean (*Glycine max* L.), pepper (*Capsicum annuum* L.), maize (*Zea mays* L.), and rice (*Oryza sativa* L.). The protein sequences and gene IDs used in this study are listed in Table S4.

### 4.3. Physicochemical Characterization and Multiple Sequence Alignment of SlGeBP Proteins

The genomic locus and exon numbers were obtained from the Sol Genomics Network (https://solgenomics.net/, accessed on 20 September 2024). The physical and chemical characteristics of the SlGeBP proteins were predicted using Expasy (https://web.expasy.org/protparam/, accessed on 22 September 2024). Based on the annotation of the genome information, MG2C V2.1 tools (http://mg2c.iask.in/mg2c_v2.1/, accessed on 25 September 2024) were used to map the location and distribution of *SlGeBP* genes on the chromosomes. The MUSCLE program in MEGA X software v10.1.8 (Temple University, Philadelphia, PA, USA) was used for the multiple sequence alignment of AtGeBP and SlGeBP proteins, and the picture was rendered using ESPript 3.0 (https://espript.ibcp.fr/ESPript/ESPript/index.php, accessed on 12 October 2024).

### 4.4. Three-Dimensional Structure, Secondary Structure, and Subcellular Localization Prediction

The AlphaFold protein structure database (https://alphafold.ebi.ac.uk/, accessed on 14 October 2024) was used to build the three-dimensional structure of SlGeBP proteins. SOPMA (https://npsa-prabi.ibcp.fr/cgi-bin/npsa_automat.pl?page=/NPSA/npsa_seccons. html, accessed on 14 October 2024) was employed to predict the secondary structure of SlGeBP members. To predict the subcellular localization of SlGeBP proteins, four prediction tools were utilized for analysis, including Wolf PSort (https://www.genscript.com/wolf-psort.html, accessed on 15 October 2024), BaCelLo (https://busca.biocomp.unibo.it/bacello, accessed on 15 October 2024), CELLO (http://cello.life.nctu.edu.tw/, accessed on 15 October 2024), and ePlant (https://bar.utoronto.ca/eplant_tomato/, accessed on 15 October 2024). The detailed 3D model and secondary structure are listed in Table S1 and the predicted subcellular localization of SlGeBP proteins are listed in Table S2.

### 4.5. Phylogenetic Analysis

To explore the evolutionary relationships among the GeBP family, 97 full-length GeBP proteins were obtained for 6 species—including *Arabidopsis*, soybean, pepper, maize, rice and tomato—and the neighbor joining (NJ) method in MEGA X via a 1000 bootstrap replication and iTOL v7 (https://itol.embl.de/, accessed on 17 October 2024) was implemented for the annotation, management, and formation of the phylogenetic tree.

### 4.6. Gene Structure, Conserved Motifs, and Collinearity Analysis of SlGeBP Proteins

The quantities and locations of introns, exons, and functional domains were visualized using GSDS 2.0 (https://gsds.gao-lab.org/, accessed on 18 October 2024). The conserved motif analysis diagram of 10 conserved motifs was constructed using MEME 5.5.7 (https://meme-suite.org/meme/, accessed on 19 October 2024). The conserved motifs were visualized using WebLogo (https://weblogo.berkeley.edu/logo.cgi, accessed on 19 October 2024) and confirmed with Pfam. The Advanced Circos program in TBtools v2.154 (South China Agricultural University, Guangdong, China) was used for intraspecies collinearity analysis. One Step MCScanX and Dual Systeny Plot in TBtools were used for interspecies collinearity analysis. The detailed information of the location and annotation of function domains is listed in Table S5.

### 4.7. cis-Acting Element Analysis

To predict the *cis-acting* elements in the *SlGeBP* genes, a 2 kb upstream region of *SlGeBP* coding sequences was uploaded into the PlantCARE database (http://bioinformatics.psb.ugent.be/webtools/plantcare/html/, accessed on 25 October 2024). The detailed information on *cis-acting* elements is listed in Table S6.

### 4.8. Hormone and Stress Treatments

Following Su et al. [56], the protocols for hormone and stress treatments were applied. With regard to hormone treatments, after seedlings’ germination on a solid medium for 12 days, the seedlings were then transplanted into a liquid medium supplemented with 20 µM IAA, 100 µM ABA, 0.5 µM EBL, 10 µM 6-BA, 20 µM ethephon, 20 µM GA3, 5 µM GR24, 50 µM MeJA, and 20 µM SA. Incubation was carried out at 25 °C under dark conditions. Samples were collected after hormone treatment at intervals of 1, 2, 4, 8, and 16 h.

For the stress treatments, tomato seedlings cultivated for 30 days were immersed in solutions supplemented with 100 mM mannitol, 200 mM NaCl, 20% (m/v) PEG6000, and 150 µM methyl viologen, matched with osmotic, salt, drought, and oxidative stress treatments, respectively, and then cultured under a standard growth environment. For the cold treatment, the tomato seedlings were transferred into the refrigerated illuminating incubator at 4 ± 1 °C. For the dehydrated stress treatment, tomato plants were extracted from the soil and rinsed with water and then placed on absorbent paper until dried, followed by collection of the leaves. For the injured stress treatment, tomato leaves were punctured by tweezers. Samples were collected after stress treatment at intervals of 1, 3, 6, 12, and 24 h. Three individual tomato leaves at the same position were harvested as a single sample, and the experiments were performed with three independent biological replicates.

### 4.9. RNA Extraction and qRT-PCR

Total RNA was isolated using a TIANGEN RNAprep Kit (TIANGEN, Beijing, China) in accordance with the recommended protocol. Subsequently, the synthesis of the first cDNA strand was carried out with the TAKARA PrimeScript™ RT Reagent Kit, which includes a gDNA Eraser. qRT-PCR was conducted by employing TB Green^TM^ Primix Ex TaqTM II (TaKaRa, Tokyo, Japan) on the Bio-Rad CFX96 Detection System (BIO-RAD, Hercules, CA, USA). The housekeeping gene SlUBI and the 2^−ΔΔCt^ method were used as internal references, and a visual map was generated with GraphPad Prism 9.0 (GraphPad Software Inc, San Diego, CA, USA).

### 4.10. Predicting the Interactors with SlGeBP Proteins

STRING v12.0 (https://cn.string-db.org/, accessed on 28 February 2025) and PaxDb v5.0 (https://pax-db.org/, accessed on 1 March 2025) were used to predict the interactors with SlGeBP proteins and create the protein interaction network. The minimum required interaction score was a medium confidence at 0.4, and the max number of interactors was no more than 10 interactors.

The PEPPI online server (https://zhanggroup.org/PEPPI/, accessed on 2 March 2025) and MEGADOCK 4.0 (Tokyo Institute of Technology, Tokyo, Japan) were used to predict the interaction between SlGeBP1 and SlGeBP5. The protein complex model was predicted using AlphaFold3 (https://golgi.sandbox.google.com/, accessed on 2 March 2025), and PyMol 2.6.0a0 (Schrödinger, LLC, New York, NY, USA) was used to render the binding sites.

### 4.11. Subcellular Localization of SlGeBP1 and SlGeBP5

A transient infiltration assay was used to investigate the subcellular localization of SlGeBP1 and SlGeBP5. The coding sequences of *SlGeBP1* and *SlGeBP5* without a stop codon were cloned and fused into the pK7WGF2 with a ClonExpress II One Step Cloning Kit (Vazyme, Nanjing, China).

The recombined vector was transformed into *Agrobacterium tumefaciens* and suspensions at an OD600 value of 0.8, then injected into *Nicotiana benthamiana* L. leaves, as previously described in Cao et al. [57]. After infiltration, the *N. benthamiana* plants were placed in complete darkness for 24 h and transferred to standard growth conditions and incubated for two or three days. 4′,6-diamidino-2-phenylindole (DAPI) was employed as a nuclear localization marker to delineate the nucleus boundaries. Green fluorescent protein (GFP) fluorescence and DAPI were excited at 488 nm and 408 nm and visualized using a confocal fluorescence microscope. The primers utilized in this study are documented in Table S7.

### 4.12. Yeast Two-Hybrid Assay (Y2H)

For the Y2H assay, the coding sequences of *SlGeBP1* were ligated into pGADT7 plasmid, acting as prey, and the coding sequences of *SlGeBP5* were ligated into pGBKT7 plasmid, acting as bait. Then, the prey and bait plasmids were transformed into Y2H Gold yeast strains. The pGADT7-RecT and pGBKT7-p53 plasmids functioned as a positive control, and the pGADT7-RecT and pGBKT7-Lam plasmids functioned as a negative control. The transformed yeast was grown on SD medium lacking Leu and Trp (SD/-Leu -Trp) for 2 days, and then the yeast colonies were transferred to an SD/-Leu -Trp -His -Ade medium containing 500 ng/mL AbA to analyze their interaction.

## 5. Conclusions

This study identified 11 *SlGeBP* transcription factor family members and revealed insights into their structural characteristics, phylogenetic relationships, and potential regulatory roles. Expression profiling under various hormone and stress treatments demonstrated the responsiveness of *SlGeBP* genes to diverse stimuli, suggesting their involvement in phytohormone signaling and stress tolerance. Subcellular localization analysis indicated that SlGeBP1 and SlGeBP5 are localized in the nucleus, and their interaction was confirmed via a yeast two-hybrid assay. These findings provide a foundation for understanding the potential functions of the *SlGeBP* family and offer valuable genetic resources for future research aimed at improving stress resistance and hormone responses in tomato.

## Figures and Tables

**Table 1:** Detailed information of SlGeBP family genes and encoded proteins identified in tomato.

Gene Name	Gene Accession	Genomic Locus	Exon	AA	MW(Da)	pI	Strand	A.I	GRAVY	Best Hit in Arabidopsis
*SlGeBP1*	Solyc01g097890	ch01:88581415..88583129	1	454	48,609.88	5.83	-	73.60	−0.371	AT3G04930.2
*SlGeBP2*	Solyc02g005290	ch02:7633701..7645348	8	519	57,645.42	9.45	-	85.08	−0.491	AT1G61730.1
*SlGeBP3*	Solyc02g014740	ch02:18023947..18024618	1	224	26,187.87	9.24	+	69.46	−1.073	AT4G00390.1
*SlGeBP4*	Solyc02g014750	ch02:18044460..18044933	1	158	18,184.97	8.66	+	71.97	−0.617	AT4G00390.1
*SlGeBP5*	Solyc02g083750	ch02:47039175..47040441	1	422	46,853.96	4.71	-	71.28	−0.687	AT5G28040.1
*SlGeBP6*	Solyc05g051330	ch05:61607815..61608822	1	336	37,761.07	5.00	-	72.96	−0.692	AT5G28040.1
*SlGeBP7*	Solyc07g052760	ch07:61209111..61210259	1	383	42,923.35	6.42	+	59.48	−1.000	AT4G00390.1
*SlGeBP8*	Solyc07g052830	ch07:61287331..61288758	2	321	35,723.94	5.12	+	65.88	−0.909	AT4G00390.1
*SlGeBP9*	Solyc07g052900	ch07:61314239..61315432	1	398	44,075.41	5.80	+	64.13	−0.972	AT4G00390.1
*SlGeBP10*	Solyc07g063840	ch07:66179566..66180037	1	157	18,606.58	5.20	+	58.14	−1.295	AT2G25650.1
*SlGeBP11*	Solyc07g064000	ch07:66308723..66309386	1	221	25,221.15	4.26	-	63.82	−1.167	AT2G25650.1

AA: amino acid; MW: molecular weight; pI: isoelectric point; A.I: aliphatic index; GRAVY: grand average of hydropathicity.

## Data Availability

Data is contained within the article or Supplementary Materials.

## Supplementary Materials

The following supporting information can be downloaded at: https://www.mdpi.com/article/10.3390/ijms26136008/s1.

## Abbreviations

The following abbreviations are used in this manuscript:

bZIP	Basic region leucine zipper
CD-Search	Conserved domain search
DPA	Day post anthesis
DUF573	Domain of Unknown Function 573
GeBP	The GLABROUS1 enhancer-binding protein
GPL	GeBP-like
HHM	Hidden Markov model
MV	Methyl viologen
NCBI	National Center for Biotechnology Information
NJ	Neighbor joining method
NLS	Nuclear localization signal
PaxDb	Protein Abundance Database
PEPPI	Pipeline for the Extraction of Predicted Protein-protein Interactions
pLDDT	Per-residue model confidence score
PPI	protein-protein interaction
qRT-PCR	Quantitative real-time polymerase chain reaction
RRs	response regulators
TAIR	The Arabidopsis Information Resource
TF	Transcription factor

## References

[1] Wang X., Shen C., Meng P., Tan G., Lv L. (2021). Analysis and review of trichomes in plants. BMC Plant Biol..

[2] Schuurink R., Tissier A. (2020). Glandular trichomes: Micro-organs with model status?. New Phytol..

[3] Huchelmann A., Boutry M., Hachez C. (2017). Plant glandular trichomes: Natural cell factories of high biotechnological interest. Plant Physiol..

[4] Kortbeek R., Galland M., Muras A., Therezan R., Maia S., Haring M., Schuurink R., Bleeker P. (2023). Genetic and physiological requirements for high-level sesquiterpene-production in tomato glandular trichomes. Front. Plant Sci..

[5] Berhin A., Nawrath C., Hachez C. (2022). Subtle interplay between trichome development and cuticle formation in plants. New Phytol..

[6] Chalvin C., Drevensek S., Dron M., Bendahmane A., Boualen A. (2020). Genetic control of glandular trichome development. Trends Plant Sci..

[7] Chevalier F., Perazza D., Laporte F., Le Hénanff G., Hornitschek P., Bonneville J., Herzog M., Vachon G. (2008). GeBP and GeBP-Like proteins are noncanonical leucine-zipper transcription factors that regulate Cytokinin response in *Arabidopsis*. Plant Physiol..

[8] Khare D., Mitsuda N., Lee S., Song W., Hwang D., Ohme-Takagi M., Martinoia E., Lee Y., Hwang J. (2017). Root avoidance of toxic metals requires the GeBP-LIKE 4 transcription factor in *Arabidopsis thaliana*. New Phytol..

[9] Liu R., Li H., Qiao Z., Liu H., Zhao L., Wang X., Zhang Z., Zhang S., Song L., You C. (2023). Genome-wide analysis of *MdGeBP* family and functional identification of *MdGeBP3* in *Malus domestica*. Environ. Exp. Bot..

[10] Liu S., Liu Y., Liu C., Zhang F., Wei J., Li B. (2022). Genome-wide characterization and expression analysis of GeBP family genes in soybean. Plants.

[11] Huang J., Zhang Q., He Y., Liu W., Xu Y., Liu K., Xian F., Li J., Hu J. (2021). Genome-wide identification, expansion mechanism and expression profiling analysis of *GLABROUS1 enhancer-binding protein (GeBP)* gene family in Gramineae crops. Int. J. Mol. Sci..

[12] Wang R., Wu X., Wang Z., Zhang X., Chen L., Duan Q., Huang J. (2023). Genome-wide identification and expression analysis of *BrGeBP* genes reveal their potential roles in cold and drought stress tolerance in *Brassica rapa*. Int. J. Mol. Sci..

[13] Zhou H., Zhou W., Yao X., Zhao Q., Lu L. (2023). Genome-wide investigation and functional analysis reveal that *CsGeBP4* is required for Tea Plant trichome formation. Int. J. Mol. Sci..

[14] Curaba J., Herzog M., Vachon G. (2003). *GeBP*, the first member of a new gene family in *Arabidopsis*, encodes a nuclear protein with DNA-binding activity and is regulated by *KNAT1*. Plant J..

[15] Zhou Y., Zhang Z., Bao Z., Li H., Lyu Y., Zan Y., Wu Y., Cheng L., Fang Y., Wu K. (2022). Graph pangenome captures missing heritability and empowers tomato breeding. Nature.

[16] Wang Y., Sun C., Ye Z., Li C., Huang S., Lin T. (2024). The genomic route to tomato breeding: Past, present, and future. Plant Physiol..

[17] Fonseca R., Capel C., Nieto-Canseco R., Ortiz-Atienza A., Bretones S., López-Fábregas J.D., Quevedo-Colmena A.S., Lebrón R., Barragán-Lozano T., Villalobos-Ramírez V. (2022). A tomato EMS-mutagenized population provides new valuable resources for gene discovery and breeding of developmental traits. Plants.

[18] Molla K.A., Yang Y. (2019). CRISPR/Cas-mediated base editing: Technical considerations and practical applications. Trends Biotechnol..

[19] Egea I., Estrada Y., Flores F.B., Bolarín M.C. (2022). Improving production and fruit quality of tomato under abiotic stress: Genes for the future of tomato breeding for a sustainable agriculture. Environ. Exp. Bot..

[20] Tripodi P., Soler S., Campanelli G., Díez M., Esposito S., Sestili S., Figàs M.R., Leteo F., Casanova C., Platani C. (2021). Genome wide association mapping for agronomic, fruit quality, and root architectural traits in tomato under organic farming conditions. BMC Plant Biol..

[21] Ahmed N., Zhang B., Bozdar B., Chachar S., Rai M., Li J., Li Y., Hayat F., Chachar Z., Tu P. (2023). The power of magnesium: Unlocking the potential for increased yield, quality, and stress tolerance of horticultural crops. Front. Plant Sci..

[22] Bai Y., Kissoudis C., Yan Z., Visser R., van der Linden G. (2018). Plant behaviour under combined stress: Tomato responses to combined salinity and pathogen stress. Plant J..

[23] Wang F., Chen X., Dong S., Jiang X., Wang L., Yu J., Zhou Y. (2020). Crosstalk of PIF4 and DELLA modulates CBF transcript and hormone homeostasis in cold response in tomato. Plant Biotechnol. J..

[24] Mauxion J., Chevalier C., Gonzalez N. (2021). Complex cellular and molecular events determining fruit size. Trends Plant Sci..

[25] Chen Y., Tang X., Fei Z., Giovannoni J. (2024). Fruit ripening and postharvest changes in very early-harvested tomatoes. Hortic. Res..

[26] Dong H., Wang J., Song X., Hu C., Zhu C., Sun T., Zhou Z., Hu Z., Xia X., Zhou J. (2023). HY5 functions as a systemic signal by integrating BRC1-dependent hormone signaling in tomato bud outgrowth. Proc. Natl. Acad. Sci. USA.

[27] Tian F., Yang D., Meng Y., Jin J., Gao G. (2019). PlantRegMap: Charting functional regulatory maps in plants. Nucleic Acids Res..

[28] Santnerr A., Esteller M. (2009). Recent advances and emerging trends in plant hormone signalling. Nature.

[29] Santnerr A., Calderon-Villalobosr L., Esteller M. (2009). Plant hormones are versatile chemical regulators of plant growth. Nat. Chem. Biol..

[30] Wengr J., Yer M., Lir B., Noelr J. (2016). Co-evolution of hormone metabolism and signaling networks expands plant adaptive plasticity. Cell.

[31] Ali M., Luo D., Khan A., Haq S., Gai W., Zhang H., Cheng G., Muhammad I., Gong Z. (2018). Classification and genome-wide analysis of chitin-binding proteins gene family in pepper (*Capsicum annuum* L.) and transcriptional regulation to *Phytophthora capsici*, abiotic stresses and hormonal applications. Int. J. Mol. Sci..

[32] Yu C., Cai X., Ye Z., Li H. (2015). Genome-wide identification and expression profiling analysis of trihelix gene family in tomato. Biochem. Biophys. Res. Commun..

[33] Cui B., Yu M., Bai J., Zhu Z. (2023). SlbHLH22-induced hypertrophy development is related to the salt stress response of the *GTgamma* gene in tomatoes. Metabolites.

[34] Wang Z., Xu C., Benning C. (2012). TGD4 involved in endoplasmic reticulum-to-chloroplast lipid trafficking is a phosphatidic acid binding protein. Plant J..

[35] Fan J., Zhai Z., Yan C., Xu C. (2015). Arabidopsis TRIGALACTOSYLDIACYLGLYCEROL5 interacts with TGD1, TGD2, and TGD4 to facilitate lipid transfer from the endoplasmic reticulum to plastids. The Plant Cell.

[36] Wang Q., Zhan X. (2024). Elucidating the role of *SlBBX31* in plant growth and heat-stress resistance in tomato. Int. J. Mol. Sci..

[37] Fang H., Zhang X., Chen W., Xu L., Yao J., Pei Y., Zhang Y. (2024). Dynamic changes of endogenous H_2_S generation during responding to developmental and environmental signals in *Solanum lycopersicum* L.. Sci. Hortic..

[38] Rowarth N., Curtis B., Einfeldt A., Archibald J., Lacroix C., Gunawardena A. (2021). RNA-Seq analysis reveals potential regulators of programmed cell death and leaf remodelling in lace plant (*Aponogeton madagascariensis*). BMC Plant Biol..

[39] Alves M., Dadalto S., Gonçalves A., de Souza G., Barros V., Fietto L. (2014). Transcription factor functional protein-protein interactions in plant defense responses. Proteomes.

[40] Muhlenbock P., Plaszczyca M., Plaszczyca M., Mellerowicz E., Karpinsk S. (2007). Lysigenous aerenchyma formation in *Arabidopsis* is controlled by *LESION SIMULATING DISEASE1*. Plant Cell.

[41] Kaminaka H., Näke C., Epple P., Dittgen J., Schütze K., Chaban C., Holt B., Merkle T., Schäfer E., Harter K. (2006). bZIP10-LSD1 antagonism modulates basal defense and cell death in *Arabidopsis* following infection. EMBO J..

[42] Perazza D., Laporte F., Balagué C., Chevalier F., Remo S., Bourge M., Larkin J., Herzog M., Vachon G. (2011). GeBP/GPL transcription factors regulate a subset of *CPR5*-dependent processes. Plant Physiol..

[43] Ma C., Chen Q., Wang S., Lers A. (2021). Downregulation of GeBP-like α factor by *MiR827* suggests their involvement in senescence and phosphate homeostasis. BMC Biol..

[44] Zhao M., Li M., Zhang L., Wu N., Tang X., Yang X., Ghanem H., Wu M., Wu G., Qing L. (2025). Insights into geminiviral pathogenesis: Interaction between βC1 protein and *GLABROUS1* enhancer binding protein (GeBP) in Solanaceae. Phytopathol. Res..

[45] García-Cano E., Hak H., Magori S., Lazarowitz S., Citovsky V. (2018). The *Agrobacterium* F-box protein effector VirF destabilizes the *Arabidopsis* GLABROUS1 enhancer/binding protein-like transcription factor VFP4, a transcriptional activator of defense response genes. Mol. Plant-Microbe Interact..

[46] Wu J., Liu R., Xie Y., Zhao S., Yan M., Sun N., Zhan Y., Li F., Yu S., Feng Z. (2024). Association of *GhGeBP* genes with fiber quality and early maturity related traits in upland cotton. BMC Genom..

[47] Simm S., Fragkostefanakis S., Paul P., Keller M., Einloft J., Scharf K., Schleiff E. (2015). Identification and expression analysis of ribosome biogenesis factor co-orthologs in *Solanum lycopersicum*. Bioinform. Biol. Insights.

[48] Lu Y., Ma L., Cheng K., Li J., Tang H., Zhu G., Wen H., Zhu B., Fu D., Qu G. (2025). Comprehensive identification of ripening-related RNA-binding proteins in tomatoes using improved plant phase extraction. Plant J..

[49] Xiong F., Dong P., Liu M., Xie G., Wang K., Zhuo F., Feng L., Yang L., Li Z., Ren M. (2016). Tomato FK506 binding protein 12KD (FKBP12) mediates the interaction between rapamycin and target of rapamycin (TOR). Front. Plant Sci..

[50] Khan I., Lubna, Asaf S., Jan R., Bilal S., Khan A., Kim K., Al-Harrasi A. (2023). Dynamic interplay of *WRKY*, *GRAS*, and *ERF* transcription factor families in tomato-endophytic fungal symbiosis: Insights from transcriptome and genome-wide analysis. Front. Plant Sci..

[51] Ahouvi Y., Haber Z., Zach Y., Rosental L., Toubiana D., Sharma D., Alseekh S., Tajima H., Fernie A., Brotman Y. (2022). The alteration of tomato chloroplast vesiculation positively affects whole-plant source–sink relations and fruit metabolism under stress conditions. Plant Cell Physiol..

[52] Chew B., Fisk I., Fray R., Tucker G., Bodi Z., Ferguson A., Xia W., Seymour G. (2017). The effect of adenosine monophosphate deaminase overexpression on the accumulation of umami-related metabolites in tomatoes. Plant Cell Rep..

[53] Zhang J., Jia W., Yang J., Ismail A. (2006). Role of ABA in integrating plant responses to drought and salt stresses. Field Crop Res..

[54] Huber A., Melcher P., Piñeros M., Setter T., Bauerle T. (2019). Signal coordination before, during and after stomatal closure in response to drought stress. New Phytol..

[55] Zhang H., Zhu J., Gong Z., Zhu J. (2022). Abiotic stress responses in plants. Nat. Rev. Genet..

[56] Su D., Xiang W., Wen L., Lu W., Shi Y., Liu Y., Li Z. (2021). Genome-wide identification, characterization and expression analysis of BES1 gene family in tomato. BMC Plant Biol..

[57] Cao H., Chen J., Yue M., Xu C., Jian W., Liu Y., Song B., Gao Y., Cheng Y., Li Z. (2020). Tomato transcriptional repressor MYB70 directly regulates ethylene-dependent fruit ripening. Plant J..

