# Supplementary material for: Genome-Wide Identification and Expression Profiling of SlGeBP Gene Family in Response to Hormone and Abiotic Stresses in Solanum lycopersicum L
Source: Int J Mol Sci. 2025 Jun 23;26(13):6008. doi: 10.3390/ijms26136008 (PMC12250332; doi:10.3390/ijms26136008)
Supplement: Supplementary file 1 [file ijms-26-06008-s001.zip › Supplementary Figures.pdf]

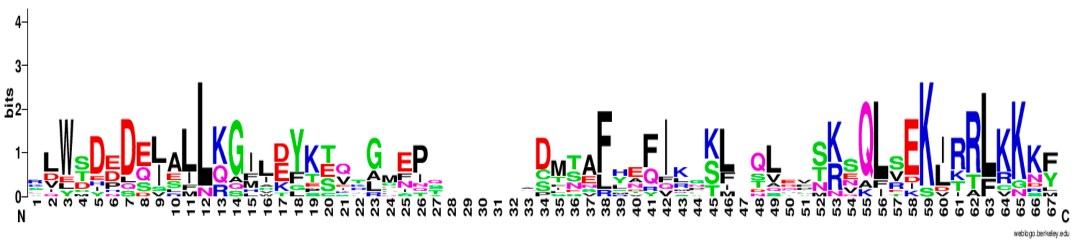

Figure S1 Sequence logos of amino acid multiple sequence alignment of SlGeBPs.

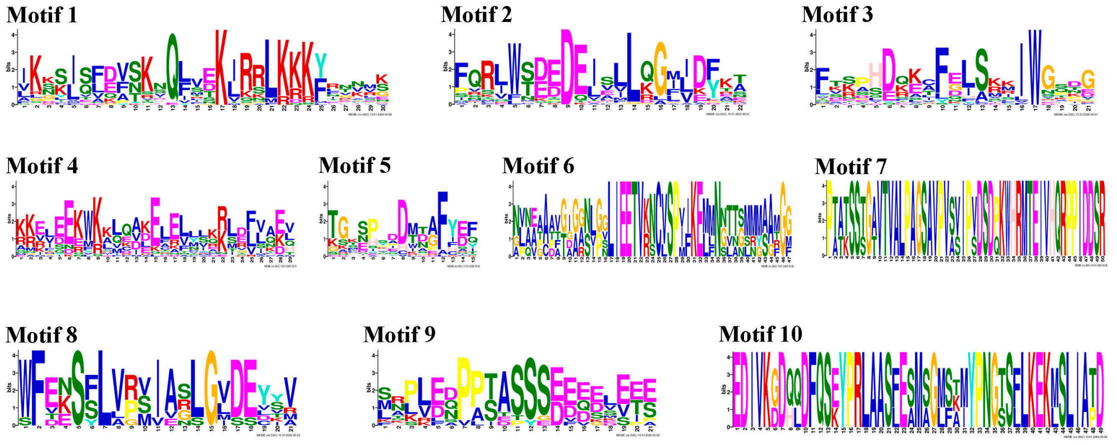

Figure S2 The conserved motif analyzed by MEME tool.

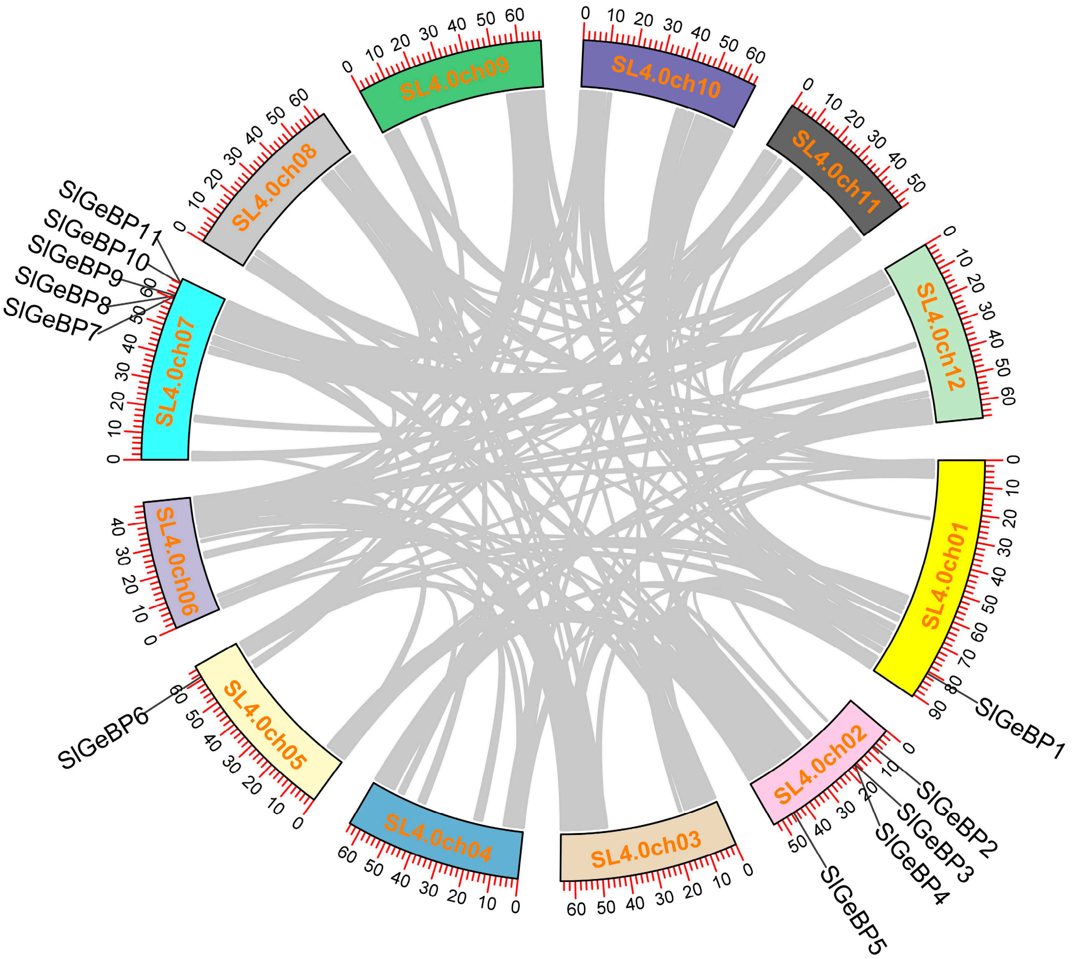

Figure S3 Chromosome location and intraspecies collinearity analysis of *SlGeBP* gene family in tomato genome. Boxes with different colors represent different chromosomes. The gray lines represent the collinearity relationship among different genes in tomato.

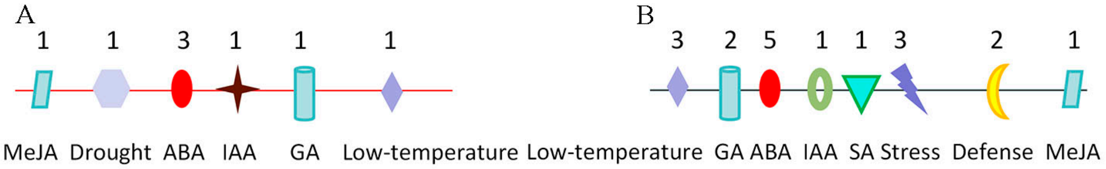

Figure S4 Types and quantities of *cis-acting* elements in the promoter region related to hormone and stress responses in *SlGeBP1* (A) and *SlGeBP5* (B).

Identifier	Species	Strain	Cultivar	Organ Type	Organ	Tissue	Stage
**C1**	S lyco	Wild Type	Micro.Tom	Reproductive	Seed	Whole	IMG_10DPA
**C2**	S lyco	Wild Type	Micro.Tom	Reproductive	Seed	Whole	MG_35DPA
**C3**	S lyco	Wild Type	Micro.Tom	Reproductive	Seed	Whole	Breaker_38DPA
**C4**	S lyco	Wild Type	Micro.Tom	Reproductive	Seed	Whole	Orange_41dpa
**C5**	S lyco	Wild Type	Micro.Tom	Reproductive	Seed	Whole	Red_44DPA
**C6**	S lyco	Wild Type	Micro.Tom	Vegetative	Root	Whole	nc
**C7**	S lyco	Wild Type	Micro.Tom	Vegetative	Leaf	Whole	nc
**C8**	S lyco	Wild Type	Micro.Tom	Reproductive	Flower	Petal	nc
**C9**	S lyco	Wild Type	Micro.Tom	Reproductive	Flower	Whole	Bud
**C10**	S lyco	Wild Type	Micro.Tom	Reproductive	Flower	Whole	Bud.3.mm
**C11**	S lyco	Wild Type	Micro.Tom	Reproductive	Flower	Whole	Anthesis
**C12**	S lyco	Wild Type	Micro.Tom	Reproductive	Fruit	Whole	4.dpa
**C13**	S lyco	Wild Type	Micro.Tom	Reproductive	Fruit	Flesh	IMG_10DPA
**C14**	S lyco	Wild Type	Micro.Tom	Reproductive	Fruit	Peel	IMG_10DPA
**C15**	S lyco	Wild Type	Micro.Tom	Reproductive	Fruit	Flesh	MG_35DPA
**C16**	S lyco	Wild Type	Micro.Tom	Reproductive	Fruit	Peel	MG_35DPA
**C17**	S lyco	Wild Type	Micro.Tom	Reproductive	Fruit	Flesh	Breaker_38DPA
**C18**	S lyco	Wild Type	Micro.Tom	Reproductive	Fruit	Peel	Breaker_38DPA
**C19**	S lyco	Wild Type	Micro.Tom	Reproductive	Fruit	Flesh	Orange_41dpa
**C20**	S lyco	Wild Type	Micro.Tom	Reproductive	Fruit	Peel	Orange_41dpa
**C21**	S lyco	Wild Type	Micro.Tom	Reproductive	Fruit	Flesh	Red_44DPA
**C22**	S lyco	Wild Type	Micro.Tom	Reproductive	Fruit	Peel	Red_44DPA

Figure S5 22 different tissues from TomExpress online RNA-seq date.

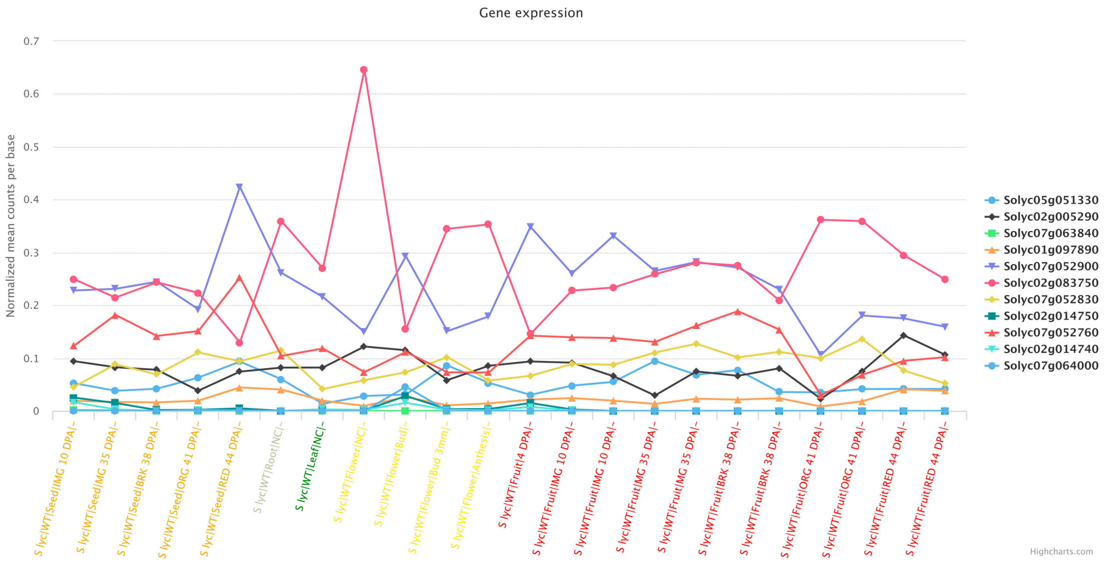

Figure S6 Graphical presentation of SlGeBP expression datas produced by the Tomexpress web server.

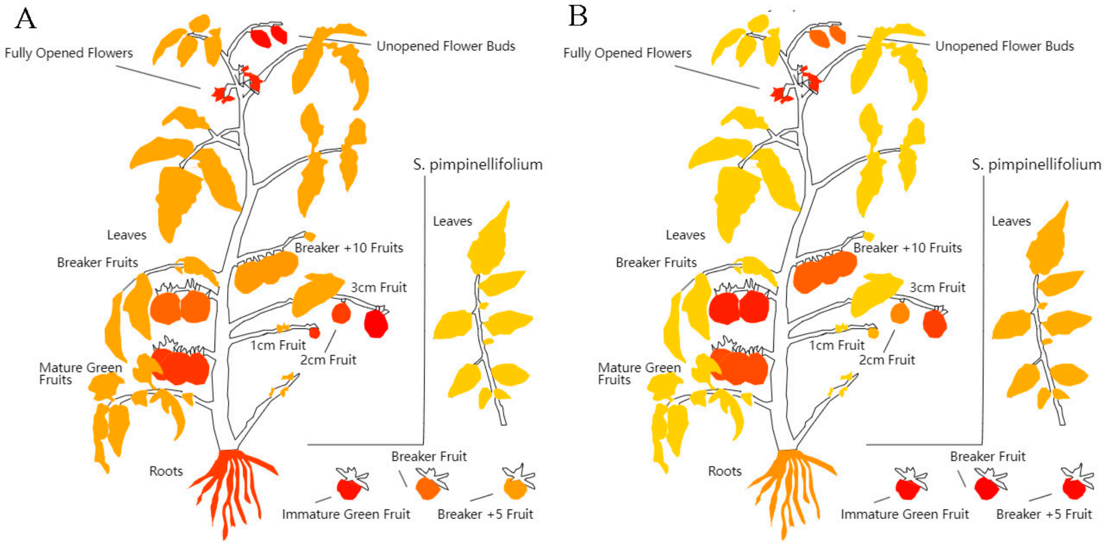

Figure S7 Predicted the tissue expression patterns of SlGeBP1 (A) and SlGeBP5 (B) in ePlant.

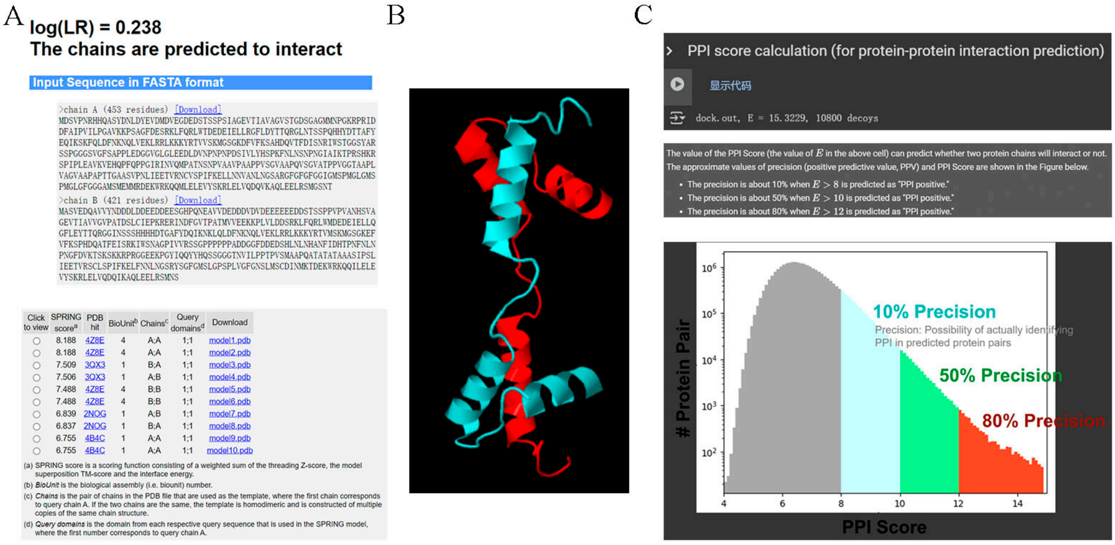

Figure S8 The predicted interaction with SlGeBP1 and SlGeBP5 in PEPPI and MEGADOCK. (A) The log(LR) score and output result in PEPPI showed SlGeBP1 and SlGeBP5 were predicted to interact. (B) The presumptive protein complex formed by SlGeBP1 and SlGeBP5 in PEPPI. (C) The predicted E-value and the annotation of the probability in MEGADOCK.
