# Supplementary material for: Genome-Wide Identification and Expression Profiling of SlGeBP Gene Family in Response to Hormone and Abiotic Stresses in Solanum lycopersicum L
Source: Int J Mol Sci. 2025 Jun 23;26(13):6008. doi: 10.3390/ijms26136008 (PMC12250332; doi:10.3390/ijms26136008)
Supplement: Supplementary file 1 [file ijms-26-06008-s001.zip › Table S1 3D model and secondary structure and the predict subcellular localization of SlGeBPs in ePlant.pdf]

	3D Structure	Secondary Structure	Subcellular Location
SlGeBP1	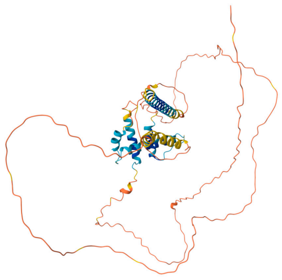	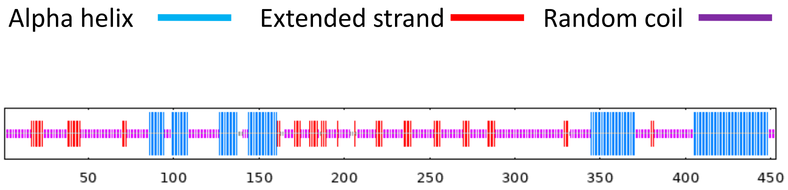	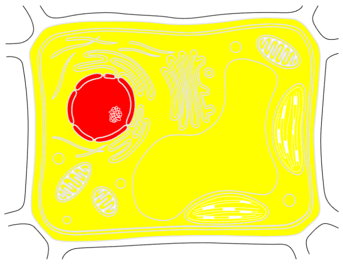
SlGeBP2	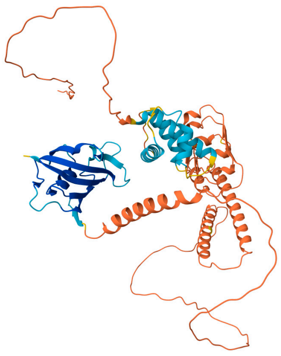	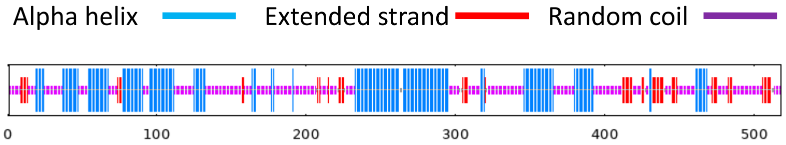	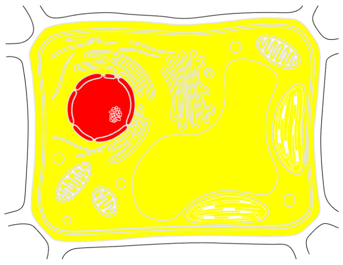
SlGeBP3	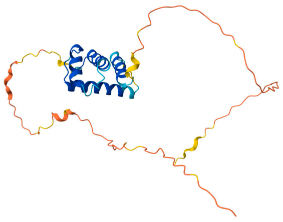	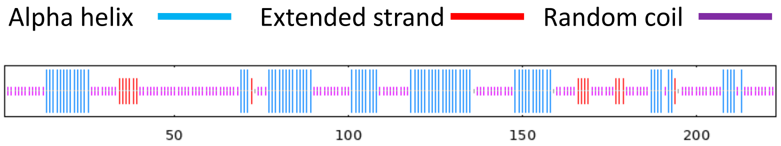	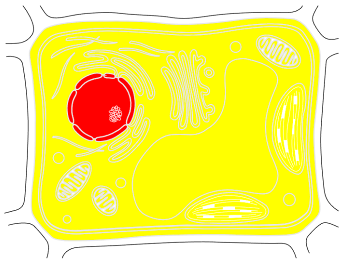
SlGeBP4	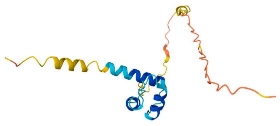	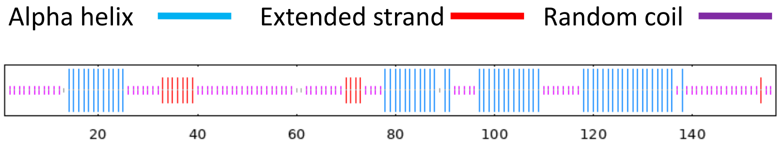	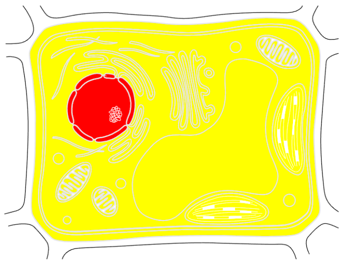
SlGeBP5	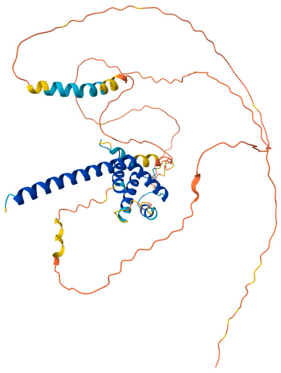	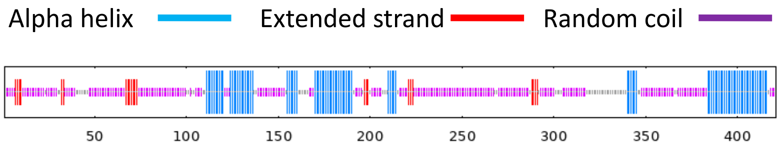	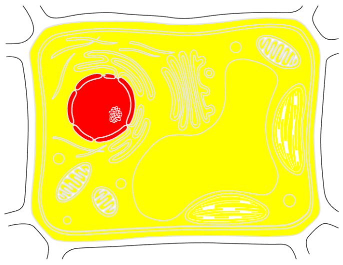
SlGeBP6	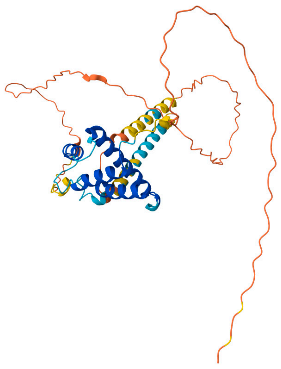	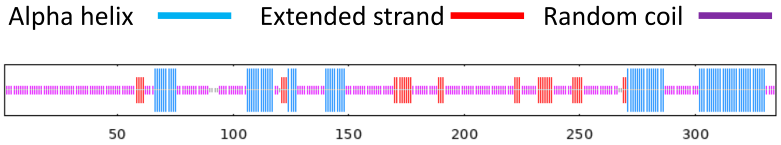	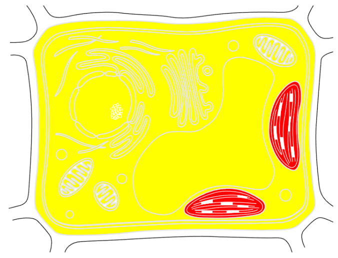

SlGeBP7	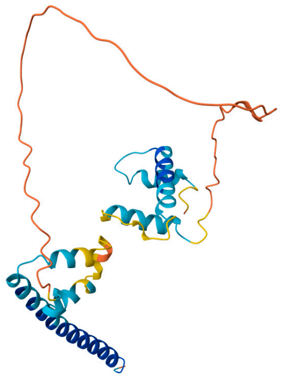	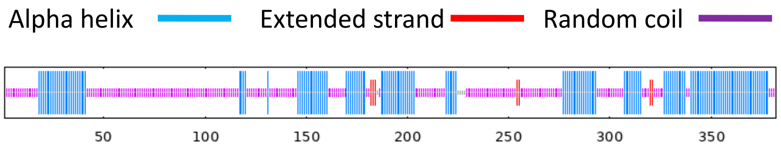	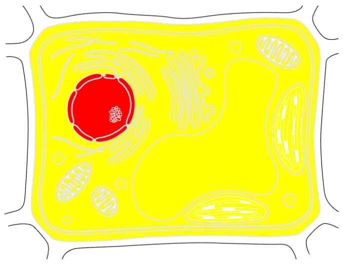
SlGeBP8	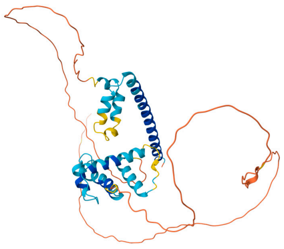	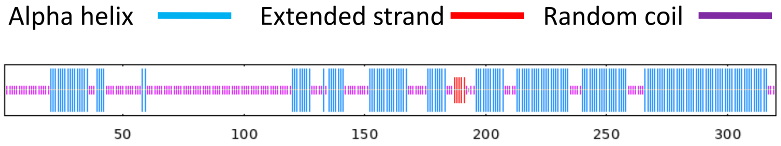	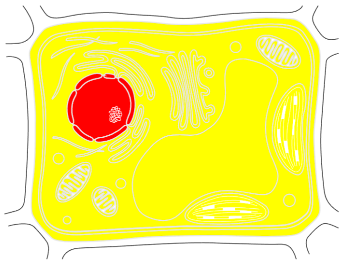
SlGeBP9	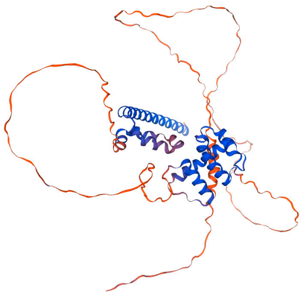	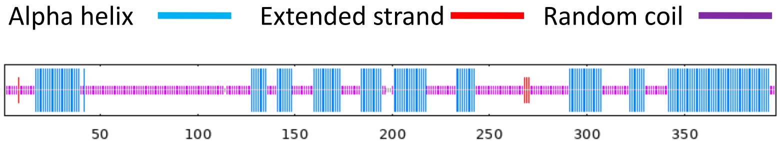	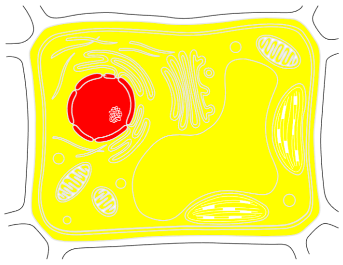
SlGeBP10	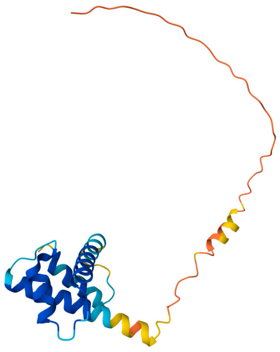	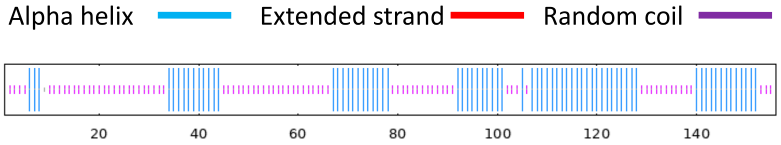	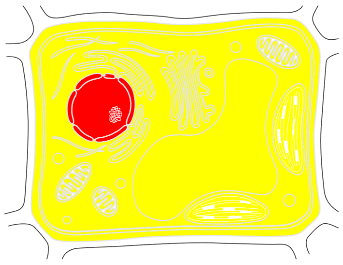
SlGeBP11	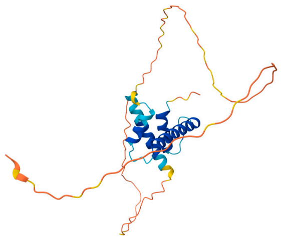	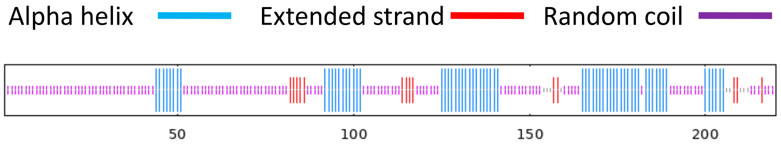	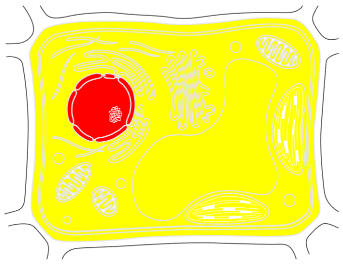
