# Supplementary material for: Genome-Wide Identification and Expression Profiling of SlGeBP Gene Family in Response to Hormone and Abiotic Stresses in Solanum lycopersicum L
Source: Int J Mol Sci. 2025 Jun 23;26(13):6008. doi: 10.3390/ijms26136008 (PMC12250332; doi:10.3390/ijms26136008)
Supplement: Supplementary file 1 [file ijms-26-06008-s001.zip › Table S2 Predicted subcellular location of SlGeBP family protein.pdf]

Table S2. Predicted subcellular location of SlGeBP family protein

Gene name	Wolf PSort	BaCelLo	CELLO	ePlant
SlGeBP1	nucl: 11	Nucleus	Nuclear（2.171）	Nucleus
SlGeBP2	nucl: 11	Nucleus	Nuclear（4.252）	Nucleus
SlGeBP3	nucl: 6.5, cyto_nucl: 6.5, cyto: 5.5	Nucleus	Nuclear（4.796）	Nucleus
SlGeBP4	chlo: 5, nucl: 4.5, cyto_nucl: 3.5, extr: 3	Nucleus	Nuclear（3.998）	Nucleus
SlGeBP5	nucl: 11	Nucleus	Nuclear（4.342）	Nucleus
SlGeBP6	nucl: 7, chlo: 5,	Nucleus	Nuclear（2.938）	plastid
SlGeBP7	nucl: 12	Nucleus	Nuclear（4.064）	Nucleus
SlGeBP8	nucl: 12	Nucleus	Nuclear（3.599）	Nucleus
SlGeBP9	nucl: 10	Nucleus	Nuclear（4.095）	Nucleus
SlGeBP10	nucl: 9	Nucleus	Nuclear（3.999）	Nucleus
SlGeBP11	nucl: 7	Nucleus	Nuclear（3.817）	Nucleus
