# Supplementary material for: Genome-Wide Identification and Expression Profiling of SlGeBP Gene Family in Response to Hormone and Abiotic Stresses in Solanum lycopersicum L
Source: Int J Mol Sci. 2025 Jun 23;26(13):6008. doi: 10.3390/ijms26136008 (PMC12250332; doi:10.3390/ijms26136008)
Supplement: Supplementary file 1 [file ijms-26-06008-s001.zip › Table S3 Predicted the protein-protein interaction in SlGeBPs.pdf]

SlGeBP2 STRING ID: A0A3Q7FFG3 PaxDb ID: Solyc02g005290

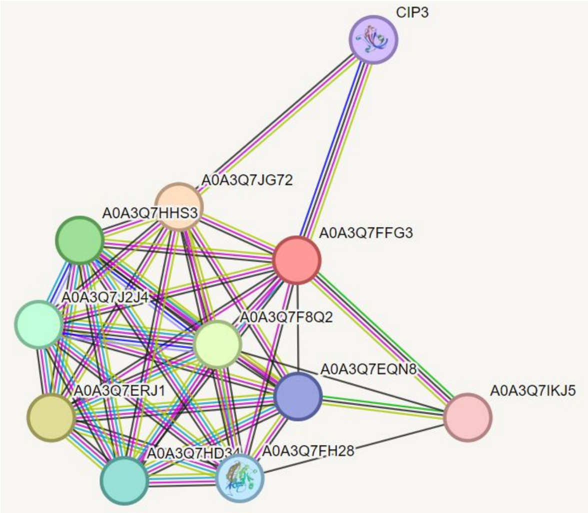

Your Input:

A0A3Q7FFG3 *Peptidylprolyl isomerase. (518 aa)*

Predicted Functional Partners:

		Neighborhood	Gene Fusion	Cooccurrence	Coexpression	Experiments	Databases	Textmining	[Homology]	**Score**
A0A3Q7JG72	*Brix domain-containing protein.*				●	●		●		0.789
A0A3Q7ERJ1	*Ribosome biogenesis protein BOP1 homolog; Required for maturation of ribosomal RNAs and formation of the large ribo…*				●	●				0.730
A0A3Q7F8Q2	*Nop domain-containing protein.*				●	●	●			0.599
A0A3Q7HHS3	*Nop domain-containing protein.*				●	●		●		0.595
A0A3Q7J2J4	*Nop domain-containing protein.*				●	●		●		0.595
A0A3Q7HD34	*Pescadillo homolog; Required for maturation of ribosomal RNAs and formation of the large ribosomal subunit; Belongs t…*				●	●				0.593
A0A3Q7FH28	*Uncharacterized protein.*				●	●				0.587
A0A3Q7EQN8	*Ribosome assembly factor mrt4; Component of the ribosome assembly machinery. Nuclear paralog of the ribosomal pro…*				●					0.574
CIP3	*Peptidylprolyl isomerase.*			●	●	●		●		0.569
A0A3Q7IKJ5	*Inosine-5'-monophosphate dehydrogenase; Catalyzes the conversion of inosine 5'-phosphate (IMP) to xanthosine 5'-pho…*	●			●	●		●		0.568

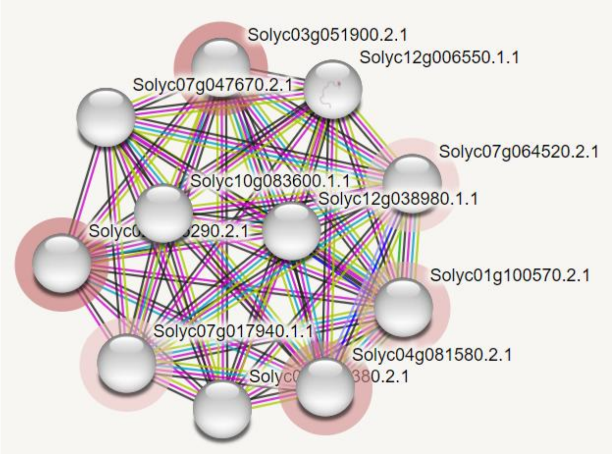

Your Input:

Solyc02g005290.2.1 *Abundance: 75.5 ppm, rank: 3327. out of 7312*

Predicted Functional Partners:

		Neighborhood	Gene Fusion	Cooccurence	Coexpression	Experiments	Databases	Textmining	[Homology]	**Score**
Solyc12g006550.1.1	*Ribosome biogenesis regulatory protein; Involved in ribosomal large subunit assembly*				●	●		●		0.945
Solyc10g083600.1.1	*Ribosome production factor 2 homolog; Ribosomal RNA processing Brix domain protein*				●	●				0.921
Solyc07g064520.2.1	*Abundance: 37.8 ppm, rank: 6260. out of 7312 [bottom 25%]*				●	●	●	●		0.883
Solyc04g016380.2.1	*annotation not available*				●	●				0.864
Solyc03g051900.2.1	*Abundance: 75.5 ppm, rank: 3178. out of 7312*				●	●		●		0.855
Solyc01g100570.2.1	*Abundance: 37.8 ppm, rank: 5423. out of 7312*				●	●				0.852
Solyc07g017940.1.1	*Abundance: 37.8 ppm, rank: 6235. out of 7312 [bottom 25%]*				●	●				0.848
Solyc12g038980.1.1	*H/aca ribonucleoprotein complex subunit 2-like protein; Common component of the spliceosome and rRNA proce…*				●	●				0.843
Solyc07g047670.2.1	*Pescadillo homolog; Required for maturation of ribosomal RNAs and formation of the large ribosomal subunit*				●	●				0.842
Solyc04g081580.2.1	*Abundance: 56.6 ppm, rank: 4462. out of 7312*				●	●	●			0.841

SlGeBP3   STRING ID: A0A3Q7EVX9

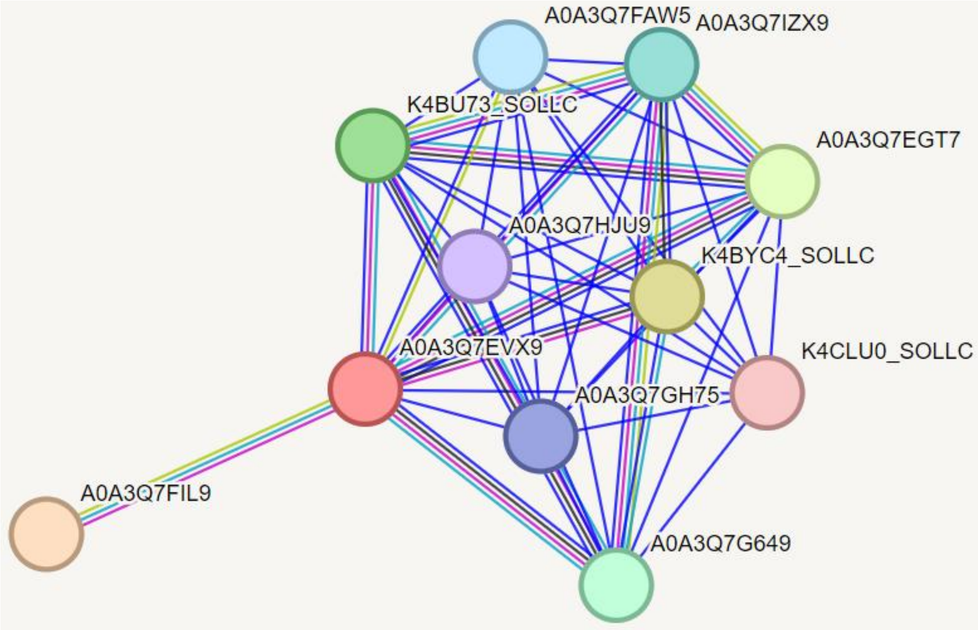

Your Input:

A0A3Q7EVX9	*Uncharacterized protein. (223 aa)*

Predicted Functional Partners:

Protein	Annotation	Neighborhood	Gene Fusion	Cooccurrence	Coexpression	Experiments	Databases	Textmining	[Homology]	Score
A0A3Q7FIL9	*Uncharacterized protein.*					●	●	·		0.641
K4BYC4_SOLLC	*WRKY domain-containing protein.*			●	·	·				0.597
A0A3Q7EGT7	*Uncharacterized protein.*			●	·	·	·			0.559
K4BU73_SOLLC	*NAB domain-containing protein.*			●		·	·			0.557
A0A3Q7G649	*Uncharacterized protein.*			●	·	·	·			0.549
A0A3Q7IZX9	*XS domain-containing protein.*			●		·	·			0.548
A0A3Q7FAW5	*Uncharacterized protein.*			●				·		0.545
A0A3Q7GH75	*Uncharacterized protein.*			●						0.542
A0A3Q7HJU9	*Uncharacterized protein.*			●						0.542
K4CLU0_SOLLC	*Uncharacterized protein.*			●						0.542

SlGeBP6 STRING ID:A0A3Q7HF63

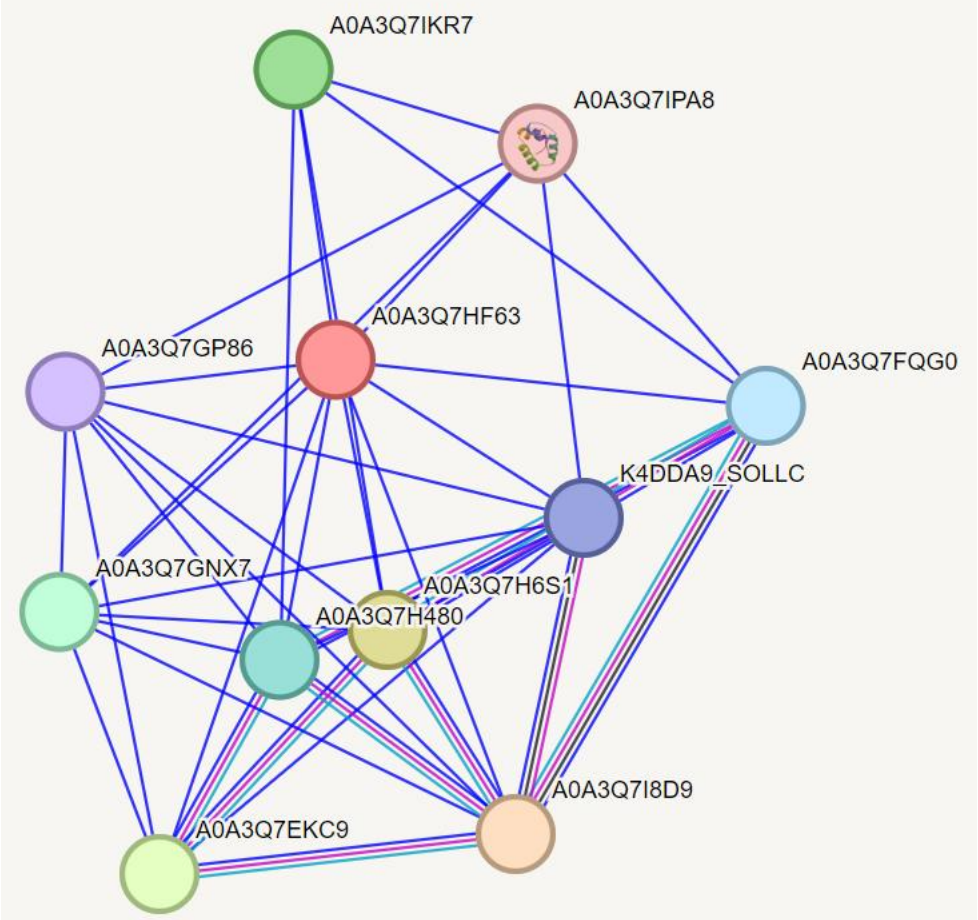

Your Input:

● A0A3Q7HF63	*Uncharacterized protein. (335 aa)*	

Predicted Functional Partners:

Protein	Description	Neighborhood	Gene Fusion	Cooccurrence	Coexpression	Experiments	Databases	Textmining	[Homology]	Score
A0A3Q7I8D9	*Uncharacterized protein.*			●						0.480
A0A3Q7H6S1	*Uncharacterized protein.*			●						0.474
A0A3Q7EKC9	*F-box domain-containing protein.*			●						0.467
A0A3Q7IKR7	*Uncharacterized protein.*			●						0.459
A0A3Q7GNX7	*AAI domain-containing protein; Belongs to the plant LTP family.*			●						0.457
A0A3Q7H480	*Uncharacterized protein.*			●						0.456
A0A3Q7FQG0	*DUF4408 domain-containing protein.*			●						0.455
K4DDA9_SOLLC	*Uncharacterized protein.*			●						0.455
A0A3Q7GP86	*Uncharacterized protein.*			●						0.450
A0A3Q7IPA8	*AAI domain-containing protein.*			●						0.449

SlGeBP7 STRING ID:A0A3Q7HBY0 PaxDb ID: Solyc07g052760

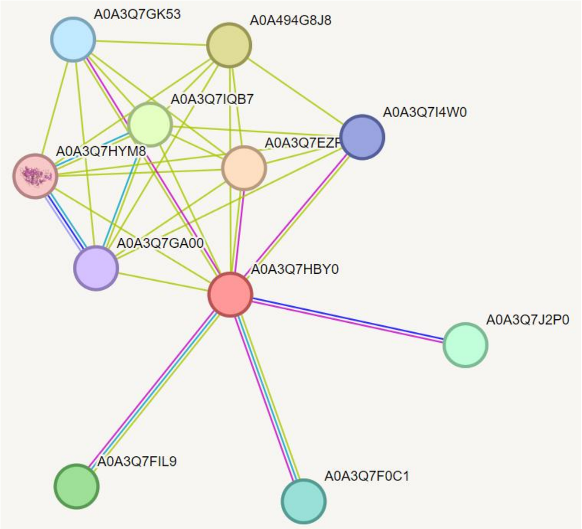

Your Input:

A0A3Q7HBY0	*Uncharacterized protein. (218 aa)*

Predicted Functional Partners:

Protein	Annotation	Neighborhood	Gene Fusion	Cooccurrence	Coexpression	Experiments	Databases	Textmining	[Homology]	Score
A0A3Q7EZF8	*ALA-interacting subunit; Belongs to the CDC50/LEM3 family.*					•		•		0.717
A0A494G8J8	*Uncharacterized protein.*							•		0.715
A0A3Q7IQB7	*Uncharacterized protein.*							•		0.712
A0A3Q7FIL9	*Uncharacterized protein.*					•	•	•		0.641
A0A3Q7J2P0	*CCHC-type domain-containing protein.*			•		•				0.589
A0A3Q7F0C1	*ERCC4 domain-containing protein.*					•	•	•		0.539
A0A3Q7GK53	*Maturase K; Usually encoded in the trnK tRNA gene intron. Probably assists in splicing its own and other chloroplast gro...*					•		•		0.538
A0A3Q7I4W0	*Maturase K; Usually encoded in the trnK tRNA gene intron. Probably assists in splicing its own and other chloroplast gro...*					•		•		0.538
A0A3Q7GA00	*A_deaminase domain-containing protein.*							•		0.530
A0A3Q7HYM8	*A_deaminase domain-containing protein.*							•		0.530

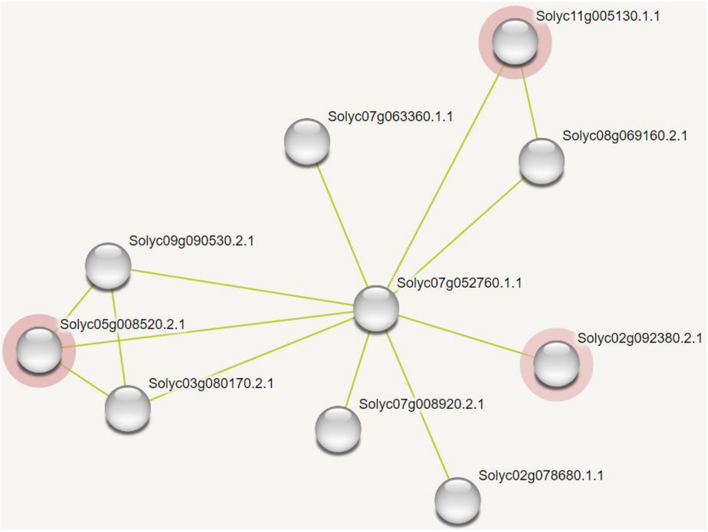

Your Input:

Solyc07g052760.1.1	*annotation not available (382 aa)*

Predicted Functional Partners:

Protein	Annotation	Neighborhood	Gene Fusion	Cooccurence	Coexpression	Experiments	Databases	Textmining	[Homology]	Score
Solyc08g069160.2.1	*Trihelix transcription factor asr3; Uncharacterized protein; Sequence-specific DNA binding transcription factors*							•		0.590
Solyc07g008920.2.1	*Oxysterol-binding protein-related protein 4c; Belongs to the OSBP family*							•		0.529
Solyc02g078680.1.1	*annotation not available*							•		0.491
Solyc02g092380.2.1	*Abundance: 37.8 ppm, rank: 5689. out of 7312 [bottom 25%]*							•		0.491
Solyc07g063360.1.1	*annotation not available*							•		0.491
Solyc03g080170.2.1	*annotation not available*							•		0.437
Solyc05g008520.2.1	*Abundance: 37.8 ppm, rank: 4885. out of 7312*							•		0.437
Solyc09g090530.2.1	*Histone h2a.z-specific chaperone chz1; Unknown protein; BEST Arabidopsis thaliana protein match is: unknown pr...*							•		0.437
Solyc11g005130.1.1	*Abundance: 37.8 ppm, rank: 5039. out of 7312*							•		0.424

SlGeBP8 STRING ID:A0A3Q7I8D9

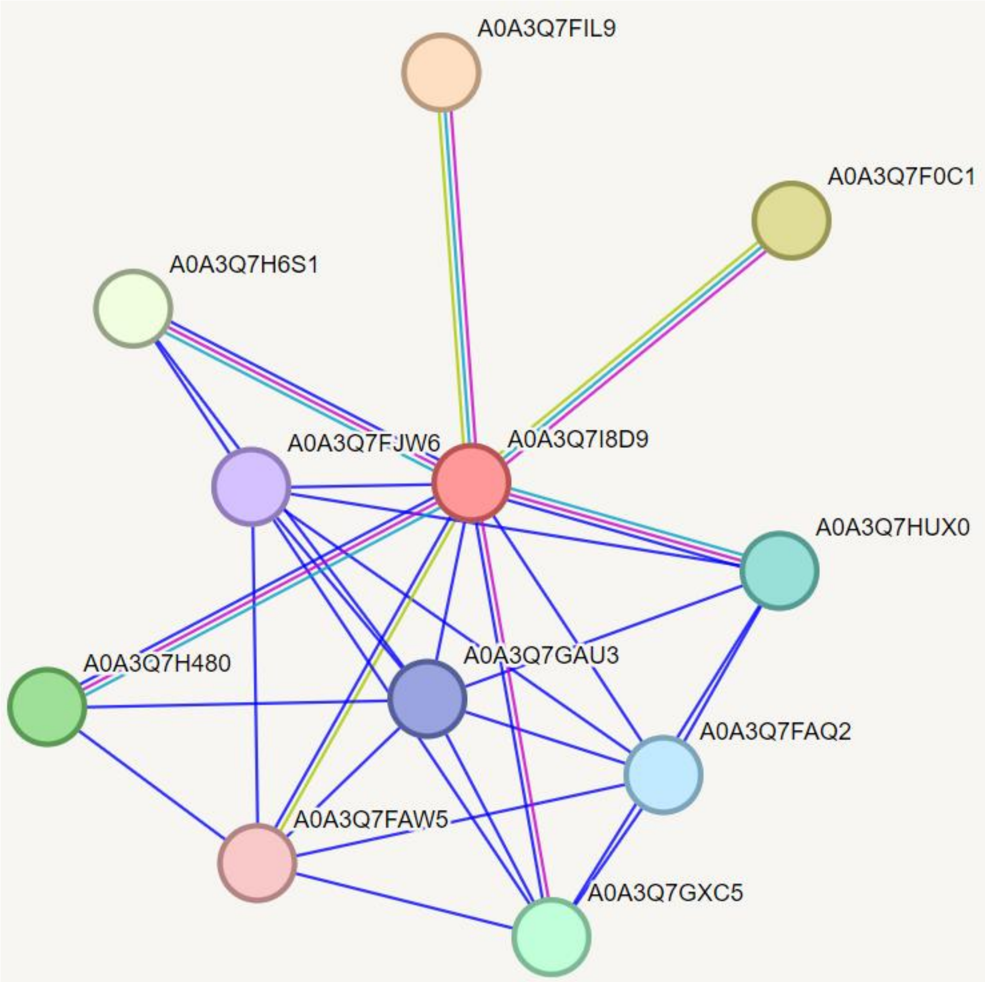

Your Input:

A0A3Q7I8D9 *Uncharacterized protein. (359 aa)*

Predicted Functional Partners:

		Neighborhood	Gene Fusion	Cooccurrence	Coexpression	Experiments	Databases	Textmining	[Homology]	Score
A0A3Q7FIL9	*Uncharacterized protein.*					●	●	●		0.641
A0A3Q7F0C1	*ERCC4 domain-containing protein.*					●	●	●		0.539
A0A3Q7H6S1	*Uncharacterized protein.*			●		●	●			0.526
A0A3Q7H480	*Uncharacterized protein.*			●		●	●			0.525
A0A3Q7GXC5	*Uncharacterized protein.*			●		●				0.520
A0A3Q7HUX0	*Uncharacterized protein.*			●		●	●			0.516
A0A3Q7FAQ2	*Uncharacterized protein.*			●						0.515
A0A3Q7GAU3	*Uncharacterized protein.*			●						0.514
A0A3Q7FJW6	*Uncharacterized protein.*			●						0.510
A0A3Q7FAW5	*Uncharacterized protein.*			●				●		0.509
