# Supplementary material for: Genome-Wide Identification and Expression Profiling of SlGeBP Gene Family in Response to Hormone and Abiotic Stresses in Solanum lycopersicum L
Source: Int J Mol Sci. 2025 Jun 23;26(13):6008. doi: 10.3390/ijms26136008 (PMC12250332; doi:10.3390/ijms26136008)
Supplement: Supplementary file 1 [file ijms-26-06008-s001.zip › Table S4 Amino acid sequences of 97 GeBPs from 6 species.pdf]

## Arabidopsis (*Arabidopsis thaliana*)

>AT1G11510.1
MSRRFNPLEDPPSASSSDEEGKEIARNSSSDDEDDVSSENPSPLKTTLDAVSDSESGSDEETDSDSELEKKKDQV
VTNPVDVKRAKKVKTSEKSGAKRSLEVDEAAVSMDVKRAKKVSGEEEKKKSGGGGEETKKTYFQRLWTEDDEI
VVLQGLIDDKKDTGVSNTNKVYELVKKSISFDVSKNQLMEKLRALKKKYENNLGKAKDGVEPTFVKPHDRKAFE
LSKLVWGGIRMALASGMKSNEKSKKSSKFESVKHELDSSLPNSKNNCEDEVMDEGEVSFTKSSLVRSIVGLGM
DELTAQQGLSKLASKDMKRFYEQWKAMQAREFEFFLQKHGFLFEVLSKISEAFGSNA
>AT1G44810.1
MNKKLLNPLEDPPTASSSEDVDEEISSGEDEKEHISNSSSSEEENELKDLSTQTLNSPSTEAPTLDSGSETNSDSDK
PIVLTSQKKKEGTDSSGTKRASEGTSSKDIKRAKKVSGDDDNKKFQSLWTKEDEISLLQGMIDFKAETGTSAHDD
MNGFFDIAKRYISFDVSKIQFGDKIRGLKKKYFGVRKKKGLDLDHDKKCLGLAKSIWGLDGKEVVVLGGDSETSN
WFEKSFLVRVVARLGVDECIVKWKWSKVSKETKKRIEEKMKMVEAKELELLSQKIDVLKEVASVIAETI
>AT1G55950.1
MAKKNKRSQQKNKCLKPEKDPSTVKRLLEDPPRKKRYLQPLENPPTESYGDDEKMETEEEGEKYLGKNLPASAF
IIVAAERHDSDSYSETELEKKICGSVVVAKMNDDKMVSTDTKKKYFQRILSDDDKIVLLQGMVDFQNDKGTISY
DDMTGFIDTVKNIISFQANSRQFTTKIRRLKDKFVQKRNKGVDENSLANVEIYEI
>AT1G61730.1
MTKKLNPLEDPPTATSSDEDDVETSEAGEASDDSSSSEEDVPIKIRIKSPSATTAAAPPAKSTAVSTAADSDSGSET
ETDSDSESTNPPNSGSGKTIALNTVNLKKKEDPTSSSATLALPAMKSGTKRPASEAAATTSTKRVKKDEESVKKPG
GFQRLWSEEDEILVLQGMIDFKADTGKSPYVDTNAFYDFLKKSISFEVSKNQFMDKIRSLRKKYIGKEGRNEPSF
VKAHDKKAFELSKFIWGPKGIALDSNVKSNGVSKKSVAKKKIDSVKQELVFAGGSSTNGKKVEEDGGDDGCDW
FDNSSLVRMIASLGVDEYYVKQQWSLVSVESKKIVEEKYKLLQAKELEFVLEKTKFLNEVASMFVEASKNKPLDT
>AT1G66420.1
MSKKHLKPLETCFEDEEDDVIYLPPGLVTGATSKKNEEFCGGSGKVQPSEMKRRSEGTSTDMTSKRAKKVSAED
EKKVGEWTKNPYFQRLWSEEDEIVMLQGIIKFEDVTGKSPFEDRHGFIEFVKNSISFEASVQQYIGKISQLKRKYT
RKRKNGFSEGHEQKCFKLAMSIWGTKETSKKTDLCSSPKGKKVKEEDGDVTNSDWFENSFLLPSIESLGVDSVK
RKWNVVPMEFKKKIEERLELLEADESECKKMEEMLKVKNSECVKQKTNLLNEVIDVMT
>AT2G01370.1
MKGITKKLLVSRSLKRLTAKKNRRKIHRETNKRLKPEEDPSMVKRRQDSDSDSLEKEVSVVVTKTSDDEMVSTDT
KKNYFQRIWSDDDEIVLLQGMVDFENDKGKSPYDDMTGFIDTVKNFISFQANQHQFTTKIRRLKDKFLKKWN
NGVDENSLVNAHDRKCFQLSKVIWEPTTKVEEVGDWFVNSYLVGSIARLGVSEDVMKQSWSMVPIETKKEVE
EKFKLLKDEESENLRLKTSFLQKVNSMIIEATN
>AT2G20613.1
DEVVTEVSALNNQESKSVKISEKSVAKRSRETHEAQASADVKKAKKVKKRGGGGGEEAETKKAYFQRVWTDDD
EIVVLEGFIDYKNDSG
>AT2G25650.1
MVTPKQIDFSSCGGGDNSDDTLSHRESPRNPSSKRAAASFAAEAGEETMKKKKKKKKNLGPPLIVRIWNEED
ELSILKGLVDYRAKTGFNPKIDWDAFYSFLGSSIVAKFSKEQVLSKIRKLKRRFHVHWEKISEGNDPKFTRSSDSEA
FGFSSMIWGQGEFGNDDGMDKEMVKEHDVNGNGAAENGTARIAQENESGEEMLKEHEETLNENGAEEIRD
NDETARKAQQLESESEEEMLKEHEEPFNENGAENIRDNNGTTQIAQQSESESEEMLKEHEEVANTELVNENGA
AKTTENGTTGGKERHDDDDDDELCAVQDAFEAVMSQGLSGYQKKLQLEKLMNLGTGKRRELSDEWKALCVE
ERRLNIKKLRFSAKLAEAANDS
>AT2G36340.1
MVGTKRVADSIDTNSDDTLTRNREVEVEAMLSRRKQLRTTTTRTTTTRTTPLSLSSSASKMNWSKNDELVILGGI

VDYENETKLSYRSDWDALYRYIKDCVEAKFSKIQLINKVKNMKRKFTYNQGRSNHGEQLSFTNTDDDEIFKLSLII
WDKNESEYVSNENIDQAKDVPSGEPETNDVPCEEQDDRDVPCEEQERANIEIDNGVREKLDQAKDVPCVEQE
SEDVPCVEQERVSIEIDNGEKEKLDQTMDCEEQENTDVLCEEKGDKDVPCEEQENKDVPCEEQERVSIEIDNGE
EEMSSEEDGVDEVGVMEDTLDSGISFQGLGKNGVKDKSEEDDVVELGVLQEIFKEDTFFQSLGRYQQKLLLQN
LENVGVERRKELINEWKALFVDEQRLCVKKLTFAAKLANLGVSP
>AT3G04930.1
MTSDHRDALFSLELESPDPDEGGVADGGESDTDEDLRDNDDVVMPGTNEAEAEDDDPEEEDLNSPSTSSLPM
VSTISATAVVSGTPTATSSTGAVTVALPAGSAVPVSVIPVDSDPKWHRMTEIVHQRPPIDDSRRLFQRLWTDEDE
IELLRGFLDYMTMHRGSSSHPPDTAPFYEQIKSKLQLDFNKNQLVEKLRRLKKKYRNVMSKISSGKEVFFKSPHD
QSTFEISRKIWNQTGKIIGFEDNNVMDFEETNNHHNTTNGNYSTFNSPSSNPTLELDSENGIEKKLTMSSSSVSR
KRSRSRIGKIEEDNKPVITPSDGPIASNVNLNEAAAVGIGGNLGGLIEETVKNCVSPVIKEMMNGTTSMMMAA
MGGGFPGLGGGGGGGGGHGFGSLSPMFTRPFNFGFGVEGGGNKAVADERWRKQQILELEVYSRRLELVQE
QIRTTLNELKTMPSGI
>AT3G04930.2
MTSDHRDALFSLELESPDPDEGGVADGGESDTDEDLRDNDDVVMPGTNEAEAEDDDPEEEDLNSPSTSSLPM
VSTISATAVVSGTPTATSSTGAVTVALPAGSAVPVSVIPVDSDPKWHRMTEIVHQRPPIDDSRRLFQRLWTDEDE
IELLRGFLDYMTMHRGSSSHPPDTAPFYEQIKSKLQLDFNKNQLVEKLRRLKKKYRNVMSKISSGKEVFFKSPHD
QSTFEISRKIWNQTGKIIGFEDNNVMDFEETNNHHNTTNGNYSTFNSPSSNPTLELDSENGIEKKLTMSSSSVSR
KRSRSRIGKIEEDNKPVITPSDGPIASNVNLNEAAAVGIGGNLGGLIEETVKNCVSPVIKEMMNGTTSMMMAA
MGGGFPGLGGGGGGGGGGGFVYNLLY
>AT4G00130.1
MMRLRRLLERMLRRCCFRDCSVRLTKSLCFKKLIEFDATKNQIVTKLQRLKKKFNNAVKNARKKGQTEDEVEYAK
ESEKKRFDLSIMIWGSNGVLVAGKSSKKKVAPKEMKPEETDAKVVNEGLSIGREMVPFGSSCGLDESKLTTGWE
NVEDGAEKREVEEKWKKFKDKLFELLYERSVLMNKTEAMMFKAES
>AT4G00232.1
MDKANTNRSKVCGGSGEAKLTGKKRKNVSAKQSKKDAKKENSQMLKWSSKDEVLVLQGMLDFKSVTGKNPV
DDINGAYEFVVHEYISTVIDEDFIEKMKSLKKKLKKKQRIYDKDPSSSEPLYQKSSEWLKMIWGYDVESALEKPRK
SKRIIKL
>AT4G00238.1
MASLENPAIDSSSEFESSSEEISSSKESKPKEAPVTVPSTKTLNSPSAAVAVSDDSESEKQSFVLTRRKKKEGAAESP
AVKSGKKEGAAESPAVKSGNNEGATESPAVKSGKKRASEGTTSRDMHVKRIKKEGDNKKGHAQRVWSEEDEIS
LLQAMIDFKAETGTSPWDHKDRFFDIAKKSISFDVSKVQFFDKIRSFKRKYFGDRKVRSVESDHDKKCLGLAVSF
WGSDGVSLETPVKKKVKDESVLVKANSKEKNVKPLVKEDEQVVILGEDSEWFEESFLVPVIASLGLDEYSVKKKW
SKVSVETKKRIQEKMKLVDAKKCELLLANMDVLKEVTSVLTQTN
>AT4G00250.1
MAPLESPATASSSEVESSSEEIFKSSSEESKPKDPVTVPSSKTLKSPSAAVNSKTDSSDDSEKQSFVLTRRKKKEGAA
ESPAVKSGKKRAGEGSTSRDMHVKRVKKEDDNKKANPQRVWSEEDEISLLQAVIDFKAETGTSPWDHKNAFF
DIAKKSISFDVSHVQFFDKIRRLKNKYFVNRKNKSGESNHDKKCLGLAVLIWGSDGMNVESPVKKDESILVKGKA
NSKEKKVEKPLVIEDEQVILGADSEWFEESFLVPVIANLGLDEYSVKKKWSKVSLETKKKIQEKMKVVDAKKCELL
LAEMDVLKDVTSVLAQTN
>AT4G00270.1
MVTPKQIDFSSCVGADNSNGTLSHRRSPRNIPSSKRAASVAEEETMKKKMKMKKKKKKLDPPLIVRIWNEEDE
LSILKGLVDYRAKTGFNPKIDWDAFCSFLGSSIVERFSKDQVLSKIRKLKRRFHVHSEKINQGNDPKFTRSSDSEAF
GFSSMIWGQGDDDGMDKEHEVNGNGAAENRTNESGEEMLKEHEEEVANTELLNENGAAKTTENGTSSGKE

RHDEDNDDDDELCAVQDAFEAVMSQGLSGYQKKLQLEKLMNLGNGKRRELSDEWKALCVEETRFNIKKLRFS
AKLAEAANDS
>AT4G00390.1
MTKKLDPPTAPSSDEDDVETSEDDSSSSEEDEPIKSLPATTAAAPAKSTAVSAATPAKSTSVSAAAPSKSTAVSAAA
DSDSGSESETDSDSESTDPPKSGSGKTIASKKKDDPSSSTATLALPAVKSGAKRAASEAATTSTKRVKKDEESVKKP
ALFQRLWSDDDEISMLQGMIDYHADTGKSPSADTNAFYEFQKKSISFEVSKSQFSDKVRSLRKKYRAKEGKDEP
RFVKAHDKKAFELSKFIWGPKGIALDSNAKSNGVSKKSASKTKEKLDSVKQDLAFVGVSSTNGDDWFEKSSLAR
MIAGSGIDEYYVRQKWSSFTLETKKIVEEKFQLMQAKELEAMLDKSVRLTDLTSYFVDASKN
>AT4G00610.1
MVSVQNLKTDQLLNFLFKNPTKFLSRFSPMAKNKTLVTPSTVKKSSDVASTSKKLSGVASPAKKPSGVTSPVKKP
LEAVASTSSEEEEEDEPSSDSESGSESESDTEAEPMTLAAAAPSSSNEKRQSEGKPEEERAKTETETGKKPLLFQRL
WTDEDEIVFLQGMIKFAKDTGKNVSEDMNGFFEKLKDSISFEVKTDQFVNKIRSMKRKYIENKKTTTEHDKKCY
ELAEIIWVSDGDATALVKPKKKKLKVDEESDWFERSFVDGAFKELGPGVDEETWKKNWSLVPVKKRKRIEEKVK
SMQADELKTTWQKIDVVHEMRSLMAKYV
>AT4G01260.1
MAPKQLKKIENPVVSSSEEEESASSGESATSGEESDSSADSPVKESSKKPVVVSKPSGSKTTTKPESSTAAKRSFEK
TDEMSKKKSKNSMGEEDVKKKDETLKKNLFVRLFTEEDEAILLQGFLDFATKKENPSDHIDDFYESIKNSISFDVT
KPQLVTKIGNLKKKFNGRVSKGLKKGKNEEVMVFSKASDQNCFDLSRKIWGSNGVLYSKSKNMRQVQLGGSV
KVDEDDQEPQKHRFVISTLSSGQELVSYLKVENPNSLGVDDTKWSAKLDKIKDGKQKRKMEKNLKKIQAKEEEL
SMMRSEFVAAVTNVLSKQDNASYSCK
>AT4G25210.1
MAPKKAEEVVESPPVSSEEEESGSSGEESESSAEVPKKVESSQKPESDSEGESESESSSGPEPESEPAKTIKLKPVG
TKPIPETSGSAATVPESSTAKRPLKEAAPEAIKKQKTSDTEHVKKPITNDEVKKISSEDAKKMFQRLFSETDEIALLQ
GIIDFTSTKGDPYEDIDAFCIYVKKLIDFDATKNQIVTKLQRLKKKFNNAVKNSLKKGKTEDDIEFAKDLEQKGFELS
RKIWGSNGVLVTGKSSRKKVGGTPAPKEMKLVAHSTPKKQQEEAKKPERTEAKVVNTGLSIGKEIASFLNADNG
SSCGLDESTLTAVWAKVADGAEKREVEEKWKKLKAKQFELCLQRSGLVNETAKMIFKAYES
>AT5G14280.1
MATPTELGFSSPGGGGDDSDDNPPQKRTSKRTASETATEEETKKKKKKKTKHNTKMASPPSNRIWNEEDELSIL
KGLVDFRAKTGLESKIDWDAFYCYVKGSIHVKVSKCQLMSKTRKLKKKFLDQMEKIDQGNDPHFTRSSETEAFG
YSMMIWRKIDAEYTNGVMDKAHQSESGEEVFEEDEEVALIDKGAAKSGKSPHEAVVVVDKITTKKNGTAGKES
DDDDDDVLCAVRDAFETTMMSQGLSDYQKKLQLEKLMNLGTGKRRELSNEWKALCVEELKLNINKLRFSAKL
AEAANDDYSSSSSSSIVSWVEIPISMSINIGEISIPDLPIFFSMFLTIYLIAYFIVFRNWKPQIRPEASSCLISIFHGSPA
VFLATRAVFSSSERSFASANTAAQNTVLDFSVAYFLTDLFHYIVFNPNDVLFIGHHVATLFVFLTCRFLVFHGACAIL
GLLILAEVTSACQNAWTLAGARKNDPESRLAVKVYDLLSPPFYAFYSIVRGVLGPLFFGKMVAFYARGGAHGVIP
NWLWISWAIVVGIAITVSILWIWNLWIELFSERKANKIRVDKKIR
>AT5G28040.1
MASDQRDTDFSAESPDLEEDGGGGGGGRGGGETESDEDVVIPEPNEAEDDDHDPDPDPEYEDLNSPSMISR
APATKSSSGTVTVALPAGSAVPVASIPSDSDQKWHRMTEIVHQRPPIDDSRRLFQRLWTDEDEIELLRGFLDYIT
NHRGNSSHPPDTAPFYEQIKSKLQLEFNKNQLVEKLRRLKKKYRNVMSKFSSGKEVFFKSPHDQATFDISRKIWN
QTGKIIGFEDNNVMDLEETNHVNNANGSSGFNVSVIGNANVDVDSENGLEKKVTISRKRSRSRIGKIDEDKPVL
APCDGVIPNAVNLNENVAVGCDFGDGRNLGVLIEETVKNCVSPMIKEMMNSTTGMMMAATGGFPGGGAH
ALGVLSPMLMPSMNLGFGGKGVGDERWRRQQILELEVYSRRLELVQEQIRATVNELKTMPNGG
>AT5G41765.1
MDKANTNRSKKEVCGGSGEAKPTGKKRKNERTLTNKNVNAKKSMMDFKALTRHNPSDDMTGAYNFLHEYIS

VDVYSYEFVEKMKSLKKKLIEKMGINAKDLSSSIGSELLKLIWRYDVKSVVEYNLLKIEQANFTSHPQISFNNSEPK
KTNL

Tomato (*Solanum lycopersicum*)

>SlGeBP1
MDSVPNRHHQASYDNLDYEVDMDVEGDEDSTSSPSIAGEVTIAVAGVSTGDSGAGMMNPGKRPRIDDFAIP
VILPGAVKKPSAGFDESRKLFQRLWTDEDEIELLRGFLDYTTQRGLNTSSPQHHYDTTAFYEQIKSKFQLDFNKN
QLVEKLRRLKKKYRTVVSKMGSGKDFVFKSAHDQVTFDISNRIWSTGGSYARSSPGGGSVGFSAPPLEDGGVGL
GLEEDLDVNPNPNPDSIVLYHSPKFNLNSNPNGIAIKTPRSHKRSPIPLEAVKVEHQPFQPPGIRINVQMPATNS
NPVAAVPAAPPVSGVAAPQVSGVATPPVGGTAAPLVAGVAAPAPTTGAASVPNLIEETVRNCVSPIFKELLNNV
ANLNGSARGFGFGFGGIGMSPMGLGMSPMGLGFGGGAMSMEMMRDEKWRKQQMLELEVYSKRLELVQ
DQVKAQLEELRSMGSNT

>SlGeBP2
MENGVLQQGSIILTNSIVDSEIMQRRGKLMPRGGGGGSEKMIKKKSEKVGAWSKEDEITILKGLIKLKTEKGKIR
FDYVQLYDSIKQSLAHKSATPLHLQKKIKCLREKYKNNFNKSTRTWSIPHEEELFYLSDKIWGKDDHQQLKIPSSSL
MNQQQLTIPSSSLMNQQHLTLPSSSLMNQQQLTLPSSSLMNQQQLTLPSSSLMNQQFTISDTTFVGDLGLALT
TMSRNQSNQMQVALQQFKLVSQSLSIRKSYANQILHAHITLKEEGLSEQKIARSYFDTKIQHAQLVSDAYNASH
GFGGSLVNIVSDCVLKEDATKVMNEGSCPPATIGCRKPINGKPSGTSLKSVREMCHSQERKKKKKRKIENPEKHR
DSSIQSQLEMENQSAVAYAKINYESAKSSDIHTLSDGLTVEVMVKGKADGKVASLGKQIKIHFIAKLRDTGCIVGS
TIGAAPHQFCLGYEKVLKGLNIGIEGMHVGEKRRLTIPPSLGPGRKAKPPIMPDSWLLYEVELVDICE

>SlGeBP3
MTENSTTGAANCSVEFVREEEEIVSNKRKNDDIDVTRFLYNDTPSDGYYQEKTPSPKPKRKMNLKSKPPLFSKV
WSDEDEVSLLKGIIKFKEQTACEITQCMTEFRAFILPSLTLQPTRVQLREKIRRLKKRYEKTLATGNSSNTDVHQLEL
FQLCRIIWTQPPNSLLLKEKREDGLQVKEKHNNPELEKQNIQVNEKKHNIPQKGQPEKQERKHNMRPIQQHX

>SlGeBP4
MTENSTTGAANCSVEFVREEEEVVSNKRKNDDIDVTRFLYNDTPSDGYYQEKTPSPKPKRMMNLKSKPPLFSK
VWCDEDEISLLKGIIKFKEQTACEITQCMTEFCAFILPSLTLQPTRVQLREKIRRLKKSMKRLLPQEIRLIQTCTNSN
CSSYVT

>SlGeBP5
MASVEDQAVVYNDDDLDDEEDDEESGHPQNEAVVDEDDDVDVDEEEEEEDDSTSSPPVPVANHSVAGEVTI
AVVGVPATDSLCIEPKRERINDFGVTPATMVVEEKKPLVLDDSRKLFQRLWMDEDEIELLQGFLEYTTQRGGINS
SSHHHHDTGAFYDQIKNKLQLDFNKNQLVEKLRRLKKKYRTVMSKMGSGKEFVFKSPHDQATFEISRKIWSNA
GPIVVRSSGPPPPPPADDGGFDDEDSHLNLNHANFIDHTPNFNLNPNGFDVKTSKSKKRPRGGEEKPGYIQQY
YHQSSGGGTNVILPPTPVSMAAPQATATATAAASIPSLIEETVRSCLSPIFKELFNNLNGSRYSGFGMSLGPSPLV
GFGNSLMSCDINMKTDEKWRKQQILELEVYSKRLELVQDQIKAQLEELRSMNS

>SlGeBP6
MDSTLPLSQSTQITQPILPSSPNKLPIKRKATDDPFTGAGAGDANGGVGEMGSDPPFKFHRIWTEPDEIRFLQG
LLDGSSENLFFPRDLNVFYTRFSNTMSQPYTKSQLSEKLRRLRKKFRVISSRLSRGLDRSLLSPHDRALYDLSKQLW
HPDFSDTSPFNAEKSKKSNLVGVKVSFLPNIYDPNRYGIVPYQDENGSNCNGVSVNEEVEQGEEEKENDGNIEF
DDEDGKLSEVNVELDTGEIGDERVEFSRVNGPVRVGIEFGVGDTAAKVVMDVFDECLKDFRNGERSNLGGVM
KEASSNEFEERWKEQRVAELNVLARRMRLVLEHSLQSL

>SlGeBP7
MAPKTKSRLVDQPPSASSSEEQELVEECQEEEEQQQQSREETEEEEEPPVVKRSITQKPVKTAQKLQFSSESDSE
NGSGSESKKSDHSPPSPFVSDFTIKPIVSAPSKSAGKRPQEAQKEKGRKKPKIAEEEDKKSAATPRSLWSDDDQL
ALLKGIAEYKTVKGMEPNADMTAFHEFIRGKLQVEVSKSQLSEKIKRLKKKFFTNAKGDEEPVFMKGQDFLVFQ

HSKRIWGAPGTSSGVKEIVTNSTNGKAKKTVEAKKSSEPKKSGKVSKHKDDEEHKEEEKQVAVKEVVKEDIVKG
DQQDFQSEYPRLAASFESMSGMFTIYPNGTSFLKEKMSLIAPDKAKVLEEKWKKLEDDEAALMVKRLDLIAEHY
RLVVDAMRGN
>SlGeBP8
MALKIKSRLVDQPPSASSSEDQELVEESHEEEQQQSGEGEGEEESGEETEEPKTAHPVVKRFLTQKLQLSSESGSE
NGSGSESEAESGHSLPSPSASDFTVKPSVPAKAAAPSKSVAKRPQKAQKEKGRKKPKIAEEEEKKSAATPRFLWS
DDDQLALLKGIVEYKEVEGMEPNADMSAFHEFIKGKLQVEVSKSQLSEKLKRLKKKFLTNDDEKHKEVEEQVAV
KEVVREDIVKDDHQDFQSEYPRLAASFESMAGMSAMYPNGSSFLKEKLSLIATDKAKELEEKWKKLEDDEAAL
MVKRLDFIAEHYKLVVDAMRGN
>SlGeBP9
MAPKTKSRLVDQPPSASSSEEQELVEESQEEEEQQSREEEGEEESGEETEEDEEPKTSHPVVKRPISQKLVQTPQ
KPQFSSGSENGSGSDSEAESGSSLPSPSASAFTVKPNVAAKAAASSKATPKRPQEAQKEKGRKKPKVAEEEEKKS
AATPRSLWSDDDQLALLKGILEYKTVKGMEPSADMSAFHEFIRGKLQAEVSKSQISDKVRRLRKKFLTNVKDGEE
PVFTKSQDFLVFEHSKRIWGAPGAINGGVKDNVNNSSNGKAKKSVEVRKISEPKRSAKVSKPKDDEKQKEEEKQ
IAVKEVVKEDIVKGDQLDFQSKYPRLAASLEAMSGLSKMYPNGTSLLKEKMSLIATDKAKVLEEKWKILEDDEAD
LMVKRLDFIAEHYRLVVDAMRGN
>SlGeBP10
MLHENDRKRLRSDEKEESDETGEGKTEELDPNEDEKERRRRRAVKKGKQSMSGRQSDADYYDYDYDYNQSLAI
LRSMWDYKSKTGDFPYPPSIELLNFIMYSIPNLTISREKLVKKITTFKNNYNDAWELDGDHPGLDRPVDREIFNLS
MRLWGDH
>SlGeBP11
MLPAIGRTDEEEEGQNGNRNSEQRETEEAGGNQLGETDEDRNDDYEEEEDMENLGSGNNVNHQNSEENGS
GNQESYDENHTSWLVVVEDDEDEEEERRRAAEKGKQPMSDGGGISITELHDEDEDHHLDLAILKAMCDYECII
RNSPQLSSFNNRLVYVKDSFPDLKMSREALEEKIIALCKNFNDVREQLGDDPRMDRPIDREIFNLSMIIWGTKVI
PR

## Pepper (*Capsicum annuum*)

>CA00g34580
MIAKKKKTTERIHIGGDGNVIHQNSKQNGSGNQESEGGDEGEEDTENDDNQEENGDKEEDEEERRRRATEKG
KKVSKHGELEFYISYDANHLDVAILTSILDFNSHCGDIPHPPSLDLINHIMNAIPDLQISREELREKIIVYKENFNKVR
EVQGDNPDMDRPIDDEFFDLCMLLWGNQPDDDDDDWIWK
>CA00g89360
MIIKGKLHADVSKSQLSDKIRMLKKKFMTSVEYGEVPGFLLVFEYSNQIWGAPSNNVVKENVSNSANGKAKKA
VTVVVKKSAEHK
>CA02g03800
MAARNIFKSLKAKRPHGDSDKEWTPSPEDSQTYSPEEVIMESSNVNKSGFQRAWSEEEEIAILQGYLNYSFLKN
SEPSSNYGAFLTFVKDDLQTETNKTQLQHKLTRLKRKYKRNIGKRKFSKSHDEKLFKLSEKIWGEVDKVRNKVDK
TSGKMIVDASRSEMSWDFVLNGGTSADLEDWFRRNPRQLISEKERNEMLEKSQSVKVAKARQYLLEIQVMEE
QAKMATDAIQAILS
>CA02g06780
SKAITHVKDDNALVKTQSKPFQRVWTDKDEKILLEAIGSYRAIHGKEPTSNNLFNIIKGKISFEPTLVQVGNKIRSL
KRIKRKTRQRIVEDTPAEKSLFCSSQKVLGVNKMCSTTAQSSKGKKVLNDAEIARIVMTLFQGYEGDEVMQIETS
TRKVSQLIASGLSTKWKDLIKAQANQYLIGLNNRKSKVELFLEMMEVEAR
>CA02g07070
SDTPCLVQSEDQMLRRAKQPPNKNKKKRCRGNDYDDVIKEERKGKHVHLMPSLDDNYGDSKEPEKKSTYANT

SMPDTDCGLGLCDQSGVLPEGLIKEKTGSSLSSSWSKEDEITILKGLVRLKTENEIKSKKIDYIALYESIKQFLHRESD
IEDLKKKVKCLREKYKKNLKKAKTPSVPHEEELFHLSDKFWGDGDKDHEINQQITCHSSAPSCSSIRPYKSLDLEA
SDKIFVGDLGLALATINRNQSNPVHVALEQYKLVSQSLRIRKCYGNQILEARITPKMV
>CA04g06080
KSFQKVWIDKDEKTLLEAIESYRAIHGKESNNNKLFNIIKGKISFKPTIVQVRNKIKSLKKIKSKTRVRTIEDTPAEKS
FFCSSQKVWSVNRMCSTTAQSSKRKKVLKDTKISNIMMTLFQGNEGDEVMQIEMSRRKVGQLIDSGLPTKWK
DLINDQANQYLIGLNNRKSKVELFLEMMETEAR
>CA04g15740
MKKKNPAKRWKFLRRXXXXXXXXXXXXXXXXXXXXXXXXXXXXXXXXXAKKPPPPQDSSSSEEEESESESEPDPD
PVTQKPNLLVKPIASKPMDVPKKSSPKPRSKSDASAKLVSPKKSSSTVGAKRAAGEGEAKESKKSKKKQEKEVET
PVKKPVNEDVKRQLFQRLWSEDDEVAILKGMSDYRSKKKADPVADLGAFHESIKKSLHVDVSKAQLQDKIRRLK
KKYMNNAGKEKGKERVLTKAHEQKVYELSKKIWGKEKSDKGDQVEVVKVDNVAASKGRQDGGVKDNGVLFE
VKEEKPSAEVEKDVEVKRNLNLIPCVENVAGLEKWLEENSGVLSVEKRNAMKEKWEALKAAEADLCLRRVKLIA
EQMELVLEAVRDSGTET
>CA05g13060
MDSTLPILPSSKLPIKRKTPSSPTAVAEPAVDTEPGSDPKNPPFKFHRIWTEPDEIRFLQGLVAGSSDNLVYPRDLN
VFYTRFSNNMSQPYTKSQLSEKLRRLRKKFRVVSSRLVKGFDISLLSPHDQALYDLAKQLWHPDFAYTSPFNSDK
SNKSGLVGVKVRFSGNVEQPGQDGITRNGVEFEGEEELREVNVELDRGKMGGKRVEVSRLDGPVRVGVVCGI
GIGDIAAKVVMDVFDECLNEFRNGVGGERSDVGSFGSGVVGEKSNLGGFGNGVVGERSSLGGFGNGVVGER
SSFGDFGSGVVGERSNVDGFESGVVEERSRIGDFTNGVVGESSNLDGVRNGFVGERSNLGGFRNAVVGERSN
VDDLTNGVAGERSNLGDFTNGVVGERSNVGDFTNGVVGEGSNLGGLLKEASSGKFEERWREQRVAELDVLAR
RMRLVLEHSLQRQ
>CA07g12470
MAPKAKSRLVDQPPSASSSEEEQVEESQEEEEQEEQSEEGQEEESGEETEEDEPPKKPITQKSLQTQPKPVHSSS
ESGTENGSGSESQTDSGHSGQSLPSPSASDFTVKPSAPVKAAVPSKEPAKRPPPQEQAQKESKDSRKKKPKIAEE
EEKKASATPRSLWSEDDQVAILKGMVEYKQEKGTEPNADMSAFYEFIRGKLQADVSKSQLSDKIRRLKKKFMTS
VKDGEQPGFSKTQDWLVYEYSNQIWGAPSNNIVKENVSSSANDKAKKAAADAVKKIAEPKKSGKVSTLNKAK
DNEKRKDEEKEKKVAAIEVVKEDIVNDDKQDFQSKYPRLAASFESLGGMSMIYPNGTSWLKEKMSSIASDKAE
VLEEKWKKLDDAEAALILKRLDLIREHCGLVVDGTRGN
>CA08g10840
MASHHHPGYNDDNLDYDVDVDGDDESNSSPSVVAGEVTIAVAGVSTSDSHAPLIQAERKRPRIDDFAIPVILPG
AVAKKPAAVDESRKLFQRLWTDDDEVELLKGFLEYTTQRVLNNSSAQQHHYDTTAFYDSIKSKFQLDFNKNQLV
EKLRRLKKKFRTLRSKMGSGKEFAFKSAHEQVTFEISSKIWGNGGSYARNSPGRGGPIGFPAQHPLEDGGLDDE
DAHSNPNPNSDAIVIYNSPKFNLNSNPNPNGPNGIDMKTRSQKRAPQVPAEVLKAEQQVFQPPGFSPNVPM
PSTNVNPMGTTPMVGVAATAPVAAAPVVGVADTAPVAASVPSLIEETVRNCVSPIFKELLNNVANLNRPARGF
GFGFGFGGMEMSPIPLGFGGGAMSMEMMKDEKWRKQQMLELEVYSKRLELVQDQVKAQLEELRSMGS
>CA10g00710
MAADEGETKESKKSKKKQEKEVETPVKTKATEHVKLQFFRDYRVQLQNKIRRLKKYMNNAGKERGKERVLTKA
HEQKEYELSKKIWDKEKSDKWVQVEVVKVDNVAVSMGLKKGGVKDNGVLFEVNEEK
>CA10g07350
MASVEEDQHAVHNDDDLEDDEDDEEGGCPPQNEVVVDEDDDVDVDDDEDSTSTPPVPVSNHFGSREVTIA
VVGEPKRQRINEFPVATTTVVEEKKPLAFDDSRKLFQRLWTDEDEIELLQGFLEYTTQRGGINSSSHHHDTTAFY
DQIKSRLQLDFNKNQLVEKLRRLKKKYRNVMSKMGSGKEFVFKSSHDQATFDISCKIWSNTAPIVIRSSAPPPQE
DGGFDDVDSHLNLNANFIDHSPNINLNPNPDGIDVKTPKSRKRFRGGAVQAEEKPGFIQQYYQPSGGGTNVIL

PMSSSPALMPTPVPMAAPPLTATAASIPGLIEETVRSCLSPILKELLNNVTNLNGSRSFGFGIALGPTPLGFGSSAV
SSDMKLDEKWRKQQMLELEVYLRRLELVQDQIKAQLEELRSMTS

Soybean (*Glycine max*)

>Glyma.03G245200.1.p
MASEQHDAVFREEDMDDDDDESQEDEEYEEDDDEENVPSPSTALAVTVAVPGSAVSNGGGGSPISKPTATATT
ATIVLADSSDPKRRRLELIEEKKPPPPLDDSRRLFQRLWTDEDEIELLQGFLDYTSQRGSSHHNDTALFYDQIKSKL
QLDFNKNQLVEKIRRLKKKYRNVLNKICSGKEFSFKSAHDQATFEISRKIWSNVTPVGDNSLDDDEINPSRSPNP
NLNFSPVILKNETIFRNSTEKKTPKRSRPRSAVKIEPNDGSASNRDHNCISNTTPTATAAATNTTAAAAAGTNNN
NCNSGYGNNIPSLIEETVKSCLSPVLKELMAGAMGGGAFGGRGFSLNLNPMPFMNLSFGGGEMVDEKWRK
QQILELEVYSKRLELVQDQIKAAMEELRSHGGGGL

>Glyma.05G088300.1.p
MMSSDRITLKIGPPLLFSFPGEVRGFYLVSAFLHQKQLPPPIEASSDEEQRPSSKQHTEEGVSSSEEASSQEEDDD
DQPPTLPLASANPHPKPSSSDSDTDFEPTKVKPKPTDQAQKPQPSPAPPKWGSKRPAQNNAPATDPKRAKKKL
TNSSSAAAAHETEEKSGGGQAKLSQRLFSKEDELAILKGMAEFISKTGQDPYKYADAFQNFVKNSLRVEASSNQI
KEKIRRLKKKFETKAQRAKKWEDPEFSKFHDRTVFELSKKVWGEGANGLVEKPKPNNGKRKTAKTPKKDATSR
NVVAKSETTTLLESMELEECGNVNLLYREVSGFKELNEDEMKRGLALIGESKRKELEGKWRKLRLAEMELVANRS
LLIGEQIKLIFEALQ

>Glyma.05G088300.2.p
MMSSDRITLKIGPPLLFSFPGEVRGFYLVSAFLHQKQLPPPIEASSDEEQRPSSKQHTEEGVSSSEEASSQEEDDD
DQPPTLPLASANPHPKPSSSDSDTDFEPTKVKPKPTDQAQKPQPSPAPPKWGSKRPAQNNAPATDPKRAKKKL
TNSSSAAAAHETEEKSGGGQAKLSQRLFSKEDELAILKGMAEFISKTGQDPYKYADAFQNFVKNSLRVEASSNQI
KEKIRRLKKKFETKAQRAKKWEDPEFSKFHDRTVFELSKKVWGEGANGLVEKPKPNNGKRKTAKTPKKDATSR
NVVAKSETTTLLESMELEECGNVNLLYREVSGFKELNEDEMKRGLALIGESKRKELEGKWRKLRLAEMELVANRS
LLIGEQIKLIFEALQ

>Glyma.10G160000.1.p
MESDLNDAVFPEEDLDDDDDDETPEDEEDYDDETEPPPFVLAVVAVAPPASTASETLDTTLIPISAVADSSLKPLH
PELIEEKKALDDSRRLFQRLWTDEDEIGLLQGFLDYTAQRGSSHHSDTALFYDQIKSKLQLGFNKNQLVEKLRRLK
KKYRNVVTKISSGKDVSFKSPHDKATFEISRRIWSNTAPISGPVEDDDEINPNPNFGNSAKTPISRKRSRPQKREL
NDGSTLNRDNNCIGNNNNNSNSNNNNNENCNGRHNLQGLIEETVKSCVSPVLKELVCGTGGMELGRGFGVG
GGLGVGGGLALNSLQTQMTMPMTLLNLRIGETTMDEKWRKQQILELEVYSKRLELVQDEIKVALEELRSAGGV

>Glyma.10G160100.1.p
MHNCHKRTNGYLDTLICEKRKCDISSTMLSRLVSWILSPSSSEEEEEILDIIKDNDINHENDQKFNVEDDKNDHFL
NSCDVDDTIPIALAVPNASPAVTVAFPANDERNTVPTTATVATIVTRSKGQRNAKYSGMVRQYQRLRTKEDEME
LLKGYLDYVKQHRKETTTLLYVVVSCMITNQLVEKLRRLKRKHKLALEKGKDKEVPFRNPQEQAIFEISHKIWAND
TDNIIVQDALDGDESGHTPESHDHVGNVKVKIEQVDNSDEIGNRVPKRLRLDDADDMNRTNDQNNGDSIQG
FIEDTMRSCFSPLLKEVLDEAQEESLPELEAIPMPLCSGEVDHEQWRKRRILELEVYVKRLELLQNQIKSRLEELRS
S

>Glyma.13G251000.1.p
MAQKQKLRPSPLDEPPTASSSDSEEEEPQQQQPSSQKNKEEEDEEVSSGEEEEEDEEEEEAASSEEEEEDEDLPP
PPVSKNPPPPPANPQPQHSSSESETESGSETESEPDPTPVKVKPLASKPMDQAQKPKAQPSPAPPPKLTLKRPA
ENNNNNARVADSKRAKKKATESSSAANSAAAAASDDEMEEDGKKSGDNSKKFQRLWSEEDELAIVKGVVEFT
SKTGLDPLKFPNTNAFHDFVKKSLHVEVSCNQLKEKVRRLKKKFETQAGKGKNGEAPKFSKPHDQKFFELSKKV
WGREVTAGANGGPVEKPKSNGSAVKSPKKKESGSRNVASAKKPKPESKPEPVPVLSLEYKDSEKMQINQKPDG
GDASLFLRELARSKEGASICKLDEDDVKRGLELIGESKRAELRGKWKKLHLAEMELFANRSELIGEQTKLILEALQA

SDH
>Glyma.15G063300.1.p
MAQKQKLRPSPLDEPPTASSSDSEEEEEQQQQQPSSQQHEEEEEEVSSGEEEEASSEEEEDENLPPPPISKNPPP
PPPSNPQPQPTSSESETESGSETESEPHPTPVKVKPLASKPMDQAQKPKAQPSPAPPPPKSASKRPAENNNNN
ARVADPKRAKKKATESSSAAAISDDEMEEDGKKSGDNSKKFQRLWSEEDELAILKGVVEFTSKTGLDPLKFPNA
NAFHDFMKKSLHVEFSSNQLKEKLRRLKKKFETQAGKGKNGDAPKFSKPHDQKFFELSKKAWGSEDGGVANG
SVEKPKSNGNAAKSPNPKKKESGSRNVASAKKPKPETNPEPAPVPSLEFKESERMEIDQKPDGGDACLFLRELV
RYKEGANVSRLDEDDVKRGLELIEESKRAELRGKWKKLHHAEMELFANRSELIGEQTKLILEALRSSNH
>Glyma.19G242600.1.p
MASQQHDAVFREEDMDDDDDESQEDGDYEEEDDDVLADDEENEPSPSTALAVTVAVPGSSVSNGGAAPIST
PTATTIVVADSSDPKRRRLEPVEEKKPPPTPDDSRRLFQRLWTDEDEIELLQGFLDYTSQRGSSHHNDTALFYDQI
KSKLQLDFNKNQLVEKIRRLKKKYRNVLNKIGSGKEFSFKSAHDQATFEISRKIWSNVTPIGDNSLDDDEINPNRS
PNPNLNFSPIILKNEMIFRNPAEKKTPKRSRPRSAVKIEPNDGSASNRDHDCISNATPTATATAAATNTTPTAATT
NDNCNSGYGNNIPSMIEETVRSCLSPVLKELMAGAMGGGAFGARGFSLNLNPMPLMNWSFGGGEMVDEK
WRKQQILELEVYSKRLELVQDQIKAAMEELRSHGGG
>Glyma.20G228300.1.p
MLSPLVSWILSPSSSSSSSEEEEEEILDIITDNDINHENDQKLNVEDDECDVDNTIPVALAVPNASPAVTVAFPAN
DERNTIPVTATATTVVTCSKGQRNAKYSGMVRQYQRLWTKEDEMELLKGYLDYVKRHRKETTTLQSVVASLYD
HVRPKLNVSFNKNQLVEKLRRLKRKHKLALDKDKDKEVPFRNPQEQAIFEISHKIWGIDTDNIIDQDALDGYESG
HTPESHDHVGNIKVKIEQLDNNDEIDNRVPKRLRLDDADDVNKTNDQNNGDSIQGFIEETMRSCFPPLLKEVL
HDAHEEPLPELEPIPMPLCPGEVDHEQWRKRRILELEVYVKRLELLQDQIKSRLEELRSN
>Glyma.20G228500.1.p
MESDLNDAVFPEEDLDDDDETPEDEEEEEDDDVLDDDETEPPPSVIAVAPPASETLDTALIPISSVADSSPKPLRT
ELIEEKKALDDSRRLFQRLWTDEDEIGLLQGFLEYTAQRGSSHHNDTALFYDQIKSKLQLGFNKNQLVEKLRRLKK
KYRNVLNKISSGKEVSFKSPHDRATFEISRRIWSNTAPITGPVEDDDEIITNPNFGNSAKMPISRKRSRPQKRELN
DGSTLNRDNNCNSNSNNNNNNENCNSRLNLQGLIEETVRSCVSPVLKELACGTGGMGLGRGFALNPLQMP
MPMSLMNLGIVGETAMDEKWRKQQILELEVYSKRLELVQNEIKVALEELRSAGGG
Rice (*Oryza sativa*)
>LOC_Os01g14720.1
MPCGYGVPIISTLDFTARRGTTFASHQYDTGPFYDEIRRRLSFDFTKSQLVEKLRRLKKKYRLCAARMASSPHAAA
AGFAFRTPHEGAIYDLARHIWPPALKRDGTASDDDDINPAAAAATAAVMTPVAMEDGFGGSAPTPTPTPRGR
GGRRVRRRMAQEQEAAALPSAPALTSTDGAHQEPLVAAMENTLPQIAQLPPVSETEPMPVIANGANEEAVRS
VLSRLLKEFITSFAVVGQTGPGMGLNMGFGGAGLNADIAGLGFGIAGLNPGVPGADRWRQHQILELEVYLKRI
ELVREQVTAALDELRSSEG
>LOC_Os01g32890.1
MRRRSPPPAAAQPPRPPTRAAESGEEGGDPGKDEVAAPPKGDSALPRSGSGFGSGNAAAPDGPAKSPMKPK
KRVTAPPSSDHGSRENLAAGSPSNEVPTPQGDDAAQEDDLPESPPKSKKNRKKKKALRAGDSGKVAAPDDP
AEPTRPQQDVGEVDGRLAESEQEVPPNAGKAKKKASAQARKKPSAKQHAAAQEEEDGDLMAEAEEEVAPW
QGDEEDGKGPLPQRKSKRVAALSGSICPPDPKRAKIVDAQKPGFGRKWNGNDEIMILEALVDQIRSGGNVPQE
PGHPLFHELVQRLEGRTFNHSDVRDKVRSLKRRYNDVVLSGLAITKDHDLQLHELSCEIWGRSVAHAGDDKQR
CLARDEQSSLARDEQKSFARDEEKSLARDEQSSLARDEQKSFARDEKSLARDEEKSLASDEQRSFDDMCKQFPL
LAKEIKVLMEGQPAIMELFPRLDGDQVVAIEKKLENLRWIDMKRKKKMAVKMAKIRKGLIYKLEGAAILADGN
MIH
>LOC_Os02g18660.1

MARKRRAPTPPPPPPPPPPEAESSADSSGEEEEEEEEEEEEERESPVAPPPQKPSGRGAASSDEEEEEEEDSDTDTY
AQGFQLRKVGGGDEEGEEVEGDSSESEPEPEPVKKESAKKAKAEAKKKRAAPEPAPSGKAKKAKPEKSSSAAAP
EPAPSSGKSKKAAKAEAAKAAAAEPAPSTGKVSKYNLAPEPSPSSKSGKALSRWTTDDEVKILEVLVAHFKSHGT
QLKVEGIIAAVGDSLERKSIKYSDMYEKVRRLKQRYEATAKKVEHGGDLPAKEDDLRMYQLSSEIWGKNAKDAG
NSSKNKKGQAKKDKVSGDSKEAAKEDKVDEAAIAVNEKGGTLAENKKGKTNKQKTGMETKVGSSKEAALAAS
PTKGKKKGSHKDKLDEEAKSGTAKVTSTIATDDDDGTLGGSKREKAGKEELDGDTHIVMPKEATTTAASDDGTL
VGSKKGKADNGKLDGDTHSVMPKEATAGTQNGGILTGGENHKEKVDKDANVPSIRREYAELQSLYPNLASFVN
GIEAQHPCGSTFKRAFEFISDDKACTLESKIKKQKIAEVRMQLRLADTKKEVANALLGLLD
>LOC_Os02g52950.1
MPSKRPSQSAMDAGAAPASPSPPRSSKKRSSRPKPRAGDAARHPAPNPSPPPAAAAPASSRSRERERKRRQRG
AFADPAAVTAPAAGGQHGGAVQKLWGDADEVALLAGAAAFRARAGHVPRLPDMGALFDSIRGSLSPHIDQA
KVYYKLKRLKGKYLHAAPGASAGPHERRVRDLCASVWGADLEPLAEGDDERAAAAAAAAAADQPRTVPDAA
AMLPVLTEMLDEYWKTDGRALSSVSLAKGLSLLGTEEARFIEGKWRRQLDSEIQTQMRRHDLAKEVYALLMDA
IKALGP
>LOC_Os02g53150.1
MAPKRPTPPPPPPPVASSEETASGSDSDESEEEEEEESPLAQPAPVVSNKGAESDSSGEEESEEEEEEDLVRSSATK
SRDPPQENREEDDSSDEEEDESSESEKAEAPPPPPPNPAPKQGAEGNGPKVSSPKRQAFHRIWSTEDEVRILEA
LAAHRREHGSLPQTDALIATLAGSLDNTGYGRKELQGKVSTLKRRYESTAKKKGDLPSKGHDRRLYDLSKSVWGS
EAAAAAVNGTTTAPREFGEMCELYPHLAEEVKLLEAAHPGLFKRDFGKLDDDKAHALDMKIKKQRIAEISVVLR
RGDLTKEVTKVLAELVE
>LOC_Os03g25430.1
MLPTVVDDPSAAAAAAVGAAASSSFPDADVYGNGDSDDIDFPSDPNHATTQAFSSAAAAAAAAGPGGGGG
AGSGSGGERRPLFQRLWTEEDEIVILRAFAEFTAQRGTAFASHQYDTDPFYEDMRRRLQLDFSKSQLVEKLRRLK
RKYRNCVDRLRATGQSFSFRSPHEQAIFEIARGIWRPTSDKHGRDADSDDDALPDAAAAVAVPAPANGEVRSP
SSTRAQQRRGRRRRTAAAAAATATAADASEPPQPYPPAPAPAPVKAEDALPHFFPQGAAVTATAHVHGVDPAS
AAASAAANAEGGILAPLFKEMVRAMLTAGMAPPSLEPPAPGIPMKGERWRQQRILELEVYLRRIDLLQDQVRT
ALEELKSTPPATQ
>LOC_Os03g50110.1
MASDQTLPVPVSVELPPAAPNPPDPTAPLLPHADDPSAPPAAAAAARKLPVKRRSPPPRPSSPSSSDPASSDPA
AKQQPQQPPPPFKFQRIWSESDELRFLQGLLGCGAQGLVFPRDLNVFYDRFSESMPSPYTRSQLSEKLRRLKNK
FRGMSARVARGLDPARLAPHDRDVLHLCSRLWDPANAATSPFAAAGDASSGNKRRRAAPRAGPLMPPDPSG
DSNSHDYNGGISAGTPGAFGDGHGGEEMMYLEQESGHFGYHGDVAIAADGSLDGIVKVQPETLPALPSIGDIA
VHSENGNGKAVVPRSNEHHMANAVLDIFEECLREVKANGITYGANVNGGSELARRWRAQRIDELDVLSRRLR
LLVEDAAAAGR
>LOC_Os06g10710.1
MSSSRRPPARAAATEAAADAALPLHPSSPRSKKRSSSSRRAAAGDRRPAARAPNPSLSPRGGGGAPSRKSERRR
RPRSLAMAVHGHASTSGGPGLVWNDADEVALLTAAVAFRARNGFAPRLPDMGALFESLRGSISSHIDQAKVYY
KLKRNKSKFLHAPPQATTTTPHDRRVRALSAELWGSELAPPAVEGDADAAEAADERDAEEGYIGGNLHVSVRL
PVVSEVLGDYWRKNGRVLSGVSLERGLALVGPEEGRMAEAKWKRQLEVETQTQGRRHDLAKEVCAMLIDAV
RGLGP
>LOC_Os07g44200.1
MLPAGGYPSAAAISFPDADVDDDSDDGDFTDAPLIDPSDPTFPNPASSSAAAAAMATPASAGGGSGSVGGVG
SSSGGGERRPLFQRLWTEEDEIVILRGFAEFTAARGTAFASHQYDTDPFYEDMRGRLQLGFSKSQLVEKLRRLKR
KYRNCVSRLRGSGSAFSFRSPHEQAIFEIARNIWRPTNKHGRDGDSDDEDATQVTPAPVPVNTSPNDHNANYS

DNGGLGTPQIAGHATSAGCFRCRQELLDASV
>LOC_Os08g36450.1
MAPKRPAAASGSASEASDCEAGGAGRRRSPSRSRSPSRSASPSRSRSKTPPHNAAVLSSTPASAAVDFVAASDS
DAGADADARLASPRRSRERSPRLHSDSDNSAAATAEAAEAAAAAAFDDGDDEGNATPPPRSRRSSRVEATGVK
PISSRPMDASRRPAAASSQSQRRSKRPRSSPTQHSPEQHKRPPRVWNPQDEVTILRALISYRAKNGALPGSSQD
TGKLHNMIRGQLSVKASTTQLSDKVRRLKHKYNLILTRVTKSGRDPDLPTEHDREVYELSKKVWGTKSGGAGA
GSGGGGGGGGGRVYENAEVVQSDEEQGSRDDSDEDMESGWDDRDHRNRRLKAIVVANGNGNAVTGGRS
VHGNGSGKGDVADKGKDMYPYLWEAVEELSKEHPSGTAFRKAFGVLDGSRARAMEEKLNRFRLSEIRQQLRR
MDLMKETIKMVLDALEGSDL
>LOC_Os09g01140.1
MAPTLPVVPAHGEDKKKKKKKKKPLKPSQEEEEALPLPLPPPDRKRKKASEPVNSPERAKKKKTATPHEPPSAKQ
QKRPLPFQRTWSPNDEVLILEAMAAHRQEHGKVPTAAELFPVLNGRLDRKRLTYKKLADKLRTFMRRHGRDA
KNGPPTQAHDRRLYDLSRNVWVSQTQPPNLSANANSNIAGGQPNQHDAMPTAGKAFDKMRDSYPNLTQAL
LLLVGTDLEKALTAIDETKAQALDLKVSNLKKELSEAVMESATIQSTESSKIPCFPSTKLQPEFGAEIEKNFQLEHLD
EMKGTQVKLARMEQEILELKQNFLAFQSQQMADSKQQHDKSSAKGIICESSESGLRSIVADNNILCNTLQKEM
VVQQKLSCGKTKEVTSKHRHPQKLVVFPF
>LOC_Os09g01470.1
MSSWWSWRSLFSSLANANGGGSNADASSGSGTSSSPPVHEAQQAAARRRSARTKKPPEEEAAGSQPQPKTR
PSPASKASKAKVLLLLGDGEPKKKPAPNPTPTQKRSNKRKRSWSRADELRILEAMANHANAHGGALPEASDLFA
ALASSLERGDADLPKLADKVHKLKRWYDNARLPQRCPTDDDDDTRRLFQLCGKVWGPPSTVLRTSPRQRHKV
VGVLVQGNGANPQPAAALKVKEKRVRRELSELYVLYPCLAQEVKAHANEYGELIGTAFQFIGDDEARCYDDRYR
KMLVDKLNMKKEHADVTKSLLCTLAGYIN
>LOC_Os09g27850.1
MAPKRPAEEADAAAAAAAAAGGSASEGSDAEASAEAARGHGSSPSPSKTPPPANPNPKSAAAPPSAVAAPAS
AAGSDSGAASDSPRAAGNPSGPRSIEVNSDSEDSALPLASDAYADQAAAAGAGAGADSDDGNTSPLPPPRPS
RAEAAAIKPISSRPMDPPPRRSAGGSEPRAKRPRSAAVASSAEHSKRPSRVWSQADELVILRGLITYRTKRGVLP
GSTQDIGKLHSYIRGQLSAKVSTTQLSDKVRRLKQKYQMLATRAKTGKEVFPTPHDHNIYQLAKKVWGTMSTA
GEGGGSGYDNADAGESEEEQYGRESDDDMESGRDNRHRKNQRSVPVTMANGNGTGIGAVNAIVRGRSEFE
KGKDVYPYLWETVEELSSQHPTGAVFKKAFELLEGSKAQVMEEKLRKFRLTEMRQQLRRMDLMKDTLSMVLD
ALEMAD
## Maize (*Zea mays*)
>Z.mays EF517601.1_FGP010
MADHDPRAAAAASSSAGGDEDDEGTDTDVSDSNLGNPQGQDPLPFPDAAWVPPHPLAPAHEPAGALPPPLP
QGPSAGASATTDDSRRLFQRLWTDEEELLILRGFLDFTARRGTTFASHQYDTGPFYEEIRRRLSFEFTKSQLIEKLR
RLKKKYRVCAARVAAQGDAFAFRSVHEGAIYDVARHIWRPAFRRGEGGGAADASDEDDINPVAAAAMEDGG
GGSASTPVPTARGRGGRCVRRRTAQELEAPALPATSALLLSDAAEDRHVVAVENLAPPPPLQVPTVSLTGATPSP
MPVCTGGAAEEAIKSIMSPLLKEFINSVAVGGQSGFGLGLGTGFGGIGGFDILGLGTGVPGPNPGMPSDDKWR
QQQILELEVYLKRIELVREQVTAALEDLRSSEG
>Z.mays GRMZM2G006871_P01
MPPPPTSAPHSLAADDFHTSDTAAAAAGAVVPCAASQPGTNGGKKPAFAFGRVWSEADEVRILEGLAAYAAA
HGAEPRRSQLHAALGGCGLDKSEFTVTEIYEKVRRLRTKYANRRSAGGVPVPAAAGGAGDGDEARKYELSRSI
WGDLPLKKVGASHATNASGNGNGNANANAAPGTRARRGIEELQGLYPSLASAVEGITDNESLRPVLKRAFQLI
SDERARQLDAKVKKQRDMEAQMAVKVTALRNEVLDTLTGSMDCFINDVKYEG
>Z.mays GRMZM2G008558_P01

MDEAASASAAAARLRASTPRSKKRTSRSKSRGRSPNPNPSSRRERAADHGSAAPSRKSDRKPNRSFPDSATLAT
AMASVPAASAPSSGGGRGSAGAVQKLWSEVDEVALLTGAAAFKDRTGIAPRLPDMGELFESIRGSLAPHLDQA
KVYYKLKRLKSKFQHSVLGDSSTAHEHRLRDLGAALWGAELTRPEENAVVTAEETTDPDEGFIDEDREGSVKLP
MVKEVLGEYWRLNGQTMSGVSLEKGLVLLGSQQASVVEVKWRRQLEADMRMQMRRHDLEKEVYGLLIDAI
KGLGP
>Z.mays GRMZM2G008558_P02
MPSKRPTPHAMDEAASASAAAARLRASTPRSKKRTSRSKSRGRSPNPNPSSRRERAADHGSAAPSRKSDRKPN
RSFPDSATLATAMASVPAASAPSSGGGRGSAGAVQKLWSEVDEVALLTGAAAFKDRTGIAPRLPDMGELFESIR
GSLAPHLDQAKVYYKLKRLKSKFQHSVLGDSSTAHEHRLRDLGAALWGAELTRPEENAVVTAEETTDPDEGFID
EDREGSVKLPMVKEVLGEYWRLNGQTMSGVSLEKGLVLLGSQQASVVEVKWRRQLEADMRMQMRRHDLE
KEVYGLLIDAIKGLGP
>Z.mays GRMZM2G026417_P01
MFPTVDDPSAAGAMVSASFPDADAYGNGDSDDLDFPVDPNPNPVFSSPAAPASAGAAGERRPLFQRLWTDE
DEIVILRAFAEFTAQRGTAFASHQYDTTPFYEDMRRRLNTGFTKNQLVEKIRRLKRKYRNCVERLRVAGAGFNFR
SPHEQAIFEIARTIWRPASDKHGRDSDDEGGGGGNNAHDATFAMDAATFAIDAALAAANGGSAKSPTSRPRR
GRRRRTGDFPADAVPETTLALPPAPMPVMTEDVLPSFPQVTAAAVMDGGCGVSVDPASGLPAALSTAAAAAA
VSGSSTAENPILAALFKEMVHAMLSVGVGGSTALLGLEPPPPIAGVPMEGEKWRQQRIQELEVYLRRIDLLQDQ
ARAALDELRSAPHAGGMNT
>Z.mays GRMZM2G030458_P01
MFPTVDEPSAAGAMVSSSFPDADAYGNGDSDGLDFPVDPNPNPVFSSPAAAAAPGSAGAAGERRQLFQRLW
TDEDEIVILRAFAEFTAQRGTSFASHQYDTTPFYEDMRRRLNTGFTKNQLAEKIRRLKRKYRNCVERLRVAGSGF
TFRSPHEQAIFEIARTIWRPASDKHGRDSDDEGGGGNVHDATFAPFAIDAAANGESAKSSRPRRGRRRRTGDFP
ADAVALPPAPMPMQVMTEDALPSFPQATAAAVMNGGCGLIVDPASGLPTALSSAAAAATAVSGGSTTENPILT
ALFKEMVRAMLSVVGSGGGTTALQGLEPPPPIAGVPMEGEKWRQQRIRELEVYLRRMDLLQDQARAALEELR
SAPHAGGMNT
>Z.mays GRMZM2G036966_P01
MAPSSPQLKEPSSAVKQASTGPAALPERTKRKTSPSLPEASGATQQPAKRKPPPFQRAWSPGDEVRILKALATYR
REHGGRLPTPVELFGALDGSLEKEGVGAKELAIKQRSFKRRYDRDVMKNAPPADKHERRLYLLSKHVWGRVPP
PRPPVAKVGSATQAKGAGAQASKADAQAQAQAKSADHQSGKEALKPKAKTLDEMRELYPYLVDEATILVEPAV
LERVLLNIGDIDAQVLDKKIRKARKQLADAITESARINNMEVPTIFLFTSSKLQPEKLRVENENNLLVDHLEKMDD
VDICAKQRLARVEREVKELRQTVIAYQSQAKGIICESAKGGLQSIVAENQTPANILQKKTEVPNDLIHKKNAAVTS
KYHCAVPHNVPPNKPKMQGVMLPPSKAFPGMPRKIDKSPTPRKAIWKGPIC
>Z.mays GRMZM2G036966_P02
MAPSSPQLKEPSSAVKQASTGPAALPERTKRKTSPSLPEASGATQQPAKRKPPPFQRAWSPGDEVRILKALATYR
REHGGRLPTPVELFGALDGSLEKEGVGAKELAIKQRSFKRRYDRDVMKNAPPADKHERRLYLLSKHVWGRVPP
PRPPVAKVGSATQAKGAGAQASKADAQAQAQAKSADHQSGKEALKPKAKTLDEMRELYPYLVDEATILVEPAV
LERVLLNIGDIDAQVLDKKIRKARKQLADAITESARINNMEVPTIFLFTSSKLQPEKLRVENENNLLVDHLEKMDD
VDICAKQRLARVEREVKELRQTVIAYQSQAKGIICESAKGGLQSIVAENQTPANILQKKTEVPNDLIHKKNAAVTS
KYHCAVPHNVPPNKPKMQGVMLPPSKAFPVGGKEHDSRDLVFVDKAKLLEGEYVVCRGKVRQNRVVGDDVS
SVGEVRVEDLKIAPLRAGVLDP
>Z.mays GRMZM2G041818_P01
MASDQQALPVPVPVPVPVPPNPNPTAPADLTPPSASAARKLPIKRRSPRPSSSPPSSSSPASSDPLRAPGGGGGSD
QQQPPFKFQRIWSESDELRFLQGLLGCGTQGLVFPRDLNVFYDRFSESMPQPYTRAQLSEKLRRLKNKFRSMS
ARVAGGIDPARLAPHDRDVLHLCSRLWDPANAATSPFAASAGTSGNKRRRSNPRGTPPDASGDSNSHDYNGIS

SSAPGLFPDGSNGEDTFYLEQESGQLGNHEGAALVADSKFDVVVQEQPEAVVTLPNGNNGIGSEMSAECKTV
VPCSNEHRMANAVLDVFEECLREAKSNGIINGGNVESELSKRWRAQRMDELDVLSRRLRLLVEYAAAAGH
>Z.mays GRMZM2G066373_P01
MPSKRPSPHAMDEAASATAAAAAATARLRPSTPRSKKRTSRSKSRGRRRSPNPNPSSRREGSADHASAAPSRKS
DRKPKPRSFPDSATLATAMASVSAAAAAAPSSGAGRGSAGAVQKLWSEADEIALLTGAAAFKDRTGIAPRLPD
MGELFDSIRDSLAPHLDQAKVYYKLKRLKSKFQHSVPGDSSTAHEHRLRDLGAALWGAELTRPEENAAEEAAD
ADDIDGNREGAVKLPMVKEVLGEYWRLNGQTMSGVSLEKGLALLGSQEASDTEVKWRRQLEADMRMQMR
RHDLEKEVYGLLIDAIKGLGP
>Z.mays GRMZM2G075122_P01
MPPTGDDPSAAAGISFPDAGGGGDSEDGDFAGSHLLDPTDPGLPNPTTSSATGLPHAIPAAGSGGGPVTSGN
GGERRPLFQRLWTEEDEIVILRGFAEFTAARGTAFASHQYDTDPFYEDMRRRLQLDFSKSQLVEKLRRLKRKYRN
CVSRLRESGATFTFRSPHEQAIFEIARNIWRPAKQTRPRSLG
>Z.mays GRMZM2G075122_P02
MPPTGDDPSAAAGISFPDAGGGGDSEDGDFAGSHLLDPTDPGLPNPTTSSATGLPHAIPAAGSGGGPVTSGN
GGERRPLFQRLWTEEDEIVILRGFAEFTAARGTAFASHQYDTDPFYEDMRRRLQLDFSKSQLVEKLRRLKRKYRN
CVSRLRESGATFTFRSPHEQAIFEIARNIWRPANKHGRDPSADSDDEDAAAAAATQIPANTSPNGEVRSPSSAR
QRRRRRATEFTPATNMLQPPQPVPLSVSVPVKTDDSLPAQPQTPMPTMDGSEPVRLPVVSPQSGVSDAEKTCL
TPLFREIIHAAMNVGANPFGAKLPEPPLGLPLPMEGEKWRRQRILELEVYLKRIELLQDQAKATLEELKSSTPGT
>Z.mays GRMZM2G082318_P01
MPPTGDDPPASAGISFPDADGGGDSEDGDFAGAHLLDPTDPGLPNPTTSSATGLPHAIPAAGSGGGPVTSGN
GGERRPLFQRLWTEEDEIVILRGFAEFTAARGTAFASHQYDTDPFYEDMRRRLQLDFTKSQLVEKLRRLKRKYRN
CVSRLRESGATFTFRSPHEQAIFEIARNIWRPTNKHGRDPSADSDDEDAAAATQIPANTSPNGEAKSPSSTRQR
RRRRVMEFSPATGAAPATNMLQPPQPVQLSVSVPVKMDDSLPAQPQTPMPVMVAMDGSEPLRLPVVSPQS
GVSDAEKTCLTPLFKEMLRAAINVGANPFGAKLPEPPLGLAMEGEKWRKQRILELEVYLKRIELLQDQAKATLEE
LKSSTPGS
>Z.mays GRMZM2G083886_P01
MAPKRSAPPPPPPPPAASSEETASGSGSEEEEEEEEEEEEDDLETAHSPPPVAPKSVAPLPQKVQEPEASDEDEY
DDEDDEPQKVQEPEASDEDEDDEEEDIEEDEKANHVVPSSATKNPPPPPQTGEDSEASDEEEDREADDEMPQ
TKPAPNQEVEAKGAKRPSAPFQRTWSIDDDFRILEALAAQRLEHGALPQTDVLADALAGKLDNSGCSLSDLKRK
VRSLQSRYAKAVKKGAPPSKDQDRRLFDLCKNVWPSVSNAKPVTKAVTKASANGGAGREPDEMCELYPYLAEE
VRALQRAHPGLFKREFGMIEDSKARTLDERIKKQRRALMNLHLRRHDLTKEVTRTLMDLAE
>Z.mays GRMZM2G092409_P01
MAPKRPAAAAAAAASGSASEASDAEADVGHQPSSPSPSKTPPTNPNPKSAAAGPAEDSTAAGSDSEAAYDSDA
DHRPAHRKAAVAASPSRKPRSPRPRSRSRSPDAASYSDGAASDADADPAARDGADSADDGNASPLALPRLSRA
EAAAIKPLSSRPMDPPRRSMVHSFSELRSKRPRSAAVPSSVEQLKRPTRLWSLRDEIVILRGLVTYRAKRGVLPGA
MYDISMFQGHIQSELSVNVTPTQLSDKVRRLKQKYNQLASRGKNGRDPDLPTQHEQSVYEIGKKVWGTSTGG
DGYEIGGDSQEEHEIGESDEDVESGWDERARKNRRLMPITMANGHGSGFGAGRGKFDVEKGKDAYPYLWET
VEDLSKEHPNGVAFKKAFELIEGARARGMEEKLRKFRLTEIRHQLRRMELMKETVKMVLDALEG
>Z.mays GRMZM2G125239_P01
MARKRRAPSPPPPPQEESSSEESGSEELEEPPRLPELRSPPQAAANNADSSEADTDSDTDAQAFQMRQVPRSL
TKHTHPVPQPESDPEEEEGESSESEHENPVPVVQKKAAAAAVLSKAEQERKRPPSDPAPTGKAKKMKAVATPL
AKAKKGKAELEKPAPEATPAGNAKKGKAELEKPTFDAIPAGKAKKGKAKEAAPEATPSGKGKKDGDKLEKSAALD
PSHSDSKSEKPVRPTWGKDDEMKILEALAAHVKREGELPKTDLLLASVRDHLVRKNCTYSDIYEKVRRLKERYKK
AASTGIVPSKEDGLQMYNLSEAIWGEKAMEAAAAVTSQKGGAVIKGKKGQANKEKMLENAKSDVTKESAPST

ANQSGDSQKASKKGQARLSEEATTTASPSKSKKQESHNGELNKDDGNLAKGKKGKTDKGKMDIDMDRLTPKE
PVNANQNGGIVIRSKEGEINDEGTEGDANVQGVHRRFGELQKLYSNLAVYVEEIETHHPCGETLKRVFEFIADEK
AEDLESKIKKLRVAQAKAEVRQGNIKKEVLNLMLSLVD
>Z.mays GRMZM2G125239_P02
MARKRRAPSPPPPPQEESSSEESGSEELEEPPRLPELRSPPQAAANNADSSEADTDSDTDAQAFQMRQVPRSL
TKHTHPVPQPESDPEEEEGESSESEHENPVPVVQKKAAAAAVLSKAEQERKRPPSDPAPTGKAKKMKAVATPL
AKAKKGKAELEKPAPEATPAGNAKKGKAELEKPTFDAIPAGKAKKGKAKEAAPEATPSGKGKKDGDKLEKSAALD
PSHSDSKSEKPVRPTWGKDDEMKILEALAAHVKREGELPKTDLLLASVRDHLVRKNCTYSDIYEKVRRLKERYKK
AASTGIVPSKEDGLQMYNLSEAIWGEKAMEAAAAVTSQKGGAVIKGKKGQANKEKMLENAKSDVTKESAPST
ANQSGDSQKASKKGQARLSEEATTTASPSKSKKQESHNGELNKDDGNLAKGKKGKTDKGKMDIDMDRLTPKE
PVNANQNGGIVIRSKEGEINDEGTEGDANVQGVHRRFGELQKLYSNLAVYVEEIETHHPCGETLKRVFEFIADEK
AEDLESKIKKLRVAQAKAEVRQGNIKKEVLNLMLSLVD
>Z.mays GRMZM2G125239_P03
MARKRRAPSPPPPPQEESSSEESGSEELEEPPRLPELRSPPQAAANNADSSEADTDSDTDAQAFQMRQVPRSL
TKHTHPVPQPESDPEEEEGESSESEHENPVPVVQKKAAAAAVLSKAEQERKRPPSDPAPTGKAKKMKAVATPL
AKAKKGKAELEKPAPEATPAGNAKKGKAELEKPTFDAIPAGKAKKGKAKEAAPEATPSGKGKKDGDKLEKSAALD
PSHSDSKSEKPVRPTWGKDDEMKILEALAAHVKREGELPKTDLLLASVRDHLVRKNCTYSDIYEKVRRLKERYKK
AASTGIVPSKEDGLQMYNLSEAIWGEKAMEAAAAVTSQKGGAVIKGKKGQANKEKMLENAKSDVTKESAPST
ANQSGDSQKASKKGQARLSEEATTTASPSKSKKQESHNGELNKDDGNLAKGKKGKTDKGKMDIDMDRLTPKE
PVNANQNGGIVIRSKEGEINDEGTEGDANVQGVHRRFGELQKLYSNLAVYVEEIETHHPCGETLKRVFEFIADEK
AEDLESKIKKLRVAQAKAEVRQGNIKKEVLNLMLSLVD
>Z.mays GRMZM2G134866_P01
MAPKRPAAASGSASEASDAEADAGHHQPSSPSPSKTPPPPNPNPKSTAAGPTSAAEDSTAAGSGSEAAYDSDA
DHRTAPRNAASPSSKPRSTRPRSRSRSRSPDAASYSDGVASDADADPAAVDGADSADDGNASPLAPPRPSRAE
AAAVKPISSRPMDPPRRSMVPSFSEQRSKRPRSVAVPSSVEHLKRPTRLWSLHDEIVILRGLATYRAKRGVLPGS
TYDISKLHGRIRSELSVKVTTTQLGDKVRRLKQKYNLLASRGKSGRDPDLPSQHEQSVYEIGKKVWGGSANGGG
STGGDGYEIGGDSEEEHEFRESDEDVENGRDERARKNRRVMPIAMANGNGAGLGAVKANSRGKFDTEKGKD
AYPYLWETVEDLSKEHPNGVAFKKAFESIEGPKARGMEEKLRKFRLTEIRHQLRRMELMKETVKMVLDALEG
>Z.mays GRMZM2G134866_P02
MAPKRPAAASGSASEASDAEADAGHHQPSSPSPSKTPPPPNPNPKSTAAGPTSAAEDSTAAGSGSEAAYDSDA
DHRTAPRNAASPSSKPRSTRPRSRSRSRSPDAASYSDGVASDADADPAAVDGADSADDGNASPLAPPRPSRAE
AAAVKPISSRPMDPPRRSMVPSFSEQRSKRPRSVAVPSSVEHLKRPTRLWSLHDEIVILRGLATYRAKRGVLPGS
TYDISKLHGRIRSELSVKVTTTQLGDKVRRLKQKYNLLASRGKSGRDPDLPSQHEQSVYEIGKKVWGGSANGGG
STGGDGYEIGGDSEEEHEFRESDEDVENGRDERARKNRRVMPIAMANGNGAGLGAVKANSRGKFDTEKGKD
AYPYLWETVEDLSKEHPNGVAFKKAFESIEGPKARGMEEKLRKFRLTEIRHQLRRMELMKETVKMVLDALEG
>Z.mays GRMZM2G155252_P01
MAAKRRARQPRTEDSKELGLNGGNEGSDEEEEESSKSDALKRAEQPSPPSPEDAEAERSGEASPQRSKPRGWV
AMKTKTTTSKRVKKPSPASEKEYEAGDSGDLSPASDPLPDNIVDAGDNKMPATLKPGKKSSPTPQQEEERPEKR
AAGSDGETVATSSPQRKKKSKRSVDKDRRKKNRNQIRKALTQEDEDHDTVMAEQEQSNTSLPQDEHAAREEE
QEEKMAQQEHAGDTSHPHDKDMRSAHGEEQQDANQEHADDTSPAEDNHSVDEEEQQVEPIPQQEQPNDT
SPPQEKNRAQEDEETGAQVNGKALEGRNAPSTKLRSFESEKKPAVERSWSYDDELKILNALVEHAQSHNGSLP
DSSHVLANLTFDKIDANEDKLTDKIRKLRTRYRRWLLQGYTSSVSGSQLFKLSEILWGQADEDEQVEPTSRDFSV
VSKLYPHLAKEVKAYAETHSSGDLIMGMVMTICDKKARDLDAKCKKQQIETFKLELGQANLTNELLSALSSQTHV
KF

>Z.mays GRMZM2G162405_P01
MSPPPPSGRAAPCPTQPPRHFPPVPSRSRRTSPRREGGARSLDPCAPASAMASDQQALSVPVPVPVPSNPNP
SALADPTPPSASAPRKLPIKRRSPRPSSSPPSSSPDSSDPLRAPAAGGGGGGSDKQQQPPFKFQRIWSESDELRF
LQGLLGCGAQGLVFPRDLNVFYDRFSESMPQPYTRAQLSEKLRRLKNKFRSMSARVAEGLDPARLAPHDRDVL
HLCSRLWDPANAATSPFVASAGTSGNKRRRANLQGTSLPPPDASGDSNSHDYNGINSSAPCLFPDGSNGEDM
FYLEQEPGHLGDHEGAAPVADSRFGVIVQEQGVIVHEQGEAVGTLPKWEQWNWQ
>Z.mays GRMZM2G162405_P02
MASDQQALSVPVPVPVPSNPNPSALADPTPPSASAPRKLPIKRRSPRPSSSPPSSSPDSSDPLRAPAAGGGGGG
SDKQQQPPFKFQRIWSESDELRFLQGLLGCGAQGLVFPRDLNVFYDRFSESMPQPYTRAQLSEKLRRLKNKFRS
MSARVAEGLDPARLAPHDRDVLHLCSRLWDPANAATSPFVASAGTSGNKRRRANLQGTSLPPPDASGDSNSH
DYNGINSSAPCLFPDGSNGEDMFYLEQEPGHLGDHEGAAPVADSRFGVIVQEQGVIVHEQGEAVGTLPNGNN
GIGNEMNVECKMVVPCSNEHRMANAVLDVFEECLREAKSNGVINGGNVDGSGEESELSKRWRAQRMDELD
VLSRRLRLLVEYAAAAGQ
>Z.mays GRMZM2G174240_P01
MSSGRPSEPAMNAGATPAASSPPRSKKRPSRMGDGNSSPNPSPRGSPARRSELKRRRRTSAESAALALATVAA
RASAAGVRSDAGDGAVERRRRPRTRAEGGDGAIRMLGAAAARVGGGRGAAQKHWSDDDEFTLLTAAVAFR
DRNGRAPRLPDMAELFESIRESISPHIDQFMVYYKIKRLKSKFQHSAGPRDRRLRDLCSVLWGVGVVASSEDDS
DVGERRTVPDAAAMMPVVTEVLGEYWKTNERAMAGVSLEKGLSLLGKKEGRLIETKWRQQLDQEMQSQM
RRHDLAKEVCGLLTDAIKGLGP
>Z.mays GRMZM2G392516_P01
MASSSPQLKEPSSAVKRASTGPTALPERNKRKTSPSLPEASGATQQPAKRKPPLFQRAWSPGDEVRILEALATYR
REHGGRLPTPVELFGALDGSLEKEGVGAKELAIKQRSLKRRYDRDVMKNAPPADKHERRLYLLSKHVWGRVPP
TRPPVAKVRSATQAKCAGAQASNAAAQAQAKSADRQSGKEALKPKAKTLDEMRELYPYLVDEATILVEPAVLER
VLLNIEDIDAQVLDKKIRKARKQLAGAITESARINNMEMPTIFLFTSSKLQPEKLRVENDNNLLVDHLEKMDDVD
ICAKQRLARVEHEVMELRQTVIAYQAQAKVESIPRNNISSVKGIICESAKSGLQSIVAENQTPANVLQKKTEVPNG
LIHKKNAAVTSKYYCAVPHNVPPNKPKMQGVMLPPFKVFPVGGKEHDARDLVFIDKAKLLEREYVACHGKVRQ
HRVVGDDVSSVGEVRVEDLKIAPLRAGVLDP
>Z.mays GRMZM2G423292_P01
MALKRSAAAASLGSASDASDGEAGAARNSHRRRVSPSRSRSESPRPNLGPSAAASDTNRERSPRDHLDSDADS
DADAGAGAGRGARSSRLLGERSPSFHSNSDADVAGRSPSPRRNRERTLHLHSDSDSDNSTTAAAAASDDAGD
ASPLAIAHRASHIGTSNVKPVSTRPMDVPSRAAAGSSQRRSKRRPSPHSPEHQKRPPRVWSPEDEVTILSALIEF
RAKKGRLPASIQDTSKVHSQIIHQLTANASTAQLSDKVRRLKHKYKLLLTRSKNGRDPDLPTKHDRDVYQLSKKV
WGLKSLLLVRGSRVSHEDTEDAEESNEEQEIEESDEDIENGWGLRGRTGKKPRALTFENGNGNAIVTAGRACH
GDGSGRDDAEKGKQMYPYLWEAVEELSKEHPSGPIFRKAFGVLEKSKAGAVEEKLRKFRMLEIRQQLRRMDL
MKETVGLVLDALEGAY
>Z.mays GRMZM5G868875_P01
MTSEEHPSSASSDGSTTIGSDEHLDPSPSPSTSFDSPPESDDSAASSLLSDGSSESEPIIFKKPRPPVRSWPASDEI
AILETVASHRQKHGRLPLADDLAAAVRGRLSVGDRLSAAQITRRLCALRNRYDNTVIRLKRGTIPAKDSDVTIYRL
SKLIWEGTRRGRIEKKTRVPDARNDPRGFDDLAELYPCLSAEVEAIGARCGYGALKRAFGRIGDDTAARLEAKLK
RQRMAETRASGKLDRLRSRVANALQGFNK

>Z.mays GRMZM5G868875_P02

MTSEEHPSSASSDGSTTIGSDEHLDPSPSPSTSFDSPPESDDSAASSLLSDGSSESEPIIFKKPRPPVRSWPASDEI
AILETVASHRQKHGRLPLADDLAAAVRGRLSVGDRLSAAQITRRLCALRNRYDNTVIRLKRGTIPAKDSDVTIYRL
SKLIWEGTRRGRIEKKTRVPDARNDPRGFDDLAELYPCLSAEVEAIGARCGYGALKRAFGRIGDDTAARLEAKLK
RQRMAETRASGKLDRLRSRVANALQGFNK
