# Supplementary material for: Genome-Wide Identification and Expression Profiling of SlGeBP Gene Family in Response to Hormone and Abiotic Stresses in Solanum lycopersicum L
Source: Int J Mol Sci. 2025 Jun 23;26(13):6008. doi: 10.3390/ijms26136008 (PMC12250332; doi:10.3390/ijms26136008)
Supplement: Supplementary file 1 [file ijms-26-06008-s001.zip › Table S7 Primers used in this study.pdf]

Name/ID	Primer	Sequence (5’-3’)	Application
Solyc01g097890	q*SlGeBP1*-F	ACTTCGCCATTCCGGTCATT	qRT-PCR
	q*SlGeBP1*-R	TCCACGCAACAGCTCAATCT	
Solyc02g005290	q*SlGeBP2*-F	TTTTGTCTAGGTTACGAGAAAGTGT	
	q*SlGeBP2*-R	AAAGATGGTGGAATGGTGAGTCTTC	
Solyc02g014740	q*SlGeBP3*-F	CTCCCAAGCCCAAGAGGAAG	
	q*SlGeBP3*-R	ACACGCGTAGGTTGAAGAGT	
Solyc02g014750	q*SlGeBP4*-F	CCACAGGTGCAGCAAATTGTA	
	q*SlGeBP4*-R	GGGCTTGGGAGAAGGAGTTT	
Solyc02g083750	q*SlGeBP5*-F	TTCCACTTCCTCTCCTCCCG	
	q*SlGeBP5*-R	ATCGTTGATCCGCTCTCGTT	
Solyc05g051330	q*SlGeBP6*-F	GGGTCGAACTGTAATGGGGT	
	q*SlGeBP6*-R	ACTCGAACCGGACCATTCAC	
Solyc07g052760	q*SlGeBP7*-F	ACCAGTCAAAACCGCTCAGAA	
	q*SlGeBP7*-R	GCTGATTTCGATGGGGCAGA	
Solyc07g052830	q*SlGeBP8*-F	CATTCCCTGCCTTCCCCTTC	
	q*SlGeBP8*-R	GCGGCCTCTTTGCTACTGAT	
Solyc07g052900	q*SlGeBP9*-F	GAAGAGAATTTGGGGCGCT	
	q*SlGeBP9*-R	TACGGCAATCTGTTTCTCTTCT	
Solyc07g063840	q*SlGeBP10*-F	TCCGCCCTCGATTGAACTAC	
	q*SlGeBP10*-R	TCCATCGAGTTCCCAAGCAT	
Solyc07g064000	q*SlGeBP11*-F	ATCCCCGAATGGATCGACCT	
	q*SlGeBP11*-R	TCTCGGGATTACCTTCGTTCC	
Solyc01g056940	q*UBI*-F	GCCGACTACAACATCCAGAAGG	
	q*UBI*-R	TGCAACACAGCGAGCTTAACC	
Solyc01g097890	*SlGeBP1*-GFP-F	CATTCTACAACTACATCTAGA ATGGATTCTGTACCAAATCG	Subcellular localization
	SlGeBP1-GFP-R	GACCGGCCGGTGGATCCCGGG AGTGTTACTTCCCATTGATC	
Solyc02g083750	*SlGeBP5*-GFP-F	CATTCTACAACTACATCTAGA ATGGCTTCCGTAGAAGATCA	
	SlGeBP5-GFP-R	GACCGGCCGGTGGATCCCGGG ACTGTTCATGGATCTGAGTT	
Solyc01g097890	*SlGeBP1-pAD*-F	TGGCCATGGAGGCCAGTGAATTC ATGGATTCTGTACCAAATCG	Y2H
	SlGeBP1-pAD-R	CTGCAGCTCGAGCTCGATGGATCC AGTGTTACTTCCCATTGATC	

Solyc02g083750	*SlGeBP5-pBK*-F	CATATGGCCATGGAGGCCGAATTC ATGGCTTCCGTAGAAGATCA
	SlGeBP5-pBK-R	TGCGGCCGCTGCAGGTCGACGGATCC ACTGTTCATGGATCTGAGTT
